# Measurement of the differential cross-sections of prompt and non-prompt production of $J/\psi$ and $\psi (2\mathrm {S})$ in *pp* collisions at $\sqrt{s} = 7$ and 8 TeV with the ATLAS detector

**DOI:** 10.1140/epjc/s10052-016-4050-8

**Published:** 2016-05-20

**Authors:** G. Aad, B. Abbott, J. Abdallah, O. Abdinov, R. Aben, M. Abolins, O. S. AbouZeid, H. Abramowicz, H. Abreu, R. Abreu, Y. Abulaiti, B. S. Acharya, L. Adamczyk, D. L. Adams, J. Adelman, S. Adomeit, T. Adye, A. A. Affolder, T. Agatonovic-Jovin, J. Agricola, J. A. Aguilar-Saavedra, S. P. Ahlen, F. Ahmadov, G. Aielli, H. Akerstedt, T. P. A. Åkesson, A. V. Akimov, G. L. Alberghi, J. Albert, S. Albrand, M. J. Alconada Verzini, M. Aleksa, I. N. Aleksandrov, C. Alexa, G. Alexander, T. Alexopoulos, M. Alhroob, G. Alimonti, L. Alio, J. Alison, S. P. Alkire, B. M. M. Allbrooke, P. P. Allport, A. Aloisio, A. Alonso, F. Alonso, C. Alpigiani, A. Altheimer, B. Alvarez Gonzalez, D. Álvarez Piqueras, M. G. Alviggi, B. T. Amadio, K. Amako, Y. Amaral Coutinho, C. Amelung, D. Amidei, S. P. Amor Dos Santos, A. Amorim, S. Amoroso, N. Amram, G. Amundsen, C. Anastopoulos, L. S. Ancu, N. Andari, T. Andeen, C. F. Anders, G. Anders, J. K. Anders, K. J. Anderson, A. Andreazza, V. Andrei, S. Angelidakis, I. Angelozzi, P. Anger, A. Angerami, F. Anghinolfi, A. V. Anisenkov, N. Anjos, A. Annovi, M. Antonelli, A. Antonov, J. Antos, F. Anulli, M. Aoki, L. Aperio Bella, G. Arabidze, Y. Arai, J. P. Araque, A. T. H. Arce, F. A. Arduh, J-F. Arguin, S. Argyropoulos, M. Arik, A. J. Armbruster, O. Arnaez, V. Arnal, H. Arnold, M. Arratia, O. Arslan, A. Artamonov, G. Artoni, S. Asai, N. Asbah, A. Ashkenazi, B. Åsman, L. Asquith, K. Assamagan, R. Astalos, M. Atkinson, N. B. Atlay, K. Augsten, M. Aurousseau, G. Avolio, B. Axen, M. K. Ayoub, G. Azuelos, M. A. Baak, A. E. Baas, M. J. Baca, C. Bacci, H. Bachacou, K. Bachas, M. Backes, M. Backhaus, P. Bagiacchi, P. Bagnaia, Y. Bai, T. Bain, J. T. Baines, O. K. Baker, E. M. Baldin, P. Balek, T. Balestri, F. Balli, W. K. Balunas, E. Banas, Sw. Banerjee, A. A. E. Bannoura, H. S. Bansil, L. Barak, E. L. Barberio, D. Barberis, M. Barbero, T. Barillari, M. Barisonzi, T. Barklow, N. Barlow, S. L. Barnes, B. M. Barnett, R. M. Barnett, Z. Barnovska, A. Baroncelli, G. Barone, A. J. Barr, F. Barreiro, J. Barreiro Guimarães da Costa, R. Bartoldus, A. E. Barton, P. Bartos, A. Basalaev, A. Bassalat, A. Basye, R. L. Bates, S. J. Batista, J. R. Batley, M. Battaglia, M. Bauce, F. Bauer, H. S. Bawa, J. B. Beacham, M. D. Beattie, T. Beau, P. H. Beauchemin, R. Beccherle, P. Bechtle, H. P. Beck, K. Becker, M. Becker, M. Beckingham, C. Becot, A. J. Beddall, A. Beddall, V. A. Bednyakov, C. P. Bee, L. J. Beemster, T. A. Beermann, M. Begel, J. K. Behr, C. Belanger-Champagne, W. H. Bell, G. Bella, L. Bellagamba, A. Bellerive, M. Bellomo, K. Belotskiy, O. Beltramello, O. Benary, D. Benchekroun, M. Bender, K. Bendtz, N. Benekos, Y. Benhammou, E. Benhar Noccioli, J. A. Benitez Garcia, D. P. Benjamin, J. R. Bensinger, S. Bentvelsen, L. Beresford, M. Beretta, D. Berge, E. Bergeaas Kuutmann, N. Berger, F. Berghaus, J. Beringer, C. Bernard, N. R. Bernard, C. Bernius, F. U. Bernlochner, T. Berry, P. Berta, C. Bertella, G. Bertoli, F. Bertolucci, C. Bertsche, D. Bertsche, M. I. Besana, G. J. Besjes, O. Bessidskaia Bylund, M. Bessner, N. Besson, C. Betancourt, S. Bethke, A. J. Bevan, W. Bhimji, R. M. Bianchi, L. Bianchini, M. Bianco, O. Biebel, D. Biedermann, S. P. Bieniek, M. Biglietti, J. Bilbao De Mendizabal, H. Bilokon, M. Bindi, S. Binet, A. Bingul, C. Bini, S. Biondi, D. M. Bjergaard, C. W. Black, J. E. Black, K. M. Black, D. Blackburn, R. E. Blair, J.-B. Blanchard, J. E. Blanco, T. Blazek, I. Bloch, C. Blocker, W. Blum, U. Blumenschein, G. J. Bobbink, V. S. Bobrovnikov, S. S. Bocchetta, A. Bocci, C. Bock, M. Boehler, J. A. Bogaerts, D. Bogavac, A. G. Bogdanchikov, C. Bohm, V. Boisvert, T. Bold, V. Boldea, A. S. Boldyrev, M. Bomben, M. Bona, M. Boonekamp, A. Borisov, G. Borissov, S. Borroni, J. Bortfeldt, V. Bortolotto, K. Bos, D. Boscherini, M. Bosman, J. Boudreau, J. Bouffard, E. V. Bouhova-Thacker, D. Boumediene, C. Bourdarios, N. Bousson, A. Boveia, J. Boyd, I. R. Boyko, I. Bozic, J. Bracinik, A. Brandt, G. Brandt, O. Brandt, U. Bratzler, B. Brau, J. E. Brau, H. M. Braun, S. F. Brazzale, W. D. Breaden Madden, K. Brendlinger, A. J. Brennan, L. Brenner, R. Brenner, S. Bressler, K. Bristow, T. M. Bristow, D. Britton, D. Britzger, F. M. Brochu, I. Brock, R. Brock, J. Bronner, G. Brooijmans, T. Brooks, W. K. Brooks, J. Brosamer, E. Brost, J. Brown, P. A. Bruckman de Renstrom, D. Bruncko, R. Bruneliere, A. Bruni, G. Bruni, M. Bruschi, N. Bruscino, L. Bryngemark, T. Buanes, Q. Buat, P. Buchholz, A. G. Buckley, S. I. Buda, I. A. Budagov, F. Buehrer, L. Bugge, M. K. Bugge, O. Bulekov, D. Bullock, H. Burckhart, S. Burdin, C. D. Burgard, B. Burghgrave, S. Burke, I. Burmeister, E. Busato, D. Büscher, V. Büscher, P. Bussey, J. M. Butler, A. I. Butt, C. M. Buttar, J. M. Butterworth, P. Butti, W. Buttinger, A. Buzatu, A. R. Buzykaev, S. Cabrera Urbán, D. Caforio, V. M. Cairo, O. Cakir, N. Calace, P. Calafiura, A. Calandri, G. Calderini, P. Calfayan, L. P. Caloba, D. Calvet, S. Calvet, R. Camacho Toro, S. Camarda, P. Camarri, D. Cameron, R. Caminal Armadans, S. Campana, M. Campanelli, A. Campoverde, V. Canale, A. Canepa, M. Cano Bret, J. Cantero, R. Cantrill, T. Cao, M. D. M. Capeans Garrido, I. Caprini, M. Caprini, M. Capua, R. Caputo, R. Cardarelli, F. Cardillo, T. Carli, G. Carlino, L. Carminati, S. Caron, E. Carquin, G. D. Carrillo-Montoya, J. R. Carter, J. Carvalho, D. Casadei, M. P. Casado, M. Casolino, E. Castaneda-Miranda, A. Castelli, V. Castillo Gimenez, N. F. Castro, P. Catastini, A. Catinaccio, J. R. Catmore, A. Cattai, J. Caudron, V. Cavaliere, D. Cavalli, M. Cavalli-Sforza, V. Cavasinni, F. Ceradini, B. C. Cerio, K. Cerny, A. S. Cerqueira, A. Cerri, L. Cerrito, F. Cerutti, M. Cerv, A. Cervelli, S. A. Cetin, A. Chafaq, D. Chakraborty, I. Chalupkova, P. Chang, J. D. Chapman, D. G. Charlton, C. C. Chau, C. A. Chavez Barajas, S. Cheatham, A. Chegwidden, S. Chekanov, S. V. Chekulaev, G. A. Chelkov, M. A. Chelstowska, C. Chen, H. Chen, K. Chen, L. Chen, S. Chen, X. Chen, Y. Chen, H. C. Cheng, Y. Cheng, A. Cheplakov, E. Cheremushkina, R. Cherkaoui El Moursli, V. Chernyatin, E. Cheu, L. Chevalier, V. Chiarella, G. Chiarelli, G. Chiodini, A. S. Chisholm, R. T. Chislett, A. Chitan, M. V. Chizhov, K. Choi, S. Chouridou, B. K. B. Chow, V. Christodoulou, D. Chromek-Burckhart, J. Chudoba, A. J. Chuinard, J. J. Chwastowski, L. Chytka, G. Ciapetti, A. K. Ciftci, D. Cinca, V. Cindro, I. A. Cioara, A. Ciocio, F. Cirotto, Z. H. Citron, M. Ciubancan, A. Clark, B. L. Clark, P. J. Clark, R. N. Clarke, W. Cleland, C. Clement, Y. Coadou, M. Cobal, A. Coccaro, J. Cochran, L. Coffey, J. G. Cogan, L. Colasurdo, B. Cole, S. Cole, A. P. Colijn, J. Collot, T. Colombo, G. Compostella, P. Conde Muiño, E. Coniavitis, S. H. Connell, I. A. Connelly, V. Consorti, S. Constantinescu, C. Conta, G. Conti, F. Conventi, M. Cooke, B. D. Cooper, A. M. Cooper-Sarkar, T. Cornelissen, M. Corradi, F. Corriveau, A. Corso-Radu, A. Cortes-Gonzalez, G. Cortiana, G. Costa, M. J. Costa, D. Costanzo, D. Côté, G. Cottin, G. Cowan, B. E. Cox, K. Cranmer, G. Cree, S. Crépé-Renaudin, F. Crescioli, W. A. Cribbs, M. Crispin Ortuzar, M. Cristinziani, V. Croft, G. Crosetti, T. Cuhadar Donszelmann, J. Cummings, M. Curatolo, J. Cúth, C. Cuthbert, H. Czirr, P. Czodrowski, S. D’Auria, M. D’Onofrio, M. J. Da Cunha Sargedas De Sousa, C. Da Via, W. Dabrowski, A. Dafinca, T. Dai, O. Dale, F. Dallaire, C. Dallapiccola, M. Dam, J. R. Dandoy, N. P. Dang, A. C. Daniells, M. Danninger, M. Dano Hoffmann, V. Dao, G. Darbo, S. Darmora, J. Dassoulas, A. Dattagupta, W. Davey, C. David, T. Davidek, E. Davies, M. Davies, P. Davison, Y. Davygora, E. Dawe, I. Dawson, R. K. Daya-Ishmukhametova, K. De, R. de Asmundis, A. De Benedetti, S. De Castro, S. De Cecco, N. De Groot, P. de Jong, H. De la Torre, F. De Lorenzi, D. De Pedis, A. De Salvo, U. De Sanctis, A. De Santo, J. B. De Vivie De Regie, W. J. Dearnaley, R. Debbe, C. Debenedetti, D. V. Dedovich, I. Deigaard, J. Del Peso, T. Del Prete, D. Delgove, F. Deliot, C. M. Delitzsch, M. Deliyergiyev, A. Dell’Acqua, L. Dell’Asta, M. Dell’Orso, M. Della Pietra, D. della Volpe, M. Delmastro, P. A. Delsart, C. Deluca, D. A. DeMarco, S. Demers, M. Demichev, A. Demilly, S. P. Denisov, D. Derendarz, J. E. Derkaoui, F. Derue, P. Dervan, K. Desch, C. Deterre, P. O. Deviveiros, A. Dewhurst, S. Dhaliwal, A. Di Ciaccio, L. Di Ciaccio, A. Di Domenico, C. Di Donato, A. Di Girolamo, B. Di Girolamo, A. Di Mattia, B. Di Micco, R. Di Nardo, A. Di Simone, R. Di Sipio, D. Di Valentino, C. Diaconu, M. Diamond, F. A. Dias, M. A. Diaz, E. B. Diehl, J. Dietrich, S. Diglio, A. Dimitrievska, J. Dingfelder, P. Dita, S. Dita, F. Dittus, F. Djama, T. Djobava, J. I. Djuvsland, M. A. B. do Vale, D. Dobos, M. Dobre, C. Doglioni, T. Dohmae, J. Dolejsi, Z. Dolezal, B. A. Dolgoshein, M. Donadelli, S. Donati, P. Dondero, J. Donini, J. Dopke, A. Doria, M. T. Dova, A. T. Doyle, E. Drechsler, M. Dris, E. Dubreuil, E. Duchovni, G. Duckeck, O. A. Ducu, D. Duda, A. Dudarev, L. Duflot, L. Duguid, M. Dührssen, M. Dunford, H. Duran Yildiz, M. Düren, A. Durglishvili, D. Duschinger, M. Dyndal, C. Eckardt, K. M. Ecker, R. C. Edgar, W. Edson, N. C. Edwards, W. Ehrenfeld, T. Eifert, G. Eigen, K. Einsweiler, T. Ekelof, M. El Kacimi, M. Ellert, S. Elles, F. Ellinghaus, A. A. Elliot, N. Ellis, J. Elmsheuser, M. Elsing, D. Emeliyanov, Y. Enari, O. C. Endner, M. Endo, J. Erdmann, A. Ereditato, G. Ernis, J. Ernst, M. Ernst, S. Errede, E. Ertel, M. Escalier, H. Esch, C. Escobar, B. Esposito, A. I. Etienvre, E. Etzion, H. Evans, A. Ezhilov, L. Fabbri, G. Facini, R. M. Fakhrutdinov, S. Falciano, R. J. Falla, J. Faltova, Y. Fang, M. Fanti, A. Farbin, A. Farilla, T. Farooque, S. Farrell, S. M. Farrington, P. Farthouat, F. Fassi, P. Fassnacht, D. Fassouliotis, M. Faucci Giannelli, A. Favareto, L. Fayard, P. Federic, O. L. Fedin, W. Fedorko, S. Feigl, L. Feligioni, C. Feng, E. J. Feng, H. Feng, A. B. Fenyuk, L. Feremenga, P. Fernandez Martinez, S. Fernandez Perez, J. Ferrando, A. Ferrari, P. Ferrari, R. Ferrari, D. E. Ferreira de Lima, A. Ferrer, D. Ferrere, C. Ferretti, A. Ferretto Parodi, M. Fiascaris, F. Fiedler, A. Filipčič, M. Filipuzzi, F. Filthaut, M. Fincke-Keeler, K. D. Finelli, M. C. N. Fiolhais, L. Fiorini, A. Firan, A. Fischer, C. Fischer, J. Fischer, W. C. Fisher, E. A. Fitzgerald, N. Flaschel, I. Fleck, P. Fleischmann, S. Fleischmann, G. T. Fletcher, G. Fletcher, R. R. M. Fletcher, T. Flick, A. Floderus, L. R. Flores Castillo, M. J. Flowerdew, A. Formica, A. Forti, D. Fournier, H. Fox, S. Fracchia, P. Francavilla, M. Franchini, D. Francis, L. Franconi, M. Franklin, M. Frate, M. Fraternali, D. Freeborn, S. T. French, F. Friedrich, D. Froidevaux, J. A. Frost, C. Fukunaga, E. Fullana Torregrosa, B. G. Fulsom, T. Fusayasu, J. Fuster, C. Gabaldon, O. Gabizon, A. Gabrielli, A. Gabrielli, G. P. Gach, S. Gadatsch, S. Gadomski, G. Gagliardi, P. Gagnon, C. Galea, B. Galhardo, E. J. Gallas, B. J. Gallop, P. Gallus, G. Galster, K. K. Gan, J. Gao, Y. Gao, Y. S. Gao, F. M. Garay Walls, F. Garberson, C. García, J. E. García Navarro, M. Garcia-Sciveres, R. W. Gardner, N. Garelli, V. Garonne, C. Gatti, A. Gaudiello, G. Gaudio, B. Gaur, L. Gauthier, P. Gauzzi, I. L. Gavrilenko, C. Gay, G. Gaycken, E. N. Gazis, P. Ge, Z. Gecse, C. N. P. Gee, Ch. Geich-Gimbel, M. P. Geisler, C. Gemme, M. H. Genest, S. Gentile, M. George, S. George, D. Gerbaudo, A. Gershon, S. Ghasemi, H. Ghazlane, B. Giacobbe, S. Giagu, V. Giangiobbe, P. Giannetti, B. Gibbard, S. M. Gibson, M. Gilchriese, T. P. S. Gillam, D. Gillberg, G. Gilles, D. M. Gingrich, N. Giokaris, M. P. Giordani, F. M. Giorgi, F. M. Giorgi, P. F. Giraud, P. Giromini, D. Giugni, C. Giuliani, M. Giulini, B. K. Gjelsten, S. Gkaitatzis, I. Gkialas, E. L. Gkougkousis, L. K. Gladilin, C. Glasman, J. Glatzer, P. C. F. Glaysher, A. Glazov, M. Goblirsch-Kolb, J. R. Goddard, J. Godlewski, S. Goldfarb, T. Golling, D. Golubkov, A. Gomes, R. Gonçalo, J. Goncalves Pinto Firmino Da Costa, L. Gonella, S. González de la Hoz, G. Gonzalez Parra, S. Gonzalez-Sevilla, L. Goossens, P. A. Gorbounov, H. A. Gordon, I. Gorelov, B. Gorini, E. Gorini, A. Gorišek, E. Gornicki, A. T. Goshaw, C. Gössling, M. I. Gostkin, D. Goujdami, A. G. Goussiou, N. Govender, E. Gozani, H. M. X. Grabas, L. Graber, I. Grabowska-Bold, P. O. J. Gradin, P. Grafström, K.-J. Grahn, J. Gramling, E. Gramstad, S. Grancagnolo, V. Gratchev, H. M. Gray, E. Graziani, Z. D. Greenwood, C. Grefe, K. Gregersen, I. M. Gregor, P. Grenier, J. Griffiths, A. A. Grillo, K. Grimm, S. Grinstein, Ph. Gris, J.-F. Grivaz, J. P. Grohs, A. Grohsjean, E. Gross, J. Grosse-Knetter, G. C. Grossi, Z. J. Grout, L. Guan, J. Guenther, F. Guescini, D. Guest, O. Gueta, E. Guido, T. Guillemin, S. Guindon, U. Gul, C. Gumpert, J. Guo, Y. Guo, S. Gupta, G. Gustavino, P. Gutierrez, N. G. Gutierrez Ortiz, C. Gutschow, C. Guyot, C. Gwenlan, C. B. Gwilliam, A. Haas, C. Haber, H. K. Hadavand, N. Haddad, P. Haefner, S. Hageböck, Z. Hajduk, H. Hakobyan, M. Haleem, J. Haley, D. Hall, G. Halladjian, G. D. Hallewell, K. Hamacher, P. Hamal, K. Hamano, A. Hamilton, G. N. Hamity, P. G. Hamnett, L. Han, K. Hanagaki, K. Hanawa, M. Hance, P. Hanke, R. Hanna, J. B. Hansen, J. D. Hansen, M. C. Hansen, P. H. Hansen, K. Hara, A. S. Hard, T. Harenberg, F. Hariri, S. Harkusha, R. D. Harrington, P. F. Harrison, F. Hartjes, M. Hasegawa, Y. Hasegawa, A. Hasib, S. Hassani, S. Haug, R. Hauser, L. Hauswald, M. Havranek, C. M. Hawkes, R. J. Hawkings, A. D. Hawkins, T. Hayashi, D. Hayden, C. P. Hays, J. M. Hays, H. S. Hayward, S. J. Haywood, S. J. Head, T. Heck, V. Hedberg, L. Heelan, S. Heim, T. Heim, B. Heinemann, L. Heinrich, J. Hejbal, L. Helary, S. Hellman, D. Hellmich, C. Helsens, J. Henderson, R. C. W. Henderson, Y. Heng, C. Hengler, S. Henkelmann, A. Henrichs, A. M. Henriques Correia, S. Henrot-Versille, G. H. Herbert, Y. Hernández Jiménez, R. Herrberg-Schubert, G. Herten, R. Hertenberger, L. Hervas, G. G. Hesketh, N. P. Hessey, J. W. Hetherly, R. Hickling, E. Higón-Rodriguez, E. Hill, J. C. Hill, K. H. Hiller, S. J. Hillier, I. Hinchliffe, E. Hines, R. R. Hinman, M. Hirose, D. Hirschbuehl, J. Hobbs, N. Hod, M. C. Hodgkinson, P. Hodgson, A. Hoecker, M. R. Hoeferkamp, F. Hoenig, M. Hohlfeld, D. Hohn, T. R. Holmes, M. Homann, T. M. Hong, L. Hooft van Huysduynen, W. H. Hopkins, Y. Horii, A. J. Horton, J-Y. Hostachy, S. Hou, A. Hoummada, J. Howard, J. Howarth, M. Hrabovsky, I. Hristova, J. Hrivnac, T. Hryn’ova, A. Hrynevich, C. Hsu, P. J. Hsu, S.-C. Hsu, D. Hu, Q. Hu, X. Hu, Y. Huang, Z. Hubacek, F. Hubaut, F. Huegging, T. B. Huffman, E. W. Hughes, G. Hughes, M. Huhtinen, T. A. Hülsing, N. Huseynov, J. Huston, J. Huth, G. Iacobucci, G. Iakovidis, I. Ibragimov, L. Iconomidou-Fayard, E. Ideal, Z. Idrissi, P. Iengo, O. Igonkina, T. Iizawa, Y. Ikegami, M. Ikeno, Y. Ilchenko, D. Iliadis, N. Ilic, T. Ince, G. Introzzi, P. Ioannou, M. Iodice, K. Iordanidou, V. Ippolito, A. Irles Quiles, C. Isaksson, M. Ishino, M. Ishitsuka, R. Ishmukhametov, C. Issever, S. Istin, J. M. Iturbe Ponce, R. Iuppa, J. Ivarsson, W. Iwanski, H. Iwasaki, J. M. Izen, V. Izzo, S. Jabbar, B. Jackson, M. Jackson, P. Jackson, M. R. Jaekel, V. Jain, K. Jakobs, S. Jakobsen, T. Jakoubek, J. Jakubek, D. O. Jamin, D. K. Jana, E. Jansen, R. Jansky, J. Janssen, M. Janus, G. Jarlskog, N. Javadov, T. Javůrek, L. Jeanty, J. Jejelava, G.-Y. Jeng, D. Jennens, P. Jenni, J. Jentzsch, C. Jeske, S. Jézéquel, H. Ji, J. Jia, Y. Jiang, S. Jiggins, J. Jimenez Pena, S. Jin, A. Jinaru, O. Jinnouchi, M. D. Joergensen, P. Johansson, K. A. Johns, K. Jon-And, G. Jones, R. W. L. Jones, T. J. Jones, J. Jongmanns, P. M. Jorge, K. D. Joshi, J. Jovicevic, X. Ju, C. A. Jung, P. Jussel, A. Juste Rozas, M. Kaci, A. Kaczmarska, M. Kado, H. Kagan, M. Kagan, S. J. Kahn, E. Kajomovitz, C. W. Kalderon, S. Kama, A. Kamenshchikov, N. Kanaya, S. Kaneti, V. A. Kantserov, J. Kanzaki, B. Kaplan, L. S. Kaplan, A. Kapliy, D. Kar, K. Karakostas, A. Karamaoun, N. Karastathis, M. J. Kareem, E. Karentzos, M. Karnevskiy, S. N. Karpov, Z. M. Karpova, K. Karthik, V. Kartvelishvili, A. N. Karyukhin, L. Kashif, R. D. Kass, A. Kastanas, Y. Kataoka, C. Kato, A. Katre, J. Katzy, K. Kawagoe, T. Kawamoto, G. Kawamura, S. Kazama, V. F. Kazanin, R. Keeler, R. Kehoe, J. S. Keller, J. J. Kempster, H. Keoshkerian, O. Kepka, B. P. Kerševan, S. Kersten, R. A. Keyes, F. Khalil-zada, H. Khandanyan, A. Khanov, A. G. Kharlamov, T. J. Khoo, V. Khovanskiy, E. Khramov, J. Khubua, S. Kido, H. Y. Kim, S. H. Kim, Y. K. Kim, N. Kimura, O. M. Kind, B. T. King, M. King, S. B. King, J. Kirk, A. E. Kiryunin, T. Kishimoto, D. Kisielewska, F. Kiss, K. Kiuchi, O. Kivernyk, E. Kladiva, M. H. Klein, M. Klein, U. Klein, K. Kleinknecht, P. Klimek, A. Klimentov, R. Klingenberg, J. A. Klinger, T. Klioutchnikova, E.-E. Kluge, P. Kluit, S. Kluth, J. Knapik, E. Kneringer, E. B. F. G. Knoops, A. Knue, A. Kobayashi, D. Kobayashi, T. Kobayashi, M. Kobel, M. Kocian, P. Kodys, T. Koffas, E. Koffeman, L. A. Kogan, S. Kohlmann, Z. Kohout, T. Kohriki, T. Koi, H. Kolanoski, I. Koletsou, A. A. Komar, Y. Komori, T. Kondo, N. Kondrashova, K. Köneke, A. C. König, T. Kono, R. Konoplich, N. Konstantinidis, R. Kopeliansky, S. Koperny, L. Köpke, A. K. Kopp, K. Korcyl, K. Kordas, A. Korn, A. A. Korol, I. Korolkov, E. V. Korolkova, O. Kortner, S. Kortner, T. Kosek, V. V. Kostyukhin, V. M. Kotov, A. Kotwal, A. Kourkoumeli-Charalampidi, C. Kourkoumelis, V. Kouskoura, A. Koutsman, R. Kowalewski, T. Z. Kowalski, W. Kozanecki, A. S. Kozhin, V. A. Kramarenko, G. Kramberger, D. Krasnopevtsev, M. W. Krasny, A. Krasznahorkay, J. K. Kraus, A. Kravchenko, S. Kreiss, M. Kretz, J. Kretzschmar, K. Kreutzfeldt, P. Krieger, K. Krizka, K. Kroeninger, H. Kroha, J. Kroll, J. Kroseberg, J. Krstic, U. Kruchonak, H. Krüger, N. Krumnack, A. Kruse, M. C. Kruse, M. Kruskal, T. Kubota, H. Kucuk, S. Kuday, S. Kuehn, A. Kugel, F. Kuger, A. Kuhl, T. Kuhl, V. Kukhtin, R. Kukla, Y. Kulchitsky, S. Kuleshov, M. Kuna, T. Kunigo, A. Kupco, H. Kurashige, Y. A. Kurochkin, V. Kus, E. S. Kuwertz, M. Kuze, J. Kvita, T. Kwan, D. Kyriazopoulos, A. La Rosa, J. L. La Rosa Navarro, L. La Rotonda, C. Lacasta, F. Lacava, J. Lacey, H. Lacker, D. Lacour, V. R. Lacuesta, E. Ladygin, R. Lafaye, B. Laforge, T. Lagouri, S. Lai, L. Lambourne, S. Lammers, C. L. Lampen, W. Lampl, E. Lançon, U. Landgraf, M. P. J. Landon, V. S. Lang, J. C. Lange, A. J. Lankford, F. Lanni, K. Lantzsch, A. Lanza, S. Laplace, C. Lapoire, J. F. Laporte, T. Lari, F. Lasagni Manghi, M. Lassnig, P. Laurelli, W. Lavrijsen, A. T. Law, P. Laycock, T. Lazovich, O. Le Dortz, E. Le Guirriec, E. Le Menedeu, M. LeBlanc, T. LeCompte, F. Ledroit-Guillon, C. A. Lee, S. C. Lee, L. Lee, G. Lefebvre, M. Lefebvre, F. Legger, C. Leggett, A. Lehan, G. Lehmann Miotto, X. Lei, W. A. Leight, A. Leisos, A. G. Leister, M. A. L. Leite, R. Leitner, D. Lellouch, B. Lemmer, K. J. C. Leney, T. Lenz, B. Lenzi, R. Leone, S. Leone, C. Leonidopoulos, S. Leontsinis, C. Leroy, C. G. Lester, M. Levchenko, J. Levêque, D. Levin, L. J. Levinson, M. Levy, A. Lewis, A. M. Leyko, M. Leyton, B. Li, H. Li, H. L. Li, L. Li, L. Li, S. Li, X. Li, Y. Li, Z. Liang, H. Liao, B. Liberti, A. Liblong, P. Lichard, K. Lie, J. Liebal, W. Liebig, C. Limbach, A. Limosani, S. C. Lin, T. H. Lin, F. Linde, B. E. Lindquist, J. T. Linnemann, E. Lipeles, A. Lipniacka, M. Lisovyi, T. M. Liss, D. Lissauer, A. Lister, A. M. Litke, B. Liu, D. Liu, H. Liu, J. Liu, J. B. Liu, K. Liu, L. Liu, M. Liu, M. Liu, Y. Liu, M. Livan, A. Lleres, J. Llorente Merino, S. L. Lloyd, F. Lo Sterzo, E. Lobodzinska, P. Loch, W. S. Lockman, F. K. Loebinger, A. E. Loevschall-Jensen, K. M. Loew, A. Loginov, T. Lohse, K. Lohwasser, M. Lokajicek, B. A. Long, J. D. Long, R. E. Long, K. A. Looper, L. Lopes, D. Lopez Mateos, B. Lopez Paredes, I. Lopez Paz, J. Lorenz, N. Lorenzo Martinez, M. Losada, P. J. Lösel, X. Lou, A. Lounis, J. Love, P. A. Love, N. Lu, H. J. Lubatti, C. Luci, A. Lucotte, C. Luedtke, F. Luehring, W. Lukas, L. Luminari, O. Lundberg, B. Lund-Jensen, D. Lynn, R. Lysak, E. Lytken, H. Ma, L. L. Ma, G. Maccarrone, A. Macchiolo, C. M. Macdonald, B. Maček, J. Machado Miguens, D. Macina, D. Madaffari, R. Madar, H. J. Maddocks, W. F. Mader, A. Madsen, J. Maeda, S. Maeland, T. Maeno, A. Maevskiy, E. Magradze, K. Mahboubi, J. Mahlstedt, C. Maiani, C. Maidantchik, A. A. Maier, T. Maier, A. Maio, S. Majewski, Y. Makida, N. Makovec, B. Malaescu, Pa. Malecki, V. P. Maleev, F. Malek, U. Mallik, D. Malon, C. Malone, S. Maltezos, V. M. Malyshev, S. Malyukov, J. Mamuzic, G. Mancini, B. Mandelli, L. Mandelli, I. Mandić, R. Mandrysch, J. Maneira, A. Manfredini, L. Manhaes de Andrade Filho, J. Manjarres Ramos, A. Mann, A. Manousakis-Katsikakis, B. Mansoulie, R. Mantifel, M. Mantoani, L. Mapelli, L. March, G. Marchiori, M. Marcisovsky, C. P. Marino, M. Marjanovic, D. E. Marley, F. Marroquim, S. P. Marsden, Z. Marshall, L. F. Marti, S. Marti-Garcia, B. Martin, T. A. Martin, V. J. Martin, B. Martin dit Latour, M. Martinez, S. Martin-Haugh, V. S. Martoiu, A. C. Martyniuk, M. Marx, F. Marzano, A. Marzin, L. Masetti, T. Mashimo, R. Mashinistov, J. Masik, A. L. Maslennikov, I. Massa, L. Massa, P. Mastrandrea, A. Mastroberardino, T. Masubuchi, P. Mättig, J. Mattmann, J. Maurer, S. J. Maxfield, D. A. Maximov, R. Mazini, S. M. Mazza, L. Mazzaferro, G. Mc Goldrick, S. P. Mc Kee, A. McCarn, R. L. McCarthy, T. G. McCarthy, N. A. McCubbin, K. W. McFarlane, J. A. Mcfayden, G. Mchedlidze, S. J. McMahon, R. A. McPherson, M. Medinnis, S. Meehan, S. Mehlhase, A. Mehta, K. Meier, C. Meineck, B. Meirose, B. R. Mellado Garcia, F. Meloni, A. Mengarelli, S. Menke, E. Meoni, K. M. Mercurio, S. Mergelmeyer, P. Mermod, L. Merola, C. Meroni, F. S. Merritt, A. Messina, J. Metcalfe, A. S. Mete, C. Meyer, C. Meyer, J-P. Meyer, J. Meyer, H. Meyer Zu Theenhausen, R. P. Middleton, S. Miglioranzi, L. Mijović, G. Mikenberg, M. Mikestikova, M. Mikuž, M. Milesi, A. Milic, D. W. Miller, C. Mills, A. Milov, D. A. Milstead, A. A. Minaenko, Y. Minami, I. A. Minashvili, A. I. Mincer, B. Mindur, M. Mineev, Y. Ming, L. M. Mir, T. Mitani, J. Mitrevski, V. A. Mitsou, A. Miucci, P. S. Miyagawa, J. U. Mjörnmark, T. Moa, K. Mochizuki, S. Mohapatra, W. Mohr, S. Molander, R. Moles-Valls, R. Monden, K. Mönig, C. Monini, J. Monk, E. Monnier, J. Montejo Berlingen, F. Monticelli, S. Monzani, R. W. Moore, N. Morange, D. Moreno, M. Moreno Llácer, P. Morettini, D. Mori, M. Morii, M. Morinaga, V. Morisbak, S. Moritz, A. K. Morley, G. Mornacchi, J. D. Morris, S. S. Mortensen, A. Morton, L. Morvaj, M. Mosidze, J. Moss, K. Motohashi, R. Mount, E. Mountricha, S. V. Mouraviev, E. J. W. Moyse, S. Muanza, R. D. Mudd, F. Mueller, J. Mueller, R. S. P. Mueller, T. Mueller, D. Muenstermann, P. Mullen, G. A. Mullier, J. A. Murillo Quijada, W. J. Murray, H. Musheghyan, E. Musto, A. G. Myagkov, M. Myska, B. P. Nachman, O. Nackenhorst, J. Nadal, K. Nagai, R. Nagai, Y. Nagai, K. Nagano, A. Nagarkar, Y. Nagasaka, K. Nagata, M. Nagel, E. Nagy, A. M. Nairz, Y. Nakahama, K. Nakamura, T. Nakamura, I. Nakano, H. Namasivayam, R. F. Naranjo Garcia, R. Narayan, D. I. Narrias Villar, T. Naumann, G. Navarro, R. Nayyar, H. A. Neal, P. Yu. Nechaeva, T. J. Neep, P. D. Nef, A. Negri, M. Negrini, S. Nektarijevic, C. Nellist, A. Nelson, S. Nemecek, P. Nemethy, A. A. Nepomuceno, M. Nessi, M. S. Neubauer, M. Neumann, R. M. Neves, P. Nevski, P. R. Newman, D. H. Nguyen, R. B. Nickerson, R. Nicolaidou, B. Nicquevert, J. Nielsen, N. Nikiforou, A. Nikiforov, V. Nikolaenko, I. Nikolic-Audit, K. Nikolopoulos, J. K. Nilsen, P. Nilsson, Y. Ninomiya, A. Nisati, R. Nisius, T. Nobe, L. Nodulman, M. Nomachi, I. Nomidis, T. Nooney, S. Norberg, M. Nordberg, O. Novgorodova, S. Nowak, M. Nozaki, L. Nozka, K. Ntekas, G. Nunes Hanninger, T. Nunnemann, E. Nurse, F. Nuti, B. J. O’Brien, F. O’grady, D. C. O’Neil, V. O’Shea, F. G. Oakham, H. Oberlack, T. Obermann, J. Ocariz, A. Ochi, I. Ochoa, J. P. Ochoa-Ricoux, S. Oda, S. Odaka, H. Ogren, A. Oh, S. H. Oh, C. C. Ohm, H. Ohman, H. Oide, W. Okamura, H. Okawa, Y. Okumura, T. Okuyama, A. Olariu, S. A. Olivares Pino, D. Oliveira Damazio, E. Oliver Garcia, A. Olszewski, J. Olszowska, A. Onofre, K. Onogi, P. U. E. Onyisi, C. J. Oram, M. J. Oreglia, Y. Oren, D. Orestano, N. Orlando, C. Oropeza Barrera, R. S. Orr, B. Osculati, R. Ospanov, G. Otero y Garzon, H. Otono, M. Ouchrif, F. Ould-Saada, A. Ouraou, K. P. Oussoren, Q. Ouyang, A. Ovcharova, M. Owen, R. E. Owen, V. E. Ozcan, N. Ozturk, K. Pachal, A. Pacheco Pages, C. Padilla Aranda, M. Pagáčová, S. Pagan Griso, E. Paganis, F. Paige, P. Pais, K. Pajchel, G. Palacino, S. Palestini, M. Palka, D. Pallin, A. Palma, Y. B. Pan, E. St. Panagiotopoulou, C. E. Pandini, J. G. Panduro Vazquez, P. Pani, S. Panitkin, D. Pantea, L. Paolozzi, Th. D. Papadopoulou, K. Papageorgiou, A. Paramonov, D. Paredes Hernandez, M. A. Parker, K. A. Parker, F. Parodi, J. A. Parsons, U. Parzefall, E. Pasqualucci, S. Passaggio, F. Pastore, Fr. Pastore, G. Pásztor, S. Pataraia, N. D. Patel, J. R. Pater, T. Pauly, J. Pearce, B. Pearson, L. E. Pedersen, M. Pedersen, S. Pedraza Lopez, R. Pedro, S. V. Peleganchuk, D. Pelikan, O. Penc, C. Peng, H. Peng, B. Penning, J. Penwell, D. V. Perepelitsa, E. Perez Codina, M. T. Pérez García-Estañ, L. Perini, H. Pernegger, S. Perrella, R. Peschke, V. D. Peshekhonov, K. Peters, R. F. Y. Peters, B. A. Petersen, T. C. Petersen, E. Petit, A. Petridis, C. Petridou, P. Petroff, E. Petrolo, F. Petrucci, N. E. Pettersson, R. Pezoa, P. W. Phillips, G. Piacquadio, E. Pianori, A. Picazio, E. Piccaro, M. Piccinini, M. A. Pickering, R. Piegaia, D. T. Pignotti, J. E. Pilcher, A. D. Pilkington, A. W. J. Pin, J. Pina, M. Pinamonti, J. L. Pinfold, A. Pingel, S. Pires, H. Pirumov, M. Pitt, C. Pizio, L. Plazak, M.-A. Pleier, V. Pleskot, E. Plotnikova, P. Plucinski, D. Pluth, R. Poettgen, L. Poggioli, D. Pohl, G. Polesello, A. Poley, A. Policicchio, R. Polifka, A. Polini, C. S. Pollard, V. Polychronakos, K. Pommès, L. Pontecorvo, B. G. Pope, G. A. Popeneciu, D. S. Popovic, A. Poppleton, S. Pospisil, K. Potamianos, I. N. Potrap, C. J. Potter, C. T. Potter, G. Poulard, J. Poveda, V. Pozdnyakov, P. Pralavorio, A. Pranko, S. Prasad, S. Prell, D. Price, L. E. Price, M. Primavera, S. Prince, M. Proissl, K. Prokofiev, F. Prokoshin, E. Protopapadaki, S. Protopopescu, J. Proudfoot, M. Przybycien, E. Ptacek, D. Puddu, E. Pueschel, D. Puldon, M. Purohit, P. Puzo, J. Qian, G. Qin, Y. Qin, A. Quadt, D. R. Quarrie, W. B. Quayle, M. Queitsch-Maitland, D. Quilty, S. Raddum, V. Radeka, V. Radescu, S. K. Radhakrishnan, P. Radloff, P. Rados, F. Ragusa, G. Rahal, S. Rajagopalan, M. Rammensee, C. Rangel-Smith, F. Rauscher, S. Rave, T. Ravenscroft, M. Raymond, A. L. Read, N. P. Readioff, D. M. Rebuzzi, A. Redelbach, G. Redlinger, R. Reece, K. Reeves, L. Rehnisch, J. Reichert, H. Reisin, M. Relich, C. Rembser, H. Ren, A. Renaud, M. Rescigno, S. Resconi, O. L. Rezanova, P. Reznicek, R. Rezvani, R. Richter, S. Richter, E. Richter-Was, O. Ricken, M. Ridel, P. Rieck, C. J. Riegel, J. Rieger, O. Rifki, M. Rijssenbeek, A. Rimoldi, L. Rinaldi, B. Ristić, E. Ritsch, I. Riu, F. Rizatdinova, E. Rizvi, S. H. Robertson, A. Robichaud-Veronneau, D. Robinson, J. E. M. Robinson, A. Robson, C. Roda, S. Roe, O. Røhne, S. Rolli, A. Romaniouk, M. Romano, S. M. Romano Saez, E. Romero Adam, N. Rompotis, M. Ronzani, L. Roos, E. Ros, S. Rosati, K. Rosbach, P. Rose, P. L. Rosendahl, O. Rosenthal, V. Rossetti, E. Rossi, L. P. Rossi, J. H. N. Rosten, R. Rosten, M. Rotaru, I. Roth, J. Rothberg, D. Rousseau, C. R. Royon, A. Rozanov, Y. Rozen, X. Ruan, F. Rubbo, I. Rubinskiy, V. I. Rud, C. Rudolph, M. S. Rudolph, F. Rühr, A. Ruiz-Martinez, Z. Rurikova, N. A. Rusakovich, A. Ruschke, H. L. Russell, J. P. Rutherfoord, N. Ruthmann, Y. F. Ryabov, M. Rybar, G. Rybkin, N. C. Ryder, A. F. Saavedra, G. Sabato, S. Sacerdoti, A. Saddique, H. F-W. Sadrozinski, R. Sadykov, F. Safai Tehrani, P. Saha, M. Sahinsoy, M. Saimpert, T. Saito, H. Sakamoto, Y. Sakurai, G. Salamanna, A. Salamon, J. E. Salazar Loyola, M. Saleem, D. Salek, P. H. Sales De Bruin, D. Salihagic, A. Salnikov, J. Salt, D. Salvatore, F. Salvatore, A. Salvucci, A. Salzburger, D. Sammel, D. Sampsonidis, A. Sanchez, J. Sánchez, V. Sanchez Martinez, H. Sandaker, R. L. Sandbach, H. G. Sander, M. P. Sanders, M. Sandhoff, C. Sandoval, R. Sandstroem, D. P. C. Sankey, M. Sannino, A. Sansoni, C. Santoni, R. Santonico, H. Santos, I. Santoyo Castillo, K. Sapp, A. Sapronov, J. G. Saraiva, B. Sarrazin, O. Sasaki, Y. Sasaki, K. Sato, G. Sauvage, E. Sauvan, G. Savage, P. Savard, C. Sawyer, L. Sawyer, J. Saxon, C. Sbarra, A. Sbrizzi, T. Scanlon, D. A. Scannicchio, M. Scarcella, V. Scarfone, J. Schaarschmidt, P. Schacht, D. Schaefer, R. Schaefer, J. Schaeffer, S. Schaepe, S. Schaetzel, U. Schäfer, A. C. Schaffer, D. Schaile, R. D. Schamberger, V. Scharf, V. A. Schegelsky, D. Scheirich, M. Schernau, C. Schiavi, C. Schillo, M. Schioppa, S. Schlenker, K. Schmieden, C. Schmitt, S. Schmitt, S. Schmitt, B. Schneider, Y. J. Schnellbach, U. Schnoor, L. Schoeffel, A. Schoening, B. D. Schoenrock, E. Schopf, A. L. S. Schorlemmer, M. Schott, D. Schouten, J. Schovancova, S. Schramm, M. Schreyer, C. Schroeder, N. Schuh, M. J. Schultens, H.-C. Schultz-Coulon, H. Schulz, M. Schumacher, B. A. Schumm, Ph. Schune, C. Schwanenberger, A. Schwartzman, T. A. Schwarz, Ph. Schwegler, H. Schweiger, Ph. Schwemling, R. Schwienhorst, J. Schwindling, T. Schwindt, F. G. Sciacca, E. Scifo, G. Sciolla, F. Scuri, F. Scutti, J. Searcy, G. Sedov, E. Sedykh, P. Seema, S. C. Seidel, A. Seiden, F. Seifert, J. M. Seixas, G. Sekhniaidze, K. Sekhon, S. J. Sekula, D. M. Seliverstov, N. Semprini-Cesari, C. Serfon, L. Serin, L. Serkin, T. Serre, M. Sessa, R. Seuster, H. Severini, T. Sfiligoj, F. Sforza, A. Sfyrla, E. Shabalina, M. Shamim, L. Y. Shan, R. Shang, J. T. Shank, M. Shapiro, P. B. Shatalov, K. Shaw, S. M. Shaw, A. Shcherbakova, C. Y. Shehu, P. Sherwood, L. Shi, S. Shimizu, C. O. Shimmin, M. Shimojima, M. Shiyakova, A. Shmeleva, D. Shoaleh Saadi, M. J. Shochet, S. Shojaii, S. Shrestha, E. Shulga, M. A. Shupe, S. Shushkevich, P. Sicho, P. E. Sidebo, O. Sidiropoulou, D. Sidorov, A. Sidoti, F. Siegert, Dj. Sijacki, J. Silva, Y. Silver, S. B. Silverstein, V. Simak, O. Simard, Lj. Simic, S. Simion, E. Simioni, B. Simmons, D. Simon, P. Sinervo, N. B. Sinev, M. Sioli, G. Siragusa, A. N. Sisakyan, S. Yu. Sivoklokov, J. Sjölin, T. B. Sjursen, M. B. Skinner, H. P. Skottowe, P. Skubic, M. Slater, T. Slavicek, M. Slawinska, K. Sliwa, V. Smakhtin, B. H. Smart, L. Smestad, S. Yu. Smirnov, Y. Smirnov, L. N. Smirnova, O. Smirnova, M. N. K. Smith, R. W. Smith, M. Smizanska, K. Smolek, A. A. Snesarev, G. Snidero, S. Snyder, R. Sobie, F. Socher, A. Soffer, D. A. Soh, G. Sokhrannyi, C. A. Solans Sanchez, M. Solar, J. Solc, E. Yu. Soldatov, U. Soldevila, A. A. Solodkov, A. Soloshenko, O. V. Solovyanov, V. Solovyev, P. Sommer, H. Y. Song, N. Soni, A. Sood, A. Sopczak, B. Sopko, V. Sopko, V. Sorin, D. Sosa, M. Sosebee, C. L. Sotiropoulou, R. Soualah, A. M. Soukharev, D. South, B. C. Sowden, S. Spagnolo, M. Spalla, M. Spangenberg, F. Spanò, W. R. Spearman, D. Sperlich, F. Spettel, R. Spighi, G. Spigo, L. A. Spiller, M. Spousta, T. Spreitzer, R. D. St. Denis, A. Stabile, S. Staerz, J. Stahlman, R. Stamen, S. Stamm, E. Stanecka, R. W. Stanek, C. Stanescu, M. Stanescu-Bellu, M. M. Stanitzki, S. Stapnes, E. A. Starchenko, J. Stark, P. Staroba, P. Starovoitov, R. Staszewski, P. Steinberg, B. Stelzer, H. J. Stelzer, O. Stelzer-Chilton, H. Stenzel, G. A. Stewart, J. A. Stillings, M. C. Stockton, M. Stoebe, G. Stoicea, P. Stolte, S. Stonjek, A. R. Stradling, A. Straessner, M. E. Stramaglia, J. Strandberg, S. Strandberg, A. Strandlie, E. Strauss, M. Strauss, P. Strizenec, R. Ströhmer, D. M. Strom, R. Stroynowski, A. Strubig, S. A. Stucci, B. Stugu, N. A. Styles, D. Su, J. Su, R. Subramaniam, A. Succurro, S. Suchek, Y. Sugaya, M. Suk, V. V. Sulin, S. Sultansoy, T. Sumida, S. Sun, X. Sun, J. E. Sundermann, K. Suruliz, G. Susinno, M. R. Sutton, S. Suzuki, M. Svatos, M. Swiatlowski, I. Sykora, T. Sykora, D. Ta, C. Taccini, K. Tackmann, J. Taenzer, A. Taffard, R. Tafirout, N. Taiblum, H. Takai, R. Takashima, H. Takeda, T. Takeshita, Y. Takubo, M. Talby, A. A. Talyshev, J. Y. C. Tam, K. G. Tan, J. Tanaka, R. Tanaka, S. Tanaka, B. B. Tannenwald, N. Tannoury, S. Tapprogge, S. Tarem, F. Tarrade, G. F. Tartarelli, P. Tas, M. Tasevsky, T. Tashiro, E. Tassi, A. Tavares Delgado, Y. Tayalati, F. E. Taylor, G. N. Taylor, P. T. E. Taylor, W. Taylor, F. A. Teischinger, P. Teixeira-Dias, K. K. Temming, D. Temple, H. Ten Kate, P. K. Teng, J. J. Teoh, F. Tepel, S. Terada, K. Terashi, J. Terron, S. Terzo, M. Testa, R. J. Teuscher, T. Theveneaux-Pelzer, J. P. Thomas, J. Thomas-Wilsker, E. N. Thompson, P. D. Thompson, R. J. Thompson, A. S. Thompson, L. A. Thomsen, E. Thomson, M. Thomson, R. P. Thun, M. J. Tibbetts, R. E. Ticse Torres, V. O. Tikhomirov, Yu. A. Tikhonov, S. Timoshenko, E. Tiouchichine, P. Tipton, S. Tisserant, K. Todome, T. Todorov, S. Todorova-Nova, J. Tojo, S. Tokár, K. Tokushuku, K. Tollefson, E. Tolley, L. Tomlinson, M. Tomoto, L. Tompkins, K. Toms, E. Torrence, H. Torres, E. Torró Pastor, J. Toth, F. Touchard, D. R. Tovey, T. Trefzger, L. Tremblet, A. Tricoli, I. M. Trigger, S. Trincaz-Duvoid, M. F. Tripiana, W. Trischuk, B. Trocmé, C. Troncon, M. Trottier-McDonald, M. Trovatelli, P. True, L. Truong, M. Trzebinski, A. Trzupek, C. Tsarouchas, J. C-L. Tseng, P. V. Tsiareshka, D. Tsionou, G. Tsipolitis, N. Tsirintanis, S. Tsiskaridze, V. Tsiskaridze, E. G. Tskhadadze, I. I. Tsukerman, V. Tsulaia, S. Tsuno, D. Tsybychev, A. Tudorache, V. Tudorache, A. N. Tuna, S. A. Tupputi, S. Turchikhin, D. Turecek, R. Turra, A. J. Turvey, P. M. Tuts, A. Tykhonov, M. Tylmad, M. Tyndel, I. Ueda, R. Ueno, M. Ughetto, M. Ugland, F. Ukegawa, G. Unal, A. Undrus, G. Unel, F. C. Ungaro, Y. Unno, C. Unverdorben, J. Urban, P. Urquijo, P. Urrejola, G. Usai, A. Usanova, L. Vacavant, V. Vacek, B. Vachon, C. Valderanis, N. Valencic, S. Valentinetti, A. Valero, L. Valery, S. Valkar, E. Valladolid Gallego, S. Vallecorsa, J. A. Valls Ferrer, W. Van Den Wollenberg, P. C. Van Der Deijl, R. van der Geer, H. van der Graaf, N. van Eldik, P. van Gemmeren, J. Van Nieuwkoop, I. van Vulpen, M. C. van Woerden, M. Vanadia, W. Vandelli, R. Vanguri, A. Vaniachine, F. Vannucci, G. Vardanyan, R. Vari, E. W. Varnes, T. Varol, D. Varouchas, A. Vartapetian, K. E. Varvell, F. Vazeille, T. Vazquez Schroeder, J. Veatch, L. M. Veloce, F. Veloso, T. Velz, S. Veneziano, A. Ventura, D. Ventura, M. Venturi, N. Venturi, A. Venturini, V. Vercesi, M. Verducci, W. Verkerke, J. C. Vermeulen, A. Vest, M. C. Vetterli, O. Viazlo, I. Vichou, T. Vickey, O. E. Vickey Boeriu, G. H. A. Viehhauser, S. Viel, R. Vigne, M. Villa, M. Villaplana Perez, E. Vilucchi, M. G. Vincter, V. B. Vinogradov, I. Vivarelli, F. Vives Vaque, S. Vlachos, D. Vladoiu, M. Vlasak, M. Vogel, P. Vokac, G. Volpi, M. Volpi, H. von der Schmitt, H. von Radziewski, E. von Toerne, V. Vorobel, K. Vorobev, M. Vos, R. Voss, J. H. Vossebeld, N. Vranjes, M. Vranjes Milosavljevic, V. Vrba, M. Vreeswijk, R. Vuillermet, I. Vukotic, Z. Vykydal, P. Wagner, W. Wagner, H. Wahlberg, S. Wahrmund, J. Wakabayashi, J. Walder, R. Walker, W. Walkowiak, C. Wang, F. Wang, H. Wang, H. Wang, J. Wang, J. Wang, K. Wang, R. Wang, S. M. Wang, T. Wang, T. Wang, X. Wang, C. Wanotayaroj, A. Warburton, C. P. Ward, D. R. Wardrope, A. Washbrook, C. Wasicki, P. M. Watkins, A. T. Watson, I. J. Watson, M. F. Watson, G. Watts, S. Watts, B. M. Waugh, S. Webb, M. S. Weber, S. W. Weber, J. S. Webster, A. R. Weidberg, B. Weinert, J. Weingarten, C. Weiser, H. Weits, P. S. Wells, T. Wenaus, T. Wengler, S. Wenig, N. Wermes, M. Werner, P. Werner, M. Wessels, J. Wetter, K. Whalen, A. M. Wharton, A. White, M. J. White, R. White, S. White, D. Whiteson, F. J. Wickens, W. Wiedenmann, M. Wielers, P. Wienemann, C. Wiglesworth, L. A. M. Wiik-Fuchs, A. Wildauer, H. G. Wilkens, H. H. Williams, S. Williams, C. Willis, S. Willocq, A. Wilson, J. A. Wilson, I. Wingerter-Seez, F. Winklmeier, B. T. Winter, M. Wittgen, J. Wittkowski, S. J. Wollstadt, M. W. Wolter, H. Wolters, B. K. Wosiek, J. Wotschack, M. J. Woudstra, K. W. Wozniak, M. Wu, M. Wu, S. L. Wu, X. Wu, Y. Wu, T. R. Wyatt, B. M. Wynne, S. Xella, D. Xu, L. Xu, B. Yabsley, S. Yacoob, R. Yakabe, M. Yamada, D. Yamaguchi, Y. Yamaguchi, A. Yamamoto, S. Yamamoto, T. Yamanaka, K. Yamauchi, Y. Yamazaki, Z. Yan, H. Yang, H. Yang, Y. Yang, W-M. Yao, Y. Yasu, E. Yatsenko, K. H. Yau Wong, J. Ye, S. Ye, I. Yeletskikh, A. L. Yen, E. Yildirim, K. Yorita, R. Yoshida, K. Yoshihara, C. Young, C. J. S. Young, S. Youssef, D. R. Yu, J. Yu, J. M. Yu, J. Yu, L. Yuan, S. P. Y. Yuen, A. Yurkewicz, I. Yusuff, B. Zabinski, R. Zaidan, A. M. Zaitsev, J. Zalieckas, A. Zaman, S. Zambito, L. Zanello, D. Zanzi, C. Zeitnitz, M. Zeman, A. Zemla, Q. Zeng, K. Zengel, O. Zenin, T. Ženiš, D. Zerwas, D. Zhang, F. Zhang, H. Zhang, J. Zhang, L. Zhang, R. Zhang, X. Zhang, Z. Zhang, X. Zhao, Y. Zhao, Z. Zhao, A. Zhemchugov, J. Zhong, B. Zhou, C. Zhou, L. Zhou, L. Zhou, M. Zhou, N. Zhou, C. G. Zhu, H. Zhu, J. Zhu, Y. Zhu, X. Zhuang, K. Zhukov, A. Zibell, D. Zieminska, N. I. Zimine, C. Zimmermann, S. Zimmermann, Z. Zinonos, M. Zinser, M. Ziolkowski, L. Živković, G. Zobernig, A. Zoccoli, M. zur Nedden, G. Zurzolo, L. Zwalinski, ATLAS Collaboration

**Affiliations:** 1 Department of Physics, University of Adelaide, Adelaide, Australia; 2 Physics Department, SUNY Albany, Albany, NY USA; 3 Department of Physics, University of Alberta, Edmonton, AB Canada; 4 Department of Physics, Ankara University, Ankara, Turkey; 5 Istanbul Aydin University, Istanbul, Turkey; 6 Division of Physics, TOBB University of Economics and Technology, Ankara, Turkey; 7 LAPP, CNRS/IN2P3 and Université Savoie Mont Blanc, Annecy-le-Vieux, France; 8 High Energy Physics Division, Argonne National Laboratory, Argonne, IL USA; 9 Department of Physics, University of Arizona, Tucson, AZ USA; 10 Department of Physics, The University of Texas at Arlington, Arlington, TX USA; 11 Physics Department, University of Athens, Athens, Greece; 12 Physics Department, National Technical University of Athens, Zografou, Greece; 13 Institute of Physics, Azerbaijan Academy of Sciences, Baku, Azerbaijan; 14 Institut de Física d’Altes Energies (IFAE), The Barcelona Institute of Science and Technology, Barcelona, Spain; 15 Institute of Physics, University of Belgrade, Belgrade, Serbia; 16 Department for Physics and Technology, University of Bergen, Bergen, Norway; 17 Physics Division, Lawrence Berkeley National Laboratory and University of California, Berkeley, CA USA; 18 Department of Physics, Humboldt University, Berlin, Germany; 19 Albert Einstein Center for Fundamental Physics and Laboratory for High Energy Physics, University of Bern, Bern, Switzerland; 20 School of Physics and Astronomy, University of Birmingham, Birmingham, UK; 21 Department of Physics, Bogazici University, Istanbul, Turkey; 22 Department of Physics Engineering, Gaziantep University, Gaziantep, Turkey; 23 Department of Physics, Dogus University, Istanbul, Turkey; 24 Centro de Investigaciones, Universidad Antonio Narino, Bogota, Colombia; 25 INFN Sezione di Bologna, Bologna, Italy; 26 Dipartimento di Fisica e Astronomia, Università di Bologna, Bologna, Italy; 27 Physikalisches Institut, University of Bonn, Bonn, Germany; 28 Department of Physics, Boston University, Boston, MA USA; 29 Department of Physics, Brandeis University, Waltham, MA USA; 30 Universidade Federal do Rio De Janeiro COPPE/EE/IF, Rio de Janeiro, Brazil; 31 Electrical Circuits Department, Federal University of Juiz de Fora (UFJF), Juiz de Fora, Brazil; 32 Federal University of Sao Joao del Rei (UFSJ), São João del Rei, Brazil; 33 Instituto de Fisica, Universidade de Sao Paulo, São Paulo, Brazil; 34 Physics Department, Brookhaven National Laboratory, Upton, NY USA; 35 National Institute of Physics and Nuclear Engineering, Bucharest, Romania; 36 Physics Department, National Institute for Research and Development of Isotopic and Molecular Technologies, Cluj Napoca, Romania; 37 University Politehnica Bucharest, Bucharest, Romania; 38 West University in Timisoara, Timisoara, Romania; 39 Departamento de Física, Universidad de Buenos Aires, Buenos Aires, Argentina; 40 Cavendish Laboratory, University of Cambridge, Cambridge, UK; 41 Department of Physics, Carleton University, Ottawa, ON Canada; 42 CERN, Geneva, Switzerland; 43 Enrico Fermi Institute, University of Chicago, Chicago, IL USA; 44 Departamento de Física, Pontificia Universidad Católica de Chile, Santiago, Chile; 45 Departamento de Física, Universidad Técnica Federico Santa María, Valparaiso, Chile; 46 Institute of High Energy Physics, Chinese Academy of Sciences, Beijing, China; 47 Department of Modern Physics, University of Science and Technology of China, Hefei, Anhui China; 48 Department of Physics, Nanjing University, Nanjing, Jiangsu China; 49 School of Physics, Shandong University, Jinan, Shandong China; 50 Department of Physics and Astronomy, Shanghai Key Laboratory for Particle Physics and Cosmology, Shanghai Jiao Tong University (also affiliated with PKU-CHEP), Shanghai, China; 51 Physics Department, Tsinghua University, Beijing, 100084 China; 52 Laboratoire de Physique Corpusculaire, Clermont Université and Université Blaise Pascal and CNRS/IN2P3, Clermont-Ferrand, France; 53 Nevis Laboratory, Columbia University, Irvington, NY USA; 54 Niels Bohr Institute, University of Copenhagen, Kobenhavn, Denmark; 55 INFN Gruppo Collegato di Cosenza, Laboratori Nazionali di Frascati, Frascati, Italy; 56 Dipartimento di Fisica, Università della Calabria, Rende, Italy; 57 AGH University of Science and Technology, Faculty of Physics and Applied Computer Science, Kraków, Poland; 58 Marian Smoluchowski Institute of Physics, Jagiellonian University, Kraków, Poland; 59 Institute of Nuclear Physics, Polish Academy of Sciences, Kraków, Poland; 60 Physics Department, Southern Methodist University, Dallas, TX USA; 61 Physics Department, University of Texas at Dallas, Richardson, TX USA; 62 DESY, Hamburg and Zeuthen, Germany; 63 Institut für Experimentelle Physik IV, Technische Universität Dortmund, Dortmund, Germany; 64 Institut für Kern- und Teilchenphysik, Technische Universität Dresden, Dresden, Germany; 65 Department of Physics, Duke University, Durham, NC USA; 66 SUPA-School of Physics and Astronomy, University of Edinburgh, Edinburgh, UK; 67 INFN Laboratori Nazionali di Frascati, Frascati, Italy; 68 Fakultät für Mathematik und Physik, Albert-Ludwigs-Universität, Freiburg, Germany; 69 Section de Physique, Université de Genève, Geneva, Switzerland; 70 INFN Sezione di Genova, Geneva, Italy; 71 Dipartimento di Fisica, Università di Genova, Geneva, Italy; 72 E. Andronikashvili Institute of Physics, Iv. Javakhishvili Tbilisi State University, Tbilisi, Georgia; 73 High Energy Physics Institute, Tbilisi State University, Tbilisi, Georgia; 74 II Physikalisches Institut, Justus-Liebig-Universität Giessen, Giessen, Germany; 75 SUPA-School of Physics and Astronomy, University of Glasgow, Glasgow, UK; 76 II Physikalisches Institut, Georg-August-Universität, Göttingen, Germany; 77 Laboratoire de Physique Subatomique et de Cosmologie, Université Grenoble-Alpes, CNRS/IN2P3, Grenoble, France; 78 Department of Physics, Hampton University, Hampton, VA USA; 79 Laboratory for Particle Physics and Cosmology, Harvard University, Cambridge, MA USA; 80 Kirchhoff-Institut für Physik, Ruprecht-Karls-Universität Heidelberg, Heidelberg, Germany; 81 Physikalisches Institut, Ruprecht-Karls-Universität Heidelberg, Heidelberg, Germany; 82 ZITI Institut für technische Informatik, Ruprecht-Karls-Universität Heidelberg, Mannheim, Germany; 83 Faculty of Applied Information Science, Hiroshima Institute of Technology, Hiroshima, Japan; 84 Department of Physics, The Chinese University of Hong Kong, Shatin, NT Hong Kong; 85 Department of Physics, The University of Hong Kong, Pokfulam, Hong Kong; 86 Department of Physics, The Hong Kong University of Science and Technology, Clear Water Bay, Kowloon, Hong Kong China; 87 Department of Physics, Indiana University, Bloomington, IN USA; 88 Institut für Astro- und Teilchenphysik, Leopold-Franzens-Universität, Innsbruck, Austria; 89 University of Iowa, Iowa City, IA USA; 90 Department of Physics and Astronomy, Iowa State University, Ames, IA USA; 91 Department of Physics and Astronomy, University of California Irvine, Irvine, CA USA; 92 Joint Institute for Nuclear Research, JINR Dubna, Dubna, Russia; 93 KEK, High Energy Accelerator Research Organization, Tsukuba, Japan; 94 Graduate School of Science, Kobe University, Kobe, Japan; 95 Faculty of Science, Kyoto University, Kyoto, Japan; 96 Kyoto University of Education, Kyoto, Japan; 97 Department of Physics, Kyushu University, Fukuoka, Japan; 98 Instituto de Física La Plata, Universidad Nacional de La Plata and CONICET, La Plata, Argentina; 99 Physics Department, Lancaster University, Lancaster, UK; 100 INFN Sezione di Lecce, Lecce, Italy; 101 Dipartimento di Matematica e Fisica, Università del Salento, Lecce, Italy; 102 Oliver Lodge Laboratory, University of Liverpool, Liverpool, UK; 103 Department of Physics, Jožef Stefan Institute and University of Ljubljana, Ljubljana, Slovenia; 104 School of Physics and Astronomy, Queen Mary University of London, London, UK; 105 Department of Physics, Royal Holloway University of London, Surrey, UK; 106 Department of Physics and Astronomy, University College London, London, UK; 107 Louisiana Tech University, Ruston, LA USA; 108 Laboratoire de Physique Nucléaire et de Hautes Energies, UPMC and Université Paris-Diderot and CNRS/IN2P3, Paris, France; 109 Fysiska institutionen, Lunds universitet, Lund, Sweden; 110 Departamento de Fisica Teorica C-15, Universidad Autonoma de Madrid, Madrid, Spain; 111 Institut für Physik, Universität Mainz, Mainz, Germany; 112 School of Physics and Astronomy, University of Manchester, Manchester, UK; 113 CPPM, Aix-Marseille Université and CNRS/IN2P3, Marseille, France; 114 Department of Physics, University of Massachusetts, Amherst, MA USA; 115 Department of Physics, McGill University, Montreal, QC Canada; 116 School of Physics, University of Melbourne, Melbourne, VIC Australia; 117 Department of Physics, The University of Michigan, Ann Arbor, MI USA; 118 Department of Physics and Astronomy, Michigan State University, East Lansing, MI USA; 119 INFN Sezione di Milano, Milan, Italy; 120 Dipartimento di Fisica, Università di Milano, Milan, Italy; 121 B.I. Stepanov Institute of Physics, National Academy of Sciences of Belarus, Minsk, Republic of Belarus; 122 National Scientific and Educational Centre for Particle and High Energy Physics, Minsk, Republic of Belarus; 123 Department of Physics, Massachusetts Institute of Technology, Cambridge, MA USA; 124 Group of Particle Physics, University of Montreal, Montreal, QC Canada; 125 P.N. Lebedev Institute of the Russian Academy of Sciences, Moscow, Russia; 126 Institute for Theoretical and Experimental Physics (ITEP), Moscow, Russia; 127 National Research Nuclear University MEPhI, Moscow, Russia; 128 D.V. Skobeltsyn Institute of Nuclear Physics, M.V. Lomonosov Moscow State University, Moscow, Russia; 129 Fakultät für Physik, Ludwig-Maximilians-Universität München, München, Germany; 130 Max-Planck-Institut für Physik (Werner-Heisenberg-Institut), München, Germany; 131 Nagasaki Institute of Applied Science, Nagasaki, Japan; 132 Graduate School of Science and Kobayashi-Maskawa Institute, Nagoya University, Nagoya, Japan; 133 INFN Sezione di Napoli, Naples, Italy; 134 Dipartimento di Fisica, Università di Napoli, Naples, Italy; 135 Department of Physics and Astronomy, University of New Mexico, Albuquerque, NM USA; 136 Institute for Mathematics, Astrophysics and Particle Physics, Radboud University Nijmegen/Nikhef, Nijmegen, The Netherlands; 137 Nikhef National Institute for Subatomic Physics and University of Amsterdam, Amsterdam, The Netherlands; 138 Department of Physics, Northern Illinois University, DeKalb, IL USA; 139 Budker Institute of Nuclear Physics, SB RAS, Novosibirsk, Russia; 140 Department of Physics, New York University, New York, NY USA; 141 Ohio State University, Columbus, OH USA; 142 Faculty of Science, Okayama University, Okayama, Japan; 143 Homer L. Dodge Department of Physics and Astronomy, University of Oklahoma, Norman, OK USA; 144 Department of Physics, Oklahoma State University, Stillwater, OK USA; 145 Palacký University, RCPTM, Olomouc, Czech Republic; 146 Center for High Energy Physics, University of Oregon, Eugene, OR USA; 147 LAL, University of Paris-Sud, CNRS/IN2P3, Université Paris-Saclay, Orsay, France; 148 Graduate School of Science, Osaka University, Osaka, Japan; 149 Department of Physics, University of Oslo, Oslo, Norway; 150 Department of Physics, Oxford University, Oxford, UK; 151 INFN Sezione di Pavia, Pavia, Italy; 152 Dipartimento di Fisica, Università di Pavia, Pavia, Italy; 153 Department of Physics, University of Pennsylvania, Philadelphia, PA USA; 154 National Research Centre “Kurchatov Institute” B.P.Konstantinov Petersburg Nuclear Physics Institute, St. Petersburg, Russia; 155 INFN Sezione di Pisa, Pisa, Italy; 156 Dipartimento di Fisica E. Fermi, Università di Pisa, Pisa, Italy; 157 Department of Physics and Astronomy, University of Pittsburgh, Pittsburgh, PA USA; 158 Laboratório de Instrumentação e Física Experimental de Partículas, LIP, Lisbon, Portugal; 159 Faculdade de Ciências, Universidade de Lisboa, Lisbon, Portugal; 160 Department of Physics, University of Coimbra, Coimbra, Portugal; 161 Centro de Física Nuclear da Universidade de Lisboa, Lisbon, Portugal; 162 Departamento de Fisica, Universidade do Minho, Braga, Portugal; 163 Departamento de Fisica Teorica y del Cosmos and CAFPE, Universidad de Granada, Granada, Spain; 164 Departamento de Fisica and CEFITEC of Faculdade de Ciencias e Tecnologia, Universidade Nova de Lisboa, Caparica, Portugal; 165 Institute of Physics, Academy of Sciences of the Czech Republic, Praha, Czech Republic; 166 Czech Technical University in Prague, Praha, Czech Republic; 167 Faculty of Mathematics and Physics, Charles University in Prague, Praha, Czech Republic; 168 State Research Center Institute for High Energy Physics (Protvino), Moscow, NRC KI Russia; 169 Particle Physics Department, Rutherford Appleton Laboratory, Didcot, UK; 170 INFN Sezione di Roma, Rome, Italy; 171 Dipartimento di Fisica, Sapienza Università di Roma, Roma, Italy; 172 INFN Sezione di Roma Tor Vergata, Rome, Italy; 173 Dipartimento di Fisica, Università di Roma Tor Vergata, Roma, Italy; 174 INFN Sezione di Roma Tre, Roma, Italy; 175 Dipartimento di Matematica e Fisica, Università Roma Tre, Roma, Italy; 176 Faculté des Sciences Ain Chock, Réseau Universitaire de Physique des Hautes Energies, Université Hassan II, Casablanca, Morocco; 177 Centre National de l’Energie des Sciences Techniques Nucleaires, Rabat, Morocco; 178 Faculté des Sciences Semlalia, Université Cadi Ayyad, LPHEA-Marrakech, Marrakech, Morocco; 179 Faculté des Sciences, Université Mohamed Premier and LPTPM, Oujda, Morocco; 180 Faculté des Sciences, Université Mohammed V, Rabat, Morocco; 181 DSM/IRFU (Institut de Recherches sur les Lois Fondamentales de l’Univers), CEA Saclay (Commissariat à l’Energie Atomique et aux Energies Alternatives), Gif-sur-Yvette, France; 182 Santa Cruz Institute for Particle Physics, University of California Santa Cruz, Santa Cruz, CA USA; 183 Department of Physics, University of Washington, Seattle, WA USA; 184 Department of Physics and Astronomy, University of Sheffield, Sheffield, UK; 185 Department of Physics, Shinshu University, Nagano, Japan; 186 Fachbereich Physik, Universität Siegen, Siegen, Germany; 187 Department of Physics, Simon Fraser University, Burnaby, BC Canada; 188 SLAC National Accelerator Laboratory, Stanford, CA USA; 189 Faculty of Mathematics, Physics and Informatics, Comenius University, Bratislava, Slovak Republic; 190 Department of Subnuclear Physics, Institute of Experimental Physics of the Slovak Academy of Sciences, Kosice, Slovak Republic; 191 Department of Physics, University of Cape Town, Cape Town, South Africa; 192 Department of Physics, University of Johannesburg, Johannesburg, South Africa; 193 School of Physics, University of the Witwatersrand, Johannesburg, South Africa; 194 Department of Physics, Stockholm University, Stockholm, Sweden; 195 The Oskar Klein Centre, Stockholm, Sweden; 196 Physics Department, Royal Institute of Technology, Stockholm, Sweden; 197 Departments of Physics and Astronomy and Chemistry, Stony Brook University, Stony Brook, NY USA; 198 Department of Physics and Astronomy, University of Sussex, Brighton, UK; 199 School of Physics, University of Sydney, Sydney, Australia; 200 Institute of Physics, Academia Sinica, Taipei, Taiwan; 201 Department of Physics, Technion: Israel Institute of Technology, Haifa, Israel; 202 Raymond and Beverly Sackler School of Physics and Astronomy, Tel Aviv University, Tel Aviv, Israel; 203 Department of Physics, Aristotle University of Thessaloniki, Thessaloníki, Greece; 204 International Center for Elementary Particle Physics and Department of Physics, The University of Tokyo, Tokyo, Japan; 205 Graduate School of Science and Technology, Tokyo Metropolitan University, Tokyo, Japan; 206 Department of Physics, Tokyo Institute of Technology, Tokyo, Japan; 207 Department of Physics, University of Toronto, Toronto, ON Canada; 208 TRIUMF, Vancouver, BC Canada; 209 Department of Physics and Astronomy, York University, Toronto, ON Canada; 210 Faculty of Pure and Applied Sciences, and Center for Integrated Research in Fundamental Science and Engineering, University of Tsukuba, Tsukuba, Japan; 211 Department of Physics and Astronomy, Tufts University, Medford, MA USA; 212 INFN Gruppo Collegato di Udine, Sezione di Trieste, Udine, Italy; 213 ICTP, Trieste, Italy; 214 Dipartimento di Chimica Fisica e Ambiente, Università di Udine, Udine, Italy; 215 Department of Physics and Astronomy, University of Uppsala, Uppsala, Sweden; 216 Department of Physics, University of Illinois, Urbana, IL USA; 217 Instituto de Física Corpuscular (IFIC) and Departamento de Física Atómica, Molecular y Nuclear and Departamento de Ingeniería Electrónica and Instituto de Microelectrónica de Barcelona (IMB-CNM), University of Valencia and CSIC, Valencia, Spain; 218 Department of Physics, University of British Columbia, Vancouver, BC Canada; 219 Department of Physics and Astronomy, University of Victoria, Victoria, BC Canada; 220 Department of Physics, University of Warwick, Coventry, UK; 221 Waseda University, Tokyo, Japan; 222 Department of Particle Physics, The Weizmann Institute of Science, Rehovot, Israel; 223 Department of Physics, University of Wisconsin, Madison, WI USA; 224 Fakultät für Physik und Astronomie, Julius-Maximilians-Universität, Würzburg, Germany; 225 Fakultät für Mathematik und Naturwissenschaften, Fachgruppe Physik, Bergische Universität Wuppertal, Wuppertal, Germany; 226 Department of Physics, Yale University, New Haven, CT USA; 227 Yerevan Physics Institute, Yerevan, Armenia; 228 Centre de Calcul de l’Institut National de Physique Nucléaire et de Physique des Particules (IN2P3), Villeurbanne, France; 229 CERN, 1211 Geneva 23, Switzerland

## Abstract

The production rates of prompt and non-prompt $J/\psi$ and $\psi (2\mathrm {S})$ mesons in their dimuon decay modes are measured using 2.1 and 11.4 fb$^{-1}$ of data collected with the ATLAS experiment at the Large Hadron Collider, in proton–proton collisions at $\sqrt{s}=7$ and 8 respectively. Production cross-sections for prompt as well as non-prompt sources, ratios of $\psi (2\mathrm {S})$ to $J/\psi$ production, and the fractions of non-prompt production for $J/\psi$ and $\psi (2\mathrm {S})$ are measured as a function of meson transverse momentum and rapidity. The measurements are compared to theoretical predictions.

## Introduction

Measurements of heavy quark–antiquark bound states (quarkonia) production processes provide an insight into the nature of quantum chromodynamics (QCD) close to the boundary between the perturbative and non-perturbative regimes. More than forty years since the discovery of the $J/\psi$, the investigation of hidden heavy-flavour production in hadronic collisions still presents significant challenges to both theory and experiment.

In high-energy hadronic collisions, charmonium states can be produced either directly by short-lived QCD sources (“prompt” production), or by long-lived sources in the decay chains of beauty hadrons (“non-prompt” production). These can be separated experimentally using the distance between the proton–proton primary interaction and the decay vertex of the quarkonium state. While *Fixed-Order with Next-to-Leading-Log* (FONLL) calculations [1, 2], made within the framework of perturbative QCD, have been quite successful in describing non-prompt production of various quarkonium states, a satisfactory understanding of the prompt production mechanisms is still to be achieved.

The $\psi (2\mathrm {S})$ meson is the only vector charmonium state that is produced with no significant contributions from decays of higher-mass quarkonia, referred to as feed-down contributions. This provides a unique opportunity to study production mechanisms specific to $J^{PC}=1^{--}$ states [3–12]. Measurements of the production of $J^{++}$ states with $J=0, 1, 2$, [12–17], strongly coupled to the two-gluon channel, allow similar studies in the *CP*-even sector, complementary to the *CP*-odd vector sector. Production of $J/\psi$ mesons [3–7, 9–11, 13, 18–24] arises from a mixture of different sources, receiving contributions from the production of $1^{--}$ and $J^{++}$ states in comparable amounts.

Early attempts to describe the formation of charmonium [25–32] using leading-order perturbative QCD gave rise to a variety of models, none of which could explain the large production cross-sections measured at the Tevatron [3, 13, 21–23]. Within the colour-singlet model (CSM) [33], next-to-next-to-leading-order (NNLO) contributions to the hadronic production of S-wave quarkonia were calculated without introducing any new phenomenological parameters. However, technical difficulties have so far made it impossible to perform the full NNLO calculation, or to extend those calculations to the P-wave states. So it is not entirely surprising that the predictions of the model underestimate the experimental data for inclusive production of $J/\psi$ and $\Upsilon$ states, where the feed-down is significant, but offer a better description for $\psi (2\mathrm {S})$ production [18, 34].

Non-relativistic QCD (NRQCD) calculations that include colour-octet (CO) contributions [35] introduce a number of phenomenological parameters — long-distance matrix elements (LDMEs) — which are determined from fits to the experimental data, and can hence describe the cross-sections and differential spectra satisfactorily [36]. However, the attempts to describe the polarization of S-wave quarkonium states using this approach have not been so successful [37], prompting a suggestion [38] that a more coherent approach is needed for the treatment of polarization within the QCD-motivated models of quarkonium production.

Neither the CSM nor the NRQCD model gives a satisfactory explanation for the measurement of prompt $$J/\psi $$ production in association with the *W* [39] and *Z* [40] bosons: in both cases, the measured differential cross-section is larger than theoretical expectations [41–44]. It is therefore important to broaden the scope of comparisons between theory and experiment by providing a variety of experimental information about quarkonium production across a wider kinematic range. In this context, ATLAS has measured the inclusive differential cross-section of $$J/\psi $$ production, with 2.3 pb$$^{-1}$$ of integrated luminosity [18], at $$\sqrt{s} = 7$$ TeV using the data collected in 2010, as well as the differential cross-sections of the production of $$\chi _c$$ states (4.5 fb$$^{-1}$$) [14], and of the $$\psi (2\mathrm {S})$$ in its $$J/\psi \pi \pi $$ decay mode (2.1 fb$$^{-1})$$ [9], at $$\sqrt{s} = 7$$ TeV with data collected in 2011. The cross-section and polarization measurements from CDF [4], CMS [6, 7, 45, 46], LHCb [8, 10, 12, 46–48] and ALICE [5, 49, 50], cover a considerable variety of charmonium production characteristics in a wide kinematic range (transverse momentum $$p_{\text {T}}\le 100$$ GeV and rapidities $$|y|<5$$), thus providing a wealth of information for a new generation of theoretical models.

This paper presents a precise measurement of $J/\psi$ and $\psi (2\mathrm {S})$ production in the dimuon decay mode, both at $\sqrt{s} = 7$ TeV and at $\sqrt{s} = 8$ TeV. It is presented as a double-differential measurement in transverse momentum and rapidity of the quarkonium state, separated into prompt and non-prompt contributions, covering a range of transverse momenta $8 < p_{\text {T}}\le 110$ GeV and rapidities $|y|<2.0$. The ratios of $\psi (2\mathrm {S})$ to $J/\psi$ cross-sections for prompt and non-prompt processes are also reported, as well as the non-prompt fractions of $J/\psi$ and $\psi (2\mathrm {S})$.

## The ATLAS detector

The ATLAS experiment [51] is a general-purpose detector consisting of an inner tracker, a calorimeter and a muon spectrometer. The inner detector (ID) directly surrounds the interaction point; it consists of a silicon pixel detector, a semiconductor tracker and a transition radiation tracker, and is embedded in an axial 2 T magnetic field. The ID covers the pseudorapidity^1^ range $|\eta | =$ 2.5 and is enclosed by a calorimeter system containing electromagnetic and hadronic sections. The calorimeter is surrounded by a large muon spectrometer (MS) in a toroidal magnet system. The MS consists of monitored drift tubes and cathode strip chambers, designed to provide precise position measurements in the bending plane in the range $|\eta | <$ 2.7. Momentum measurements in the muon spectrometer are based on track segments formed in at least two of the three precision chamber planes.

The ATLAS trigger system [52] is separated into three levels: the hardware-based Level-1 trigger and the two-stage High Level Trigger (HLT), comprising the Level-2 trigger and Event Filter, which reduce the 20 MHz proton–proton collision rate to several-hundred Hz of events of interest for data recording to mass storage. At Level-1, the muon trigger searches for patterns of hits satisfying different transverse momentum thresholds with a coarse position resolution but a fast response time using resistive-plate chambers and thin-gap chambers in the ranges $|\eta | <$ 1.05 and $1.05 <|\eta | < 2.4$, respectively. Around these Level-1 hit patterns “Regions-of-Interest” (RoI) are defined that serve as seeds for the HLT muon reconstruction. The HLT uses dedicated algorithms to incorporate information from both the MS and the ID, achieving position and momentum resolution close to that provided by the offline muon reconstruction.

## Candidate selection

The analysis is based on data recorded at the LHC in 2011 and 2012 during proton–proton collisions at centre-of-mass energies of 7  and 8 TeV, respectively. This data sample corresponds to a total integrated luminosity of 2.1 and 11.4 fb$^{-1}$ for 7 and 8 TeV data, respectively.

Events were selected using a trigger requiring two oppositely charged muon candidates, each passing the requirement $p_{\text {T}}>4$ GeV. The muons are constrained to originate from a common vertex, which is fitted with the track parameter uncertainties taken into account. The fit is required to satisfy $\chi ^2 < 20$ for the one degree of freedom.

For 7 TeV data, the Level-1 trigger required only spatial coincidences in the MS [53]. For 8 TeV data, a 4 GeV muon $p_{\text {T}}$ threshold was also applied at Level-1, which reduced the trigger efficiency for low-$p_{\text {T}}$ muons.

The offline analysis requires events to have at least two muons, identified by the muon spectrometer and with matching tracks reconstructed in the ID [54]. Due to the ID acceptance, muon reconstruction is possible only for $|\eta | <$ 2.5. The selected muons are further restricted to $|\eta | <$ 2.3 to ensure high-quality tracking and triggering, and to reduce the contribution from misidentified muons. For the momenta of interest in this analysis (corresponding to muons with a transverse momentum of at most *O*(100) GeV), measurements of the muons are degraded by multiple scattering within the MS and so only the ID tracking information is considered. To ensure accurate ID measurements, each muon track must fulfil muon reconstruction and selection requirements [54]. The pairs of muon candidates satisfying these quality criteria are required to have opposite charges.

In order to allow an accurate correction for trigger inefficiencies, each reconstructed muon candidate is required to match a trigger-identified muon candidate within a cone of $\Delta R = \sqrt{(\Delta \eta )^2 + (\Delta \phi )^2}=0.01$. Dimuon candidates are obtained from muon pairs, constrained to originate from a common vertex using ID track parameters and uncertainties, with a requirement of $\chi ^2 < 20$ of the vertex fit for the one degree of freedom. All dimuon candidates with an invariant mass within $2.6 < m(\mu \mu ) < 4.0$ GeV and within the kinematic range $p_{\text {T}}(\mu \mu ) > 8$ GeV, $|y(\mu \mu )| < 2.0$ are retained for the analysis. If multiple candidates are found in an event (occurring in approximately $10^{-6}$ of selected events), all candidates are retained. The properties of the dimuon system, such as invariant mass $m(\mu \mu )$, transverse momentum $p_{\text {T}}(\mu \mu )$, and rapidity $|y(\mu \mu )|$ are determined from the result of the vertex fit.

## Methodology

The measurements are performed in intervals of dimuon $p_{\text {T}}$ and absolute value of the rapidity (|*y*|). The term “prompt” refers to the $J/\psi$ or $\psi (2\mathrm {S})$ states — hereafter called $\psi$ to refer to either — are produced from short-lived QCD decays, including feed-down from other charmonium states as long as they are also produced from short-lived sources. If the decay chain producing a $\psi$ state includes long-lived particles such as *b*-hadrons, then such $\psi$ mesons are labelled as “non-prompt”. Using a simultaneous fit to the invariant mass of the dimuon and its “pseudo-proper decay time” (described below), prompt and non-prompt signal and background contributions can be extracted from the data.

The probability for the decay of a particle as a function of proper decay time *t* follows an exponential distribution, $p(t) = 1/\tau _{B}\cdot e^{-t/\tau _{B}}$ where $\tau _{B}$ is the mean lifetime of the particle. For each decay, the proper decay time can be calculated as $t = L m/p$, where *L* is the distance between the particle production and decay vertices, *p* is the momentum of the particle, and *m* is its invariant mass. As the reconstruction of non-prompt $\psi$ mesons, such as *b*-hadrons, does not fully describe the properties of the parent, the transverse momentum of the dimuon system and the reconstructed dimuon invariant mass are used to construct the “pseudo-proper decay time”, $\tau = L_{xy} m(\mu \mu )/p_{\text {T}}(\mu \mu )$, where $L_{xy} \equiv \mathbf {L} \cdot \mathbf {p_{\text {T}}}(\mu \mu )/p_{\text {T}}(\mu \mu )$ is the signed projection of the distance of the dimuon decay vertex from the primary vertex, $\mathbf {L}$, onto its transverse momentum, $\mathbf {p_{\text {T}}}(\mu \mu )$. This is a good approximation of using the parent *b*-hadron information when the $\psi$ and parent momenta are closely aligned, which is the case for the values of $\psi$ transverse momenta considered here, and $\tau$ therefore can be used to distinguish statistically between the non-prompt and prompt processes (in which the latter are assumed to decay with vanishingly small lifetime). If the event contains multiple primary vertices [51], the primary vertex closest in *z* to the dimuon decay vertex is selected. The effect of selecting an incorrect vertex has been shown [55] to have a negligible impact on the extraction of prompt and non-prompt contributions. If any of the muons in the dimuon candidate contributes to the construction of the primary vertex, the corresponding tracks are removed and the vertex is refitted.

### Double differential cross-section determination

The double differential dimuon prompt and non-prompt production cross-sections times branching ratio are measured separately for $J/\psi$ and $\psi (2\mathrm {S})$ mesons according to the equations:

1 $$\begin{aligned} \frac{\mathrm {d}^2\sigma (pp \rightarrow \psi )}{\mathrm {d}p_{\text {T}}\mathrm {d}y} \times \mathcal {B} (\psi \rightarrow \mu ^+\mu ^- ) = \frac{N_{\psi }^{\mathrm {p}}}{\Delta p_{\text {T}}\Delta y \times \int \mathcal {L} \mathrm {d}t}, \end{aligned}$$

2 $$\begin{aligned}&\frac{\mathrm {d}^2\sigma (pp \rightarrow b\bar{b} \rightarrow \psi )}{\mathrm {d}p_{\text {T}}\mathrm {d}y} \times \mathcal {B} (\psi \rightarrow \mu ^+\mu ^- ) \nonumber \\&\quad = \frac{N_{\psi }^{\mathrm {np}}}{\Delta p_{\text {T}}\Delta y \times \int \mathcal {L} \mathrm {d}t}, \end{aligned}$$

where $\int \mathcal {L} dt$ is the integrated luminosity, $\Delta p_{\text {T}}$ and $\Delta y$ are the interval sizes in terms of dimuon transverse momentum and rapidity, respectively, and $N_{\psi }^{\mathrm {p(np)}}$ is the number of observed prompt (non-prompt) $\psi$ mesons in the slice under study, corrected for acceptance, trigger and reconstruction efficiencies. The intervals in $\Delta y$ combine the data from negative and positive rapidities.

The determination of the cross-sections proceeds in several steps. First, a weight is determined for each selected dimuon candidate equal to the inverse of the total efficiency for each candidate. The total weight, $w_\mathrm {tot}$, for each dimuon candidate includes three factors: the fraction of produced $\psi \rightarrow \mu ^+\mu ^-$ decays with both muons in the fiducial region $p_{\text {T}}(\mu ) > 4$ GeV and $|\eta (\mu )| <$ 2.3 (defined as acceptance, $\mathcal {A}$), the probability that a candidate within the acceptance satisfies the offline reconstruction selection ($\epsilon _\mathrm {reco}$), and the probability that a reconstructed event satisfies the trigger selection ($\epsilon _\mathrm {trig}$). The weight assigned to a given candidate when calculating the cross-sections is therefore given by:

$$\begin{aligned} w_{\mathrm {tot}}^{-1} = \mathcal {A}\cdot \epsilon _{\mathrm {reco}} \cdot \epsilon _{\mathrm {trig}}. \end{aligned}$$

After the weight determination, an unbinned maximum-likelihood fit is performed to these weighted events in each ($p_{\text {T}}(\mu \mu ), \ |y(\mu \mu )|$) interval using the dimuon invariant mass, $m(\mu \mu )$, and pseudo-proper decay time, $\tau (\mu \mu )$, observables. The fitted yields of $J/\psi \rightarrow \mu ^+\mu ^-$ and $\psi (2\mathrm {S})\rightarrow \mu ^+\mu ^-$ are determined separately for prompt and non-prompt processes. Finally, the differential cross-section times the $\psi \rightarrow \mu ^+\mu ^-$ branching fraction is calculated for each state by including the integrated luminosity and the $p_{\text {T}}$ and rapidity interval widths as shown in Eqs. (1) and (2).

### Non-prompt fraction

The non-prompt fraction $f_{b}^{\psi }$ is defined as the number of non-prompt $\psi$ (produced via the decay of a *b*-hadron) divided by the number of inclusively produced $\psi$ decaying to muon pairs after applying weighting corrections:

$$\begin{aligned} f_{b}^{\psi } \equiv \frac{pp \rightarrow b + X \rightarrow \psi + X'}{pp \xrightarrow {\mathrm {Inclusive}} \psi + X'} = \frac{N^{\mathrm {np}}_{\psi }}{N^{\mathrm {np}}_{\psi } + N^{\mathrm {p}}_{\psi }}, \end{aligned}$$

where this fraction is determined separately for $J/\psi$ and $\psi (2\mathrm {S})$. Determining the fraction from this ratio is advantageous since acceptance and efficiencies largely cancel and the systematic uncertainty is reduced.

### Ratio of $\psi (2\mathrm {S})$ to $J/\psi$ production

The ratio of $\psi (2\mathrm {S})$ to $J/\psi$ production, in their dimuon decay modes, is defined as:

$$\begin{aligned} R^{\mathrm {p(np)}} =\frac{N^{\mathrm {p(np)}} _{\psi (2\mathrm {S})}}{N^{\mathrm {p(np)}} _{J/\psi }}, \end{aligned}$$

where $N_{\psi }^{\mathrm {p(np)}}$ is the number of prompt (non-prompt) $J/\psi$ or $\psi (2\mathrm {S})$ mesons decaying into a muon pair in an interval of $p_{\text {T}}$ and *y*, corrected for selection efficiencies and acceptance.

For the ratio measurements, similarly to the non-prompt fraction, the acceptance and efficiency corrections largely cancel, thus allowing a more precise measurement. The theoretical uncertainties on such ratios are also smaller, as several dependencies, such as parton distribution functions and *b*-hadron production spectra, largely cancel in the ratio.

### Acceptance

The kinematic acceptance $\mathcal {A}$ for a $\psi \rightarrow \mu ^+\mu ^-$ decay with $p_{\text {T}}$ and *y* is given by the probability that both muons pass the fiducial selection ($p_{\text {T}}(\mu )>4$ GeV and $|\eta (\mu )|<2.3$). This is calculated using generator-level “accept-reject” simulations, based on the analytic formula described below. Detector-level corrections, such as bin migration effects due to detector resolution, are found to be small. They are applied to the results and are also considered as part of the systematic uncertainties.

The acceptance $\mathcal {A}$ depends on five independent variables (the two muon momenta are constrained by the $m(\mu \mu )$ mass condition), chosen as the $p_{\text {T}}$, |*y*| and azimuthal angle $\phi$ of the $\psi$ meson in the laboratory frame, and two angles characterizing the $\psi \rightarrow \mu ^+\mu ^-$ decay, $\theta ^{\star }$ and $\phi ^{\star }$, described in detail in Ref. [56]. The angle $\theta ^{\star }$ is the angle between the direction of the positive-muon momentum in the $\psi$ rest frame and the momentum of the $\psi$ in the laboratory frame, while $\phi ^{\star }$ is defined as the angle between the dimuon production and decay planes in the laboratory frame. The $\psi$ production plane is defined by the momentum of the $\psi$ in the laboratory frame and the positive *z*-axis direction. The distributions in $\theta ^{\star }$ and $\phi ^{\star }$ differ for various possible spin-alignment scenarios of the dimuon system.

The spin-alignment of the $\psi$ may vary depending on the production mechanism, which in turn affects the angular distribution of the dimuon decay. Predictions of various theoretical models are quite contradictory, while the recent experimental measurements [7] indicate that the angular dependence of $J/\psi$ and $\psi (2\mathrm {S})$ decays is consistent with being isotropic.

The coefficients $\lambda _{\theta }, \lambda _{\phi }$ and $\lambda _{\theta \phi }$ in

3 $$\begin{aligned} \frac{\mathrm {d}^2N}{\mathrm {d}\cos \theta ^{\star }\mathrm {d}\phi ^{\star }}&\propto 1 + \lambda _{\theta } \cos ^2\theta ^{\star } + \lambda _{\phi } \sin ^2\theta ^{\star }\cos 2\phi ^{\star }\nonumber \\&\quad + \lambda _{\theta \phi } \sin 2\theta ^{\star }\cos \phi ^{\star } \end{aligned}$$

are related to the spin-density matrix elements of the dimuon spin wave function.

Since the polarization of the $\psi$ state may affect acceptance, seven extreme cases that lead to the largest possible variations of acceptance within the phase space of this measurement are identified. These cases, described in Table 1, are used to define a range in which the results may vary under any physically allowed spin-alignment assumptions. The same technique has also been used in other measurements [9, 14, 34]. This analysis adopts the isotropic distribution in both $\cos \theta ^{\star }$ and $\phi ^{\star }$ as nominal, and the variation of the results for a number of extreme spin-alignment scenarios is studied and presented as sets of correction factors, detailed further in “Appendix”.

**Table 1:** Values of angular coefficients describing the considered spin-alignment scenarios

	Angular coefficients		
	$\lambda _{\theta }$	$\lambda _{\phi }$	$\lambda _{\theta \phi }$
Isotropic *(central value)*	0	0	0
Longitudinal	$-1$	0	0
Transverse positive	$+1$	$+1$	0
Transverse zero	$+1$	0	0
Transverse negative	$+1$	$-1$	0
Off-($\lambda _{\theta }$–$\lambda _{\phi }$)-plane positive	0	0	$+0.5$
Off-($\lambda _{\theta }$–$\lambda _{\phi }$)-plane negative	0	0	$-0.5$

For each of the two mass-points (corresponding to the $J/\psi$ and $\psi (2\mathrm {S})$ masses), two-dimensional maps are produced as a function of dimuon $p_{\text {T}}(\mu \mu )$ and $|y(\mu \mu )|$ for the set of spin-alignment hypotheses. Each point on the map is determined from a uniform sampling over $\phi ^{\star }$ and $\cos \theta ^{\star }$, accepting those trials that pass the fiducial selections. To account for various spin-alignment scenarios, all trials are weighted according to Eq. 3. Acceptance maps are defined within the range $8 < p_{\text {T}}(\mu \mu ) < 110$ GeVand $|y(\mu \mu )| < 2.0$, corresponding to the data considered in the analysis. The map is defined by 100 slices in $|y(\mu \mu )|$ and 4400 in $p_{\text {T}}(\mu \mu )$, using 200k trials for each point, resulting in sufficiently high precision that the statistical uncertainty can be neglected. Due to the contributions of background, and the detector resolution of the signal, the acceptance for each candidate is determined from a linear interpolation of the two maps, which are generated for the $J/\psi$ and $\psi (2\mathrm {S})$ known masses, as a function of the reconstructed mass $m(\mu \mu )$.

**Fig. 1:**
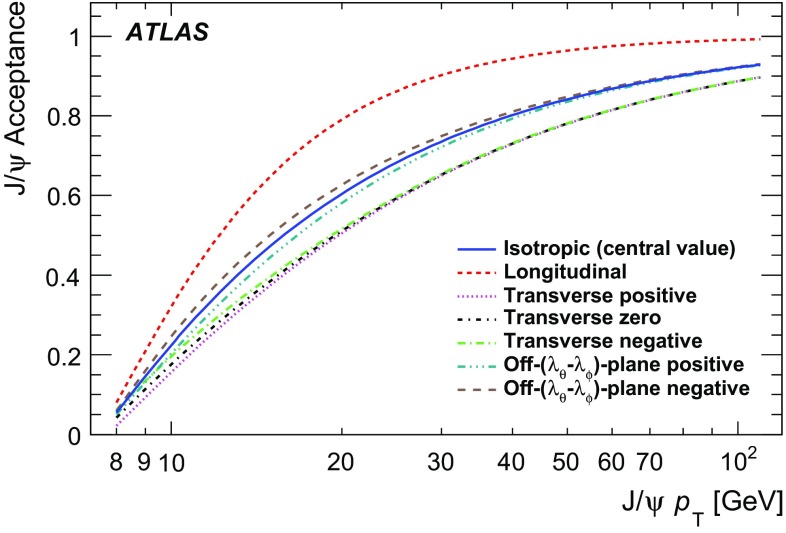
Projections of the acceptance as a function of $p_{\text {T}}$ for the $J/\psi$ meson for various spin-alignment hypotheses

Figure 1 shows the acceptance, projected in $p_{\text {T}}$ for all the spin-alignment hypotheses for the $J/\psi$ meson. The differences between the acceptance of the $\psi (2\mathrm {S})$ and $J/\psi$ meson, are independent of rapidity, except near $|y|\approx 2$ at low $p_{\text {T}}$. Similarly, the only dependence on $p_{\text {T}}$ is found below $p_{\text {T}}\approx 9$ GeV. The correction factors (as given in “Appendix”) vary most at low $p_{\text {T}}$, ranging from $-35\,\%$ under longitudinal, to $+100\,\%$ for transverse-positive scenarios. At high $p_{\text {T}}$, the range is between $-14\,\%$ for longitudinal, and $+9\,\%$ for transverse-positive scenarios. For the fraction and ratio measurements, the correction factor is determined from the appropriate ratio of the individual correction factors.

### Muon reconstruction and trigger efficiency determination

The technique for correcting the 7 TeV data for trigger and reconstruction inefficiencies is described in detail in Refs. [9, 34]. For the 8 TeV data, a similar technique is used, however different efficiency maps are required for each set of data, and the 8 TeV corrections are detailed briefly below.

The single-muon reconstruction efficiency is determined from a tag-and-probe study in dimuon decays [40]. The efficiency map is calculated as a function of $p_{\text {T}}(\mu )$ and $q\times \eta (\mu )$, where $q=\pm 1$ is the electrical charge of the muon, expressed in units of *e*.

The trigger efficiency correction consists of two components. The first part represents the trigger efficiency for a single muon in intervals of $p_{\text {T}}(\mu )$ and $q\times \eta (\mu )$. For the dimuon system there is a second correction to account for reductions in efficiency due to closely spaced muons firing only a single RoI, vertex-quality cuts, and opposite-sign requirements. This correction is performed in three rapidity intervals: 0–1.0, 1.0–1.2 and 1.2–2.3. The correction is a function of $\Delta R(\mu \mu )$ in the first two rapidity intervals and a function of $\Delta R(\mu \mu )$ and $|y(\mu \mu )|$ in the last interval.

**Fig. 2:**
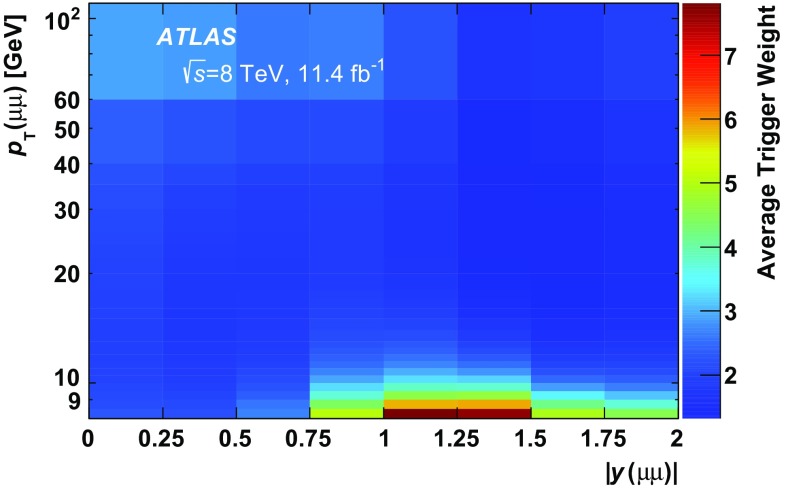
Average dimuon trigger-weight in the intervals of $p_{\text {T}}(\mu \mu )$ and $|y(\mu \mu )|$ studied in this set of measurements

The combination of the two components (single-muon efficiency map and dimuon corrections) is illustrated in Fig. 2 by plotting the average trigger-weight correction for the events in this analysis in terms of $p_{\text {T}}(\mu \mu )$ and $|y(\mu \mu )|$. The increased weight at low $p_{\text {T}}$ and $|y|\approx 1.25$ is caused by the geometrical acceptance of the muon trigger system and the turn-on threshold behaviour of the muon trigger. At high $p_{\text {T}}$ the weight is increased due to the reduced opening angle between the two muons.

### Fitting technique

To extract the corrected yields of prompt and non-prompt $J/\psi$ and $\psi (2\mathrm {S})$ mesons, two-dimensional weighted unbinned maximum-likelihood fits are performed on the dimuon invariant mass, $m(\mu \mu )$, and pseudo-proper decay time, $\tau (\mu \mu )$, in intervals of $p_{\text {T}}(\mu \mu )$ and $|y(\mu \mu )|$. Each interval is fitted independently from all the others. In $m(\mu \mu )$, signal processes of $\psi$ meson decays are statistically distinguished as narrow peaks convolved with the detector resolution, at their respective mass positions, on top of background continuum. In $\tau (\mu \mu )$, decays originating with zero pseudo-proper decay time and those following an exponential decay distribution (both convolved with a detector resolution function) statistically distinguish prompt and non-prompt signal processes, respectively. Various sources of background processes include Drell-Yan processes, mis-reconstructed muon pairs from prompt and non-prompt sources, and semileptonic decays from separate *b*-hadrons.

The probability density function (PDF) for each fit is defined as a normalized sum, where each term represents a specific signal or background contribution, with a physically motivated mass and $\tau$ dependence. The PDF can be written in a compact form as

4 $$\begin{aligned} \mathrm {PDF}(m,\tau ) = \sum _{i=1}^{7} \kappa _i f_i(m) \ \cdot h_i(\tau ) \otimes R(\tau ), \end{aligned}$$

where $\kappa _i$ represents the relative normalization of the $i^\mathrm {th}$ term of the seven considered signal and background contributions (such that $\sum _i \kappa _i = 1$), $f_i(m)$ is the mass-dependent term, and $\otimes$ represents the convolution of the $\tau$-dependent function $h_i(\tau )$ with the $\tau$ resolution term, $R(\tau )$. The latter is modelled by a double Gaussian distribution with both means fixed to zero and widths determined from the fit.

Table 2 lists the contributions to the overall PDF with the corresponding $f_i$ and $h_i$ functions. Here $G_1$ and $G_2$ are Gaussian functions, $B_1$ and $B_2$ are Crystal Ball^2^ distributions [57], while F is a uniform distribution and $C_1$ a first-order Chebyshev polynomial. The exponential functions $E_1$, $E_2$, $E_3$, $E_4$ and $E_5$ have different decay constants, where $E_5(|\tau |)$ is a double-sided exponential with the same decay constant on either side of $\tau = 0$. The parameter $\omega$ represents the fractional contribution of the *B* and *G* mass signal functions, while the Dirac delta function, $\delta (\tau )$, is used to represent the pseudo-proper decay time distribution of the prompt candidates.

**Table 2:** Description of the fit model PDF in Eq. 4. Components of the probability density function used to extract the prompt (P) and non-prompt (NP) contributions for $J/\psi$ and $\psi (2\mathrm {S})$ signal and the P, NP, and incoherent or mis-reconstructed background (Bkg) contributions

*i*	Type	Source	$f_i(m)$	$h_i(\tau )$
1	$J/\psi$	P	$\omega B_1(m) + (1-\omega ) G_1(m)$	$\delta (\tau )$
2	$J/\psi$	NP	$\omega B_1(m) + (1-\omega ) G_1(m)$	$E_1(\tau )$
3	$\psi (2\mathrm {S})$	P	$\omega B_2(m) + (1-\omega ) G_2(m)$	$\delta (\tau )$
4	$\psi (2\mathrm {S})$	NP	$\omega B_2(m) + (1-\omega ) G_2(m)$	$E_2(\tau )$
5	Bkg	P	*F*	$\delta (\tau )$
6	Bkg	NP	$$ C_1(m)$$	$$E_3 (\tau )$$
7	Bkg	NP	$$ E_4(m)$$	$$E_5 (|\tau |)$$

**Fig. 3:**
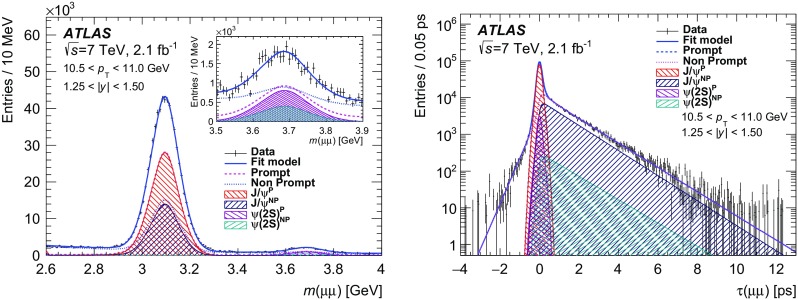
Projections of the fit result over the mass (*left*) and pseudo-proper decay time (*right*) distributions for data collected at 7 TeV for one typical interval. The data are shown with *error bars* in *black*, superimposed with the individual components of the fit result projections, where the total prompt and non-prompt components are represented by the *dashed* and *dotted lines*, respectively, and the *shaded areas* show the signal $\psi$ prompt and non-prompt contributions

In order to make the fitting procedure more robust and to reduce the number of free parameters, a number of component terms share common parameters, which led to 22 free parameters per interval. In detail, the signal mass models are described by the sum of a Crystal Ball shape (*B*) and a Gaussian shape (*G*). For each of $J/\psi$ and $\psi (2\mathrm {S})$, the *B* and *G* share a common mean, and freely determined widths, with the ratio of the *B* and *G* widths common to $J/\psi$ and $\psi (2\mathrm {S})$. The *B* parameters $\alpha$, and *n*, describing the transition point of the low-edge from a Gaussian to a power-law shape, and the shape of the tail, respectively, are fixed, and variations are considered as part of the fit model systematic uncertainties. The width of *G* for $\psi (2\mathrm {S})$ is set to the width for $J/\psi$ multiplied by a free parameter scaling term. The relative fraction of *B* and *G* is left floating, but common to $J/\psi$ and $\psi (2\mathrm {S})$.

The non-prompt signal decay shapes ($E_1$,$E_2$) are described by an exponential function (for positive $\tau$ only) convolved with a double Gaussian function, $R(\tau )$ describing the pseudo-proper decay time resolution for the non-prompt component, and the same Gaussian response functions to describe the prompt contributions. Each Gaussian resolution component has its mean fixed at $\tau$ = 0 and a free width. The decay constants of the $J/\psi$ and $\psi (2\mathrm {S})$ are separate free parameters in the fit.

The background contributions are described by a prompt and non-prompt component, as well as a double-sided exponential function convolved with a double Gaussian function describing mis-reconstructed or non-coherent muon pairs. The same resolution function as in signal is used to describe the background. For the non-resonant mass parameterizations, the non-prompt contribution is modelled by a first-order Chebyshev polynomial. The prompt mass contribution follows a flat distribution and the double-sided background uses an exponential function. Variations of this fit model are considered as systematic uncertainties.

The following quantities are extracted directly from the fit in each interval: the fraction of events that are signal (prompt or non-prompt $J/\psi$ or $\psi (2\mathrm {S})$); the fraction of signal events that are prompt; the fraction of prompt signal that is $\psi (2\mathrm {S})$; and the fraction of non-prompt signal that is $\psi (2\mathrm {S})$. From these parameters, and the weighted sum of events, all measured values are calculated.

**Fig. 4:**
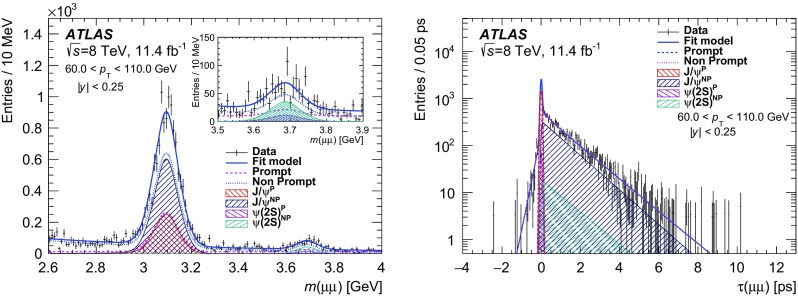
Projections of the fit result over the mass (*left*) and pseudo-proper decay time (*right*) distributions for data collected at 8 TeV for one high-$p_{\text {T}}$ interval. The data are shown with *error bars* in *black*, superimposed with the individual components of the fit result projections, where the total prompt and non-prompt components are represented by the *dashed* and *dotted lines*, respectively, and the *shaded areas* show the signal $\psi$ prompt and non-prompt contributions

For 7 TeV data, 168 fits are performed across the range of $8<p_{\text {T}}<100$ GeV ($8<p_{\text {T}}<60$ GeV) for $J/\psi$ ($\psi (2\mathrm {S})$) and $0<|y|<2$. For 8 TeV data, 172 fits are performed across the range of $8<p_{\text {T}}<110$ GeV and $0<|y|<2$, excluding the area where $p_{\text {T}}$ is less than 10 GeV and simultaneously |*y*| is greater than 0.75. This region is excluded due to a steeply changing low trigger efficiency causing large systematic uncertainties in the measured cross-section.

Figure 3 shows the fit results for one of the intervals considered in the analysis, projected onto the invariant mass and pseudo-proper decay time distributions, for 7 TeV data, weighted according to the acceptance and efficiency corrections. The fit projections are shown for the total prompt and total non-prompt contributions (shown as curves), and also for the individual contributions of the $J/\psi$ and $\psi (2\mathrm {S})$ prompt and non-prompt signal yields (shown as hashed areas of various types).

In Fig. 4 the fit results are shown for one high-$p_{\text {T}}$ interval of 8 TeV data.

### Bin migration corrections

To account for bin migration effects due to the detector resolution, which results in decays of $\psi$ in one bin, being identified and accounted for in another, the numbers of acceptance- and efficiency-corrected dimuon decays extracted from the fits in each interval of $p_{\text {T}}(\mu \mu )$ and rapidity are corrected for the differences between the true and reconstructed values of the dimuon $p_{\text {T}}$. These corrections are derived from data by comparing analytic functions that are fitted to the $p_{\text {T}}(\mu \mu )$ spectra of dimuon events with and without convolution by the experimental resolution in $p_{\text {T}}(\mu \mu )$ (as determined from the fitted mass resolution and measured muon angular resolutions), as described in Ref. [34].

**Table 3:** Summary of the minimum and maximum contributions along with the median value of the systematic uncertainties as percentages for the prompt and non-prompt $\psi$ cross-section results. Values are quoted for 7 and 8 TeV data

	7 TeV (%)			8 TeV (%)		
Source of systematic uncertainty	Min	Median	Max	Min	Median	Max
Luminosity	1.8	1.8	1.8	2.8	2.8	2.8
Muon reconstruction efficiency	0.7	1.2	4.7	0.3	0.7	6.0
Muon trigger efficiency	3.2	4.7	35.9	2.9	7.0	23.4
Inner detector tracking efficiency	1.0	1.0	1.0	1.0	1.0	1.0
Fit model parameterizations	0.5	2.2	22.6	0.26	1.07	24.9
Bin migrations	0.01	0.1	1.4	0.01	0.3	1.5
Total	4.2	6.5	36.3	4.4	8.1	27.9

The correction factors applied to the fitted yields deviate from unity by no more than $1.5\,\%$, and for the majority of slices are smaller than $1\,\%$. The ratio measurement and non-prompt fractions are corrected by the corresponding ratios of bin migration correction factors. Using a similar technique, bin migration corrections as a function of |*y*| are found to differ from unity by negligible amounts.

## Systematic uncertainties

The sources of systematic uncertainties that are applied to the $\psi$ double differential cross-section measurements are from uncertainties in: the luminosity determination; muon and trigger efficiency corrections; inner detector tracking efficiencies; the fit model parametrization; and due to bin migration corrections. For the non-prompt fraction and ratio measurements the systematic uncertainties are assessed in the same manner as for the uncertainties on the cross-section, except that in these ratios some systematic uncertainties, such as the luminosity uncertainty, cancel out. The sources of systematic uncertainty evaluated for the prompt and non-prompt $\psi$ cross-section measurements, along with the minimum, maximum and median values, are listed in Table 3. The largest contributions, which originate from the trigger and fit model uncertainties, are typically for the high $p_{\text {T}}$ intervals and are due to the limited statistics of the efficiency maps (for the trigger), and the data sample (for the fit model).

**Fig. 5:**
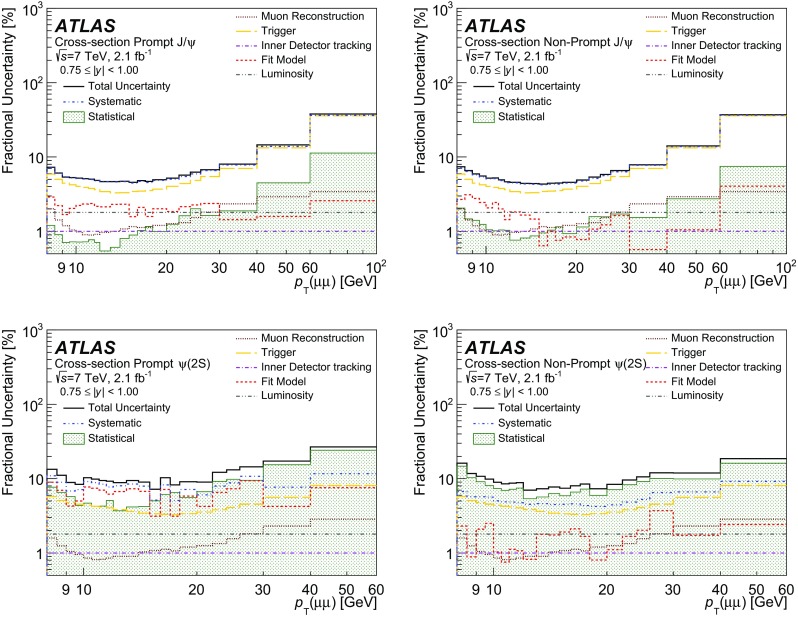
Statistical and systematic contributions to the fractional uncertainty on the prompt (*left column*) and non-prompt (*right column*) $J/\psi$ (*top row*) and $\psi (2\mathrm {S})$ (*bottom row*) cross-sections for 7 TeV, shown for the region $0.75<|y|<1.00$

**Fig. 6:**
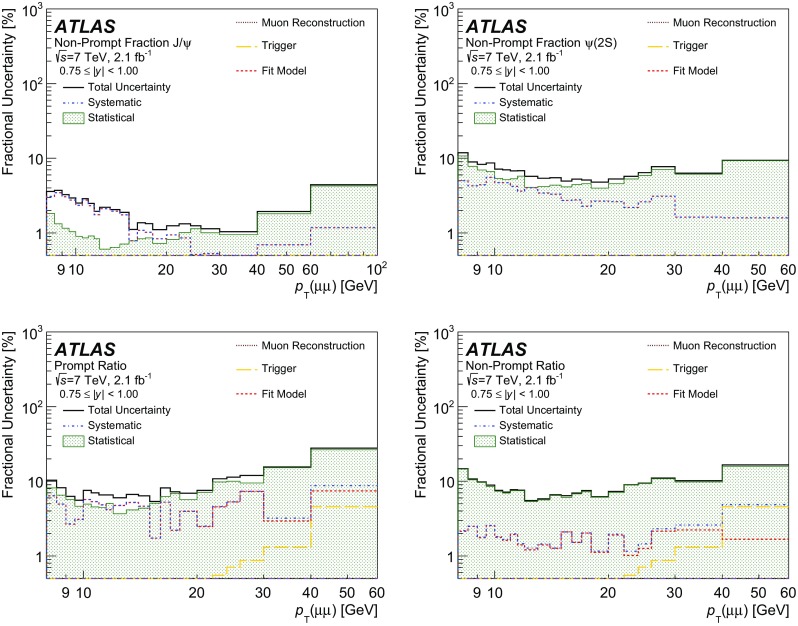
Breakdown of the contributions to the fractional uncertainty on the non-prompt fractions for $J/\psi$ (*top left*) and $\psi (2\mathrm {S})$ (*top right*), and the prompt (*bottom left*) and non-prompt (*bottom right*) ratios for 7 TeV, shown for the region $0.75<|y|<1.00$

Figures 5 and 6 show, for a representative interval, the impact of the considered uncertainties on the production cross-section, as well as the non-prompt fraction and ratios for 7 TeV data. The impact is very similar at 8 TeV.

*Luminosity*.   The uncertainty on the integrated luminosity is $1.8\,\%$ ($2.8\,\%$) for the 7 TeV (8 TeV) data-taking period. The methodology used to determine these uncertainties is described in Ref. [58]. The luminosity uncertainty is only applied to the $J/\psi$ and $\psi (2\mathrm {S})$ cross-section results.

*Muon reconstruction and trigger efficiencies*. To determine the systematic uncertainty on the muon reconstruction and trigger efficiency maps, each of the maps is reproduced in 100 pseudo-experiments. The dominant uncertainty in each bin is statistical and hence any bin-to-bin correlations are neglected. For each pseudo-experiment a new map is created by varying independently each bin content according to a Gaussian distribution about its estimated value, determined from the original map. In each pseudo-experiment, the total weight is recalculated for each dimuon $p_{\text {T}}$ and |*y*| interval of the analysis. The RMS of the total weight pseudo-experiment distributions for each efficiency type is used as the systematic uncertainty, where any correlation effects between the muon and trigger efficiencies can be neglected.

The ID tracking efficiency is in excess of $99.5\,\%$ [34], and an uncertainty of 1 % is applied to account for the ID dimuon reconstruction inefficiency (0.5 % per muon, added coherently). This uncertainty is applied to the differential cross-sections and is assumed to cancel in the fraction of non-prompt to inclusive production for $J/\psi$ and $\psi (2\mathrm {S})$ and in the ratios of $\psi (2\mathrm {S})$ to $J/\psi$ production.

For the trigger efficiency $\epsilon _\mathrm {trig}$, in addition to the trigger efficiency map, there is an additional correction term that accounts for inefficiencies due to correlations between the two trigger muons, such as the dimuon opening angle. This correction is varied by its uncertainty, and the shift in the resultant total weight relative to its central value is added in quadrature to the uncertainty from the map. The choice of triggers is known [59] to introduce a small lifetime-dependent efficiency loss but it is determined to have a negligible effect on the prompt and non-prompt yields and no correction is applied in this analysis. Similarly, the muon reconstruction efficiency corrections of prompt and non-prompt signals are found to be consistent within the statistical uncertainties of the efficiency measurements, and no additional uncertainty is applied.

*Fit model uncertainty*

The uncertainty due to the fit procedure is determined by varying one component at a time in the fit model described in Sect. 4.6, creating a set of new fit models. For each new fit model, all measured quantities are recalculated, and in each $p_{\text {T}}$ and |*y*| interval the spread of variations around the central fit model is used as its systematic uncertainty. The variations of the fit model also account for possible uncertainties due to final-state radiation. The following variations to the central model fit are evaluated:

- *Signal mass model*.  Using double Gaussian models in place of the Crystal Ball plus Gaussian model; variation of the $\alpha$ and *n* parameters of the *B* model, which are originally fixed;
- *Signal pseudo-proper decay time model*.  A double exponential function is used to describe the pseudo-proper decay time distribution for the $\psi$ non-prompt signal;
- *Background mass models*.  Variations of the mass model using exponentials functions, or quadratic Chebyshev polynomials to describe the components of prompt, non-prompt and double-sided background terms;
- *Background pseudo-proper decay time model*.  A single exponential function was considered for the non-prompt component;
- *Pseudo-proper decay time resolution model*.  Using a single Gaussian function in place of the double Gaussian function to model the lifetime resolution (also prompt lifetime model); and variation of the mixing terms for the two Gaussian components of this term.

Of the variations considered, it is typically the parametrizations of the signal mass model and pseudo-proper decay time resolution model that dominate the contribution to the fit model uncertainty.

*Bin migrations*. As the corrections to the results due to bin migration effects are factors close to unity in all regions, the difference between the correction factor and unity is applied as the uncertainty.

The variation of the acceptance corrections with spin-alignment is treated separately, and scaling factors supplied in “Appendix”.

## Results

The $J/\psi$ and $\psi (2\mathrm {S})$ non-prompt and prompt production cross-sections are presented, corrected for acceptance and detector efficiencies while assuming isotropic decay, as described in Sect. 4.1. Also presented are the ratios of non-prompt production relative to the inclusive production for $J/\psi$ and $\psi (2\mathrm {S})$ mesons separately, described in Sect. 4.2, and the ratio of $\psi (2\mathrm {S})$ to $J/\psi$ production for prompt and non-prompt components separately, described in Sect. 4.3. Correction factors for various spin-alignment hypotheses for both 7 and 8 TeV data can be found in Tables 4, 5, 6, 7, 8, 9, 10, 11, 12, 13, 14 and 15 (in “Appendix”) and Tables 16, 17, 18, 19, 20, 21, 22, 23, 24, 25, 26 and 27 (in “Appendix”) respectively, in terms of $p_{\text {T}}$ and rapidity intervals.

*Production cross-sections*

**Fig. 7:**
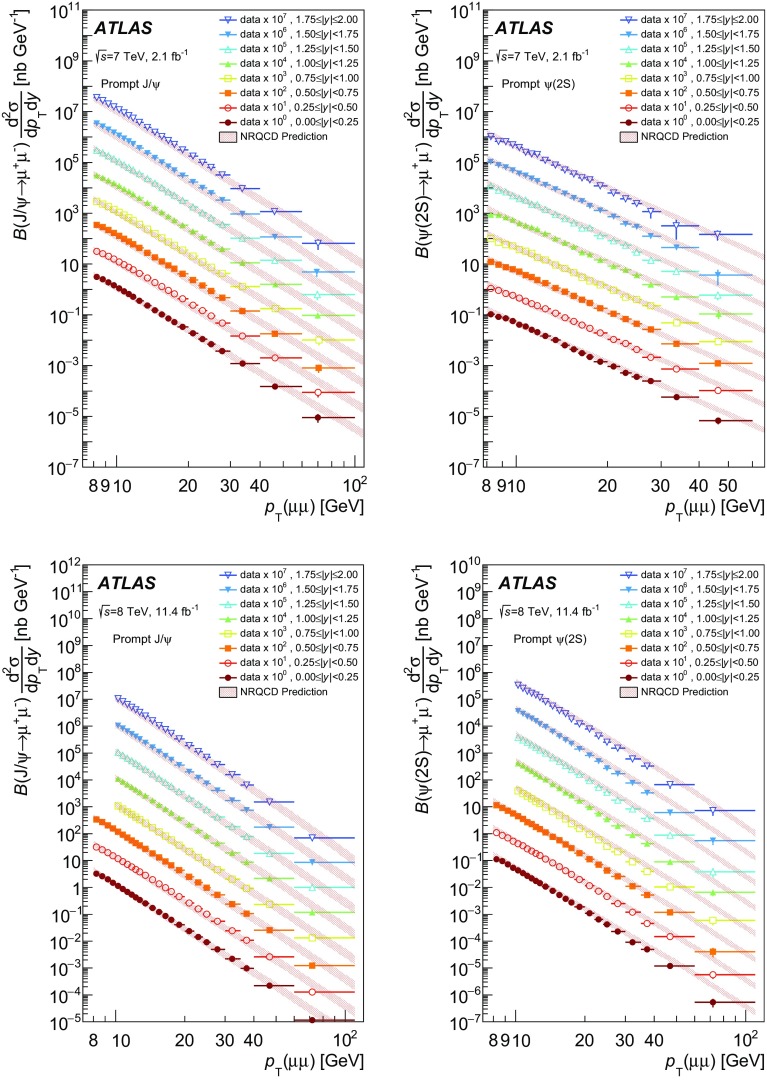
The differential prompt cross-section times dimuon branching fraction of $J/\psi$ (*left*) and $\psi (2\mathrm {S})$ (*right*) as a function of $p_{\text {T}}(\mu \mu )$ for each slice of rapidity. The *top* (*bottom*) *row* shows the 7 TeV (8 TeV) results. For each increasing rapidity slice, an additional scaling factor of 10 is applied to the plotted points for visual clarity. The centre of each bin on the *horizontal axis* represents the mean of the weighted $p_{\text {T}}$ distribution. The *horizontal error bars* represent the range of $p_{\text {T}}$ for the bin, and the *vertical error bar* covers the statistical and systematic uncertainty (with the same multiplicative scaling applied). The NLO NRQCD theory predictions are also shown

**Fig. 8:**
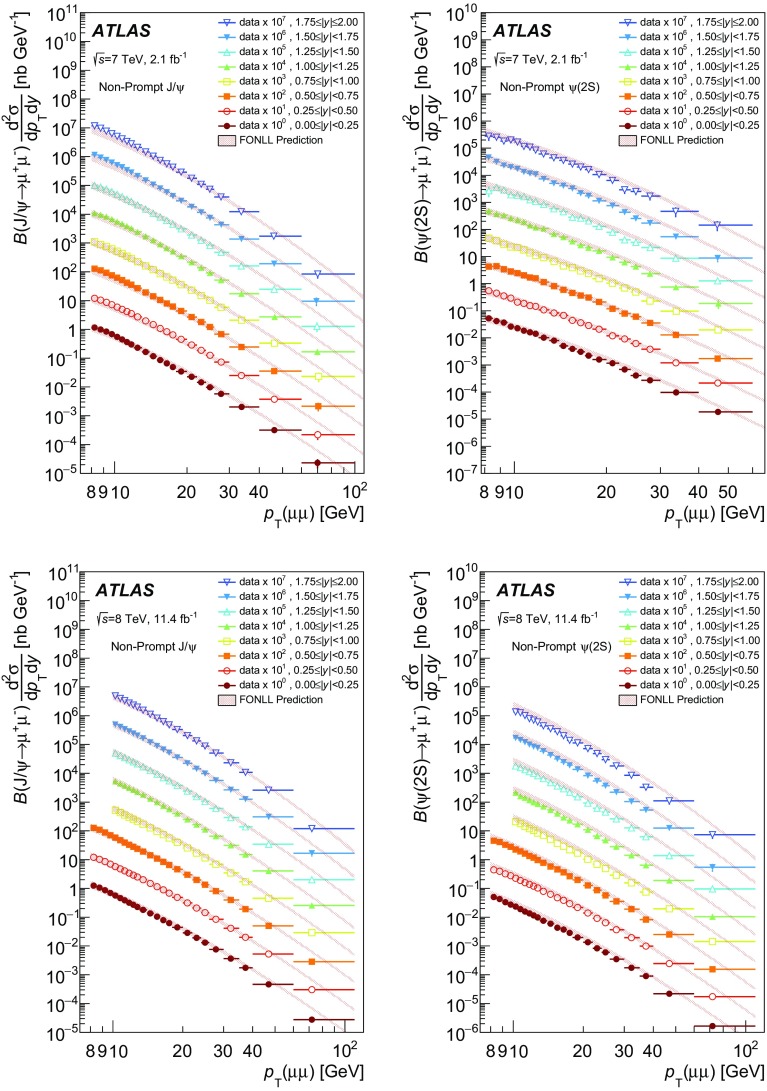
The differential non-prompt cross-section times dimuon branching fraction of $J/\psi$ (*left*) and $\psi (2\mathrm {S})$ (*right*) as a function of $p_{\text {T}}(\mu \mu )$ for each slice of rapidity. The *top* (*bottom*) *row* shows the 7 TeV (8 TeV) results. For each increasing rapidity slice, an additional scaling factor of 10 is applied to the plotted points for visual clarity. The centre of each bin on the *horizontal axis* represents the mean of the weighted $p_{\text {T}}$ distribution. The *horizontal error bars* represent the range of $p_{\text {T}}$ for the bin, and the *vertical error bar* covers the statistical and systematic uncertainty (with the same multiplicative scaling applied). The FONLL theory predictions are also shown

Figures 7 and 8 show respectively the prompt and non-prompt differential cross-sections of $J/\psi$ and $\psi (2\mathrm {S})$ as functions of $p_{\text {T}}$ and |*y*|, together with the relevant theoretical predictions, which are described below.

*Non-prompt production fractions*

**Fig. 9:**
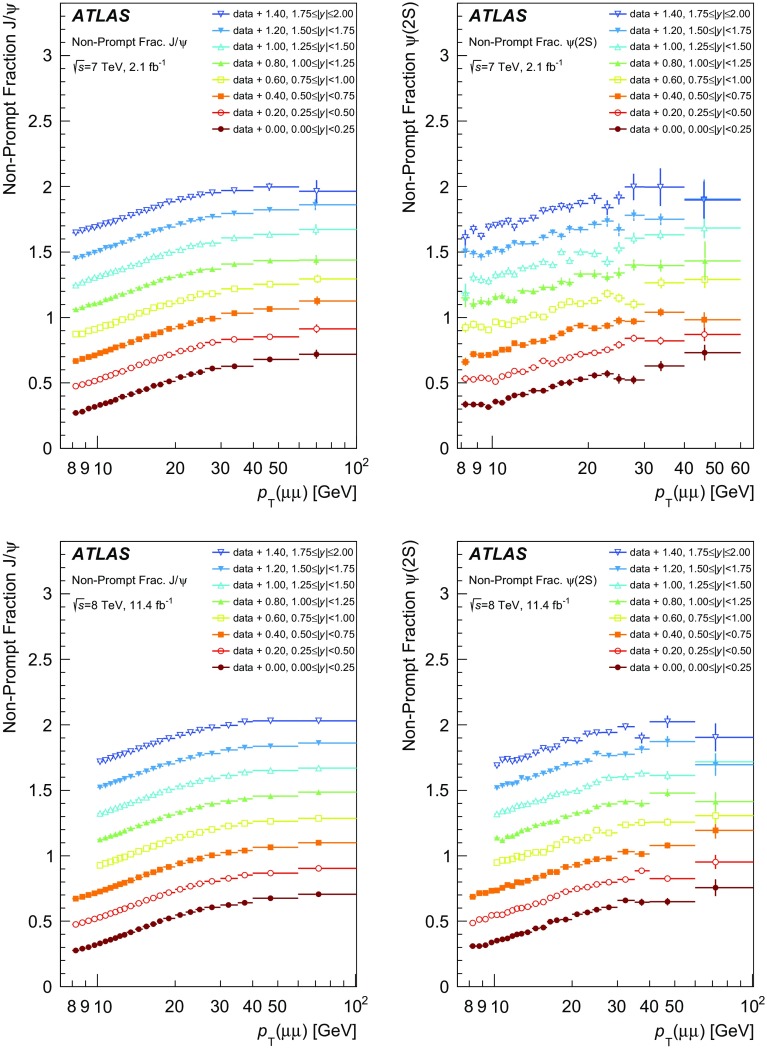
The non-prompt fraction of $J/\psi$ (*left*) and $\psi (2\mathrm {S})$ (*right*), as a function of $p_{\text {T}}(\mu \mu )$ for each slice of rapidity. The *top* (*bottom*) *row* shows the 7 TeV (8 TeV) results. For each increasing rapidity slice, an additional factor of 0.2 is applied to the plotted points for visual clarity. The centre of each bin on the *horizontal axis* represents the mean of the weighted $p_{\text {T}}$ distribution. The *horizontal error bars* represent the range of $p_{\text {T}}$ for the bin, and the *vertical error bar* covers the statistical and systematic uncertainty (with the same multiplicative scaling applied)

The results for the fractions of non-prompt production relative to the inclusive production of $J/\psi$ and $\psi (2\mathrm {S})$  are presented as a function of $p_{\text {T}}$ for slices of rapidity in Fig. 9. In each rapidity slice, the non-prompt fraction is seen to increase as a function of $p_{\text {T}}$ and has no strong dependence on either rapidity or centre-of-mass energy.

*Production ratios of* $\psi (2\mathrm {S})$ *to* $J/\psi$

**Fig. 10:**
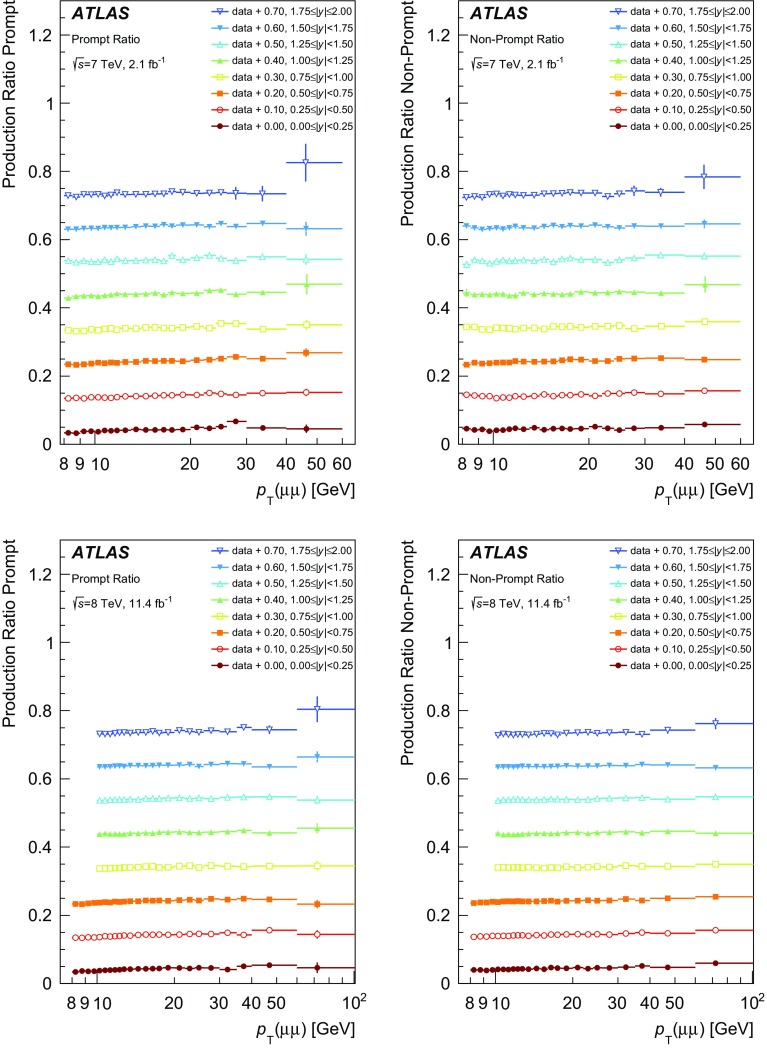
The ratio of $\psi (2\mathrm {S})$ to $J/\psi$ production times dimuon branching fraction for prompt (*left*) and non-prompt (*right*) processes as a function of $p_{\text {T}}(\mu \mu )$ for each of the slices of rapidity. For each increasing rapidity slice, an additional factor of 0.1 is applied to the plotted points for visual clarity. The *top* (*bottom*) *row* shows the 7 TeV (8 TeV) results. The centre of each bin on the *horizontal axis* represents the mean of the weighted $p_{\text {T}}$ distribution. The *horizontal error bars* represent the range of $p_{\text {T}}$ for the bin, and the *vertical error bar* covers the statistical and systematic uncertainty

Figure 10 shows the ratios of $\psi (2\mathrm {S})$ to $J/\psi$ decaying to a muon pair in prompt and non-prompt processes, presented as a function of $p_{\text {T}}$ for slices of rapidity. The non-prompt ratio is shown to be relatively flat across the considered range of $p_{\text {T}}$, for each slice of rapidity. For the prompt ratio, a slight increase as a function of $p_{\text {T}}$ is observed, with no strong dependence on rapidity or centre-of-mass energy.

*Comparison with theory*

**Fig. 11:**
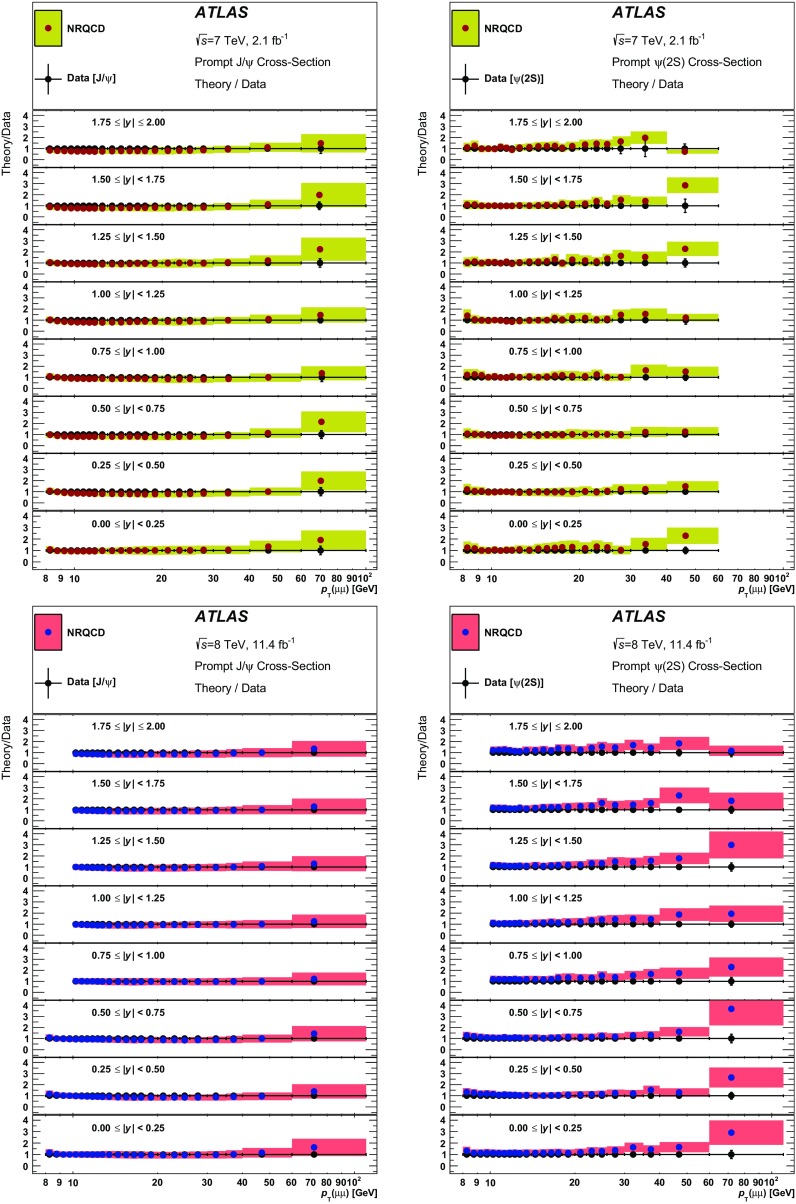
The ratios of the NRQCD theoretical predictions to data are presented for the differential prompt cross-section of $J/\psi$ (*left*) and $\psi (2\mathrm {S})$ (*right*) as a function of $p_{\text {T}}(\mu \mu )$ for each rapidity slice. The *top* (*bottom*) *row* shows the 7 TeV (8 TeV) results. The error on the data is the relative error of each data point, while the *error bars* on the theory prediction are the relative error of each theory point

For prompt production, as shown in Fig. 11, the ratio of the NLO NRQCD theory calculations [60] to data, as a function of $p_{\text {T}}$ and in slices of rapidity, is provided for $J/\psi$ and $\psi (2\mathrm {S})$ at both the 7 and 8 TeV centre-of-mass energies. The theory predictions are based on the long-distance matrix elements (LDMEs) from Refs. [60, 61], with uncertainties originating from the choice of scale, charm quark mass and LDMEs (see Refs. [60, 61] for more details). Figure 11 shows fair agreement between the theoretical calculation and the data points for the whole $p_{\text {T}}$ range. The ratio of theory to data does not depend on rapidity.

**Fig. 12:**
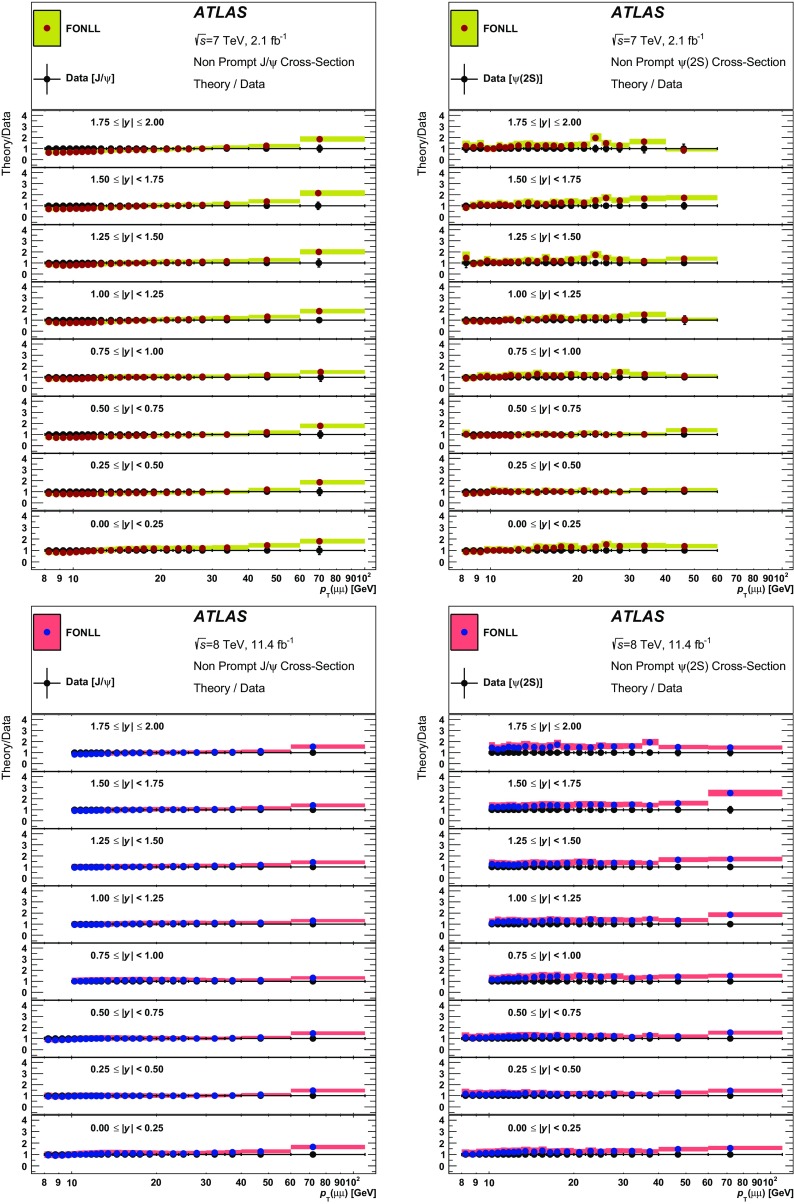
The ratio of the FONLL theoretical predictions to data are presented for the differential non-prompt cross-section of $J/\psi$ (*left*) and $\psi (2\mathrm {S})$ (*right*) as a function of $p_{\text {T}}(\mu \mu )$ for each rapidity slice. The *top* (*bottom*) *row* shows the 7 TeV (8 TeV) results. The error on the data is the relative error of each data point, while the *error bars* on the theory prediction are the relative error of each theory point

For non-prompt $\psi$ production, comparisons are made to FONLL theoretical predictions [1, 2], which describe the production of *b*-hadrons followed by their decay into $\psi +X$. Figure 12 shows the ratios of $J/\psi$ and $\psi (2\mathrm {S})$ FONLL predictions to data, as a function of $p_{\text {T}}$ and in slices of rapidity, for centre-of-mass energies of 7 and 8 TeV. For $J/\psi$, agreement is generally good, but the theory predicts slightly harder $p_{\text {T}}$ spectra than observed in the data. For $\psi (2\mathrm {S})$, the shapes of data and theory appear to be in satisfactory agreement, but the theory predicts higher yields than in the data. There is no observed dependence on rapidity in the comparisons between theory and data for non-prompt $J/\psi$ and $\psi (2\mathrm {S})$ production.

*Comparison of cross-sections* 8 TeV *with* 7 TeV

It is interesting to compare the cross-section results between the two centre-of-mass energies, both for data and the theoretical predictions.

**Fig. 13:**
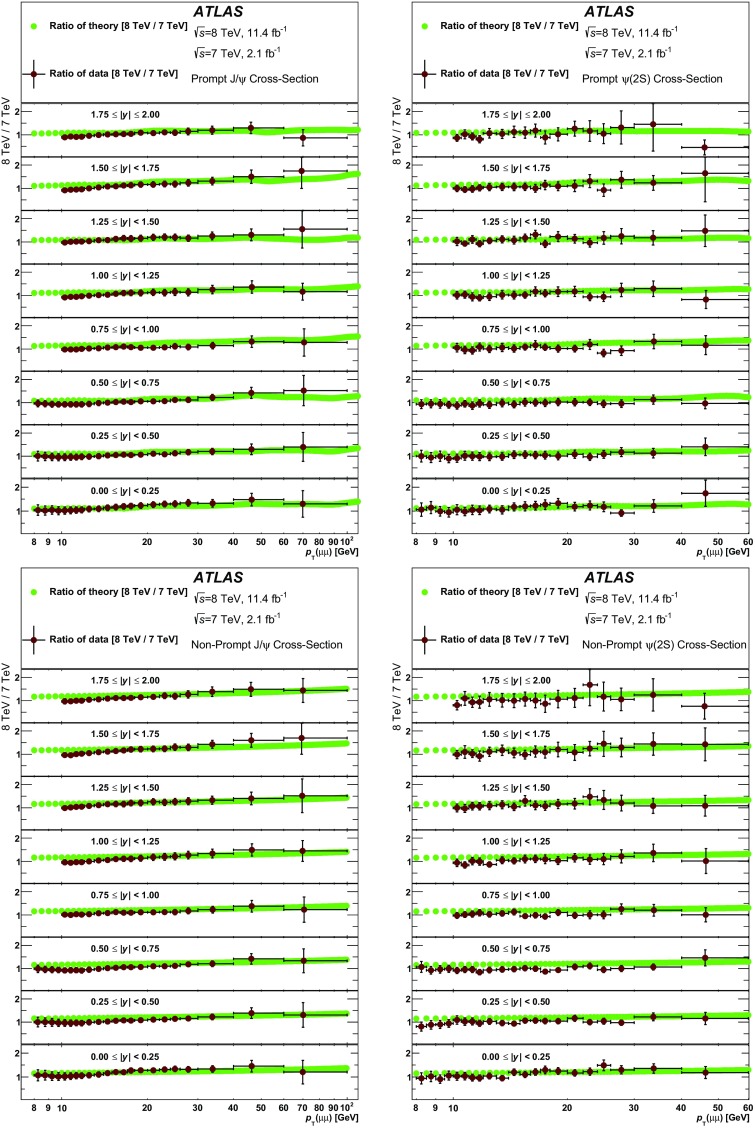
The ratio of the 8 and 7 TeV differential cross-sections are presented for prompt (*top*) and non-prompt (*bottom*) $J/\psi$ (left) and $\psi (2\mathrm {S})$ (*right*) for both data (*red points* with *error bars*) and theoretical predictions (*green points*). The theoretical predictions used are NRQCD for prompt and FONLL for non-prompt production. The uncertainty on the data ratio does not account for possible correlations between 7 and 8 TeV data, and no uncertainty is shown for the ratio of theory predictions

Figure 13 shows the 8–7 TeV cross-section ratios of prompt and non-prompt $J/\psi$ and $\psi (2\mathrm {S})$ for both data sets. For the theoretical ratios the uncertainties are neglected here, since the high correlation between them results in large cancellations.

**Table 4:** Mean weight correction factor for $J/\psi$ under the “longitudinal” spin-alignment hypothesis for 7 TeV

$p_{\text {T}}$ [GeV]	Absolute Rapidity Range							
	0.00–0.25	0.25–0.50	0.50–0.75	0.75–1.00	1.00–1.25	1.25–1.50	1.50–1.75	1.75–2.00
8.00–8.50	0.666	0.672	0.674	0.680	0.688	0.690	0.690	0.690
8.50–9.00	0.670	0.674	0.678	0.685	0.689	0.694	0.694	0.698
9.00–9.50	0.673	0.676	0.680	0.687	0.693	0.697	0.698	0.700
9.50–10.00	0.675	0.678	0.683	0.689	0.694	0.697	0.701	0.703
10.00–10.50	0.679	0.681	0.687	0.692	0.697	0.699	0.702	0.706
10.50–11.00	0.682	0.686	0.691	0.696	0.700	0.702	0.704	0.708
11.00–11.50	0.688	0.689	0.694	0.699	0.701	0.705	0.708	0.710
11.50–12.00	0.692	0.695	0.698	0.702	0.706	0.708	0.710	0.712
12.00–13.00	0.698	0.700	0.703	0.707	0.711	0.713	0.715	0.717
14.00–15.00	0.716	0.717	0.720	0.722	0.725	0.727	0.728	0.730
15.00–16.00	0.724	0.726	0.728	0.729	0.732	0.734	0.735	0.737
16.00–17.00	0.733	0.733	0.735	0.737	0.739	0.741	0.742	0.744
17.00–18.00	0.740	0.741	0.743	0.744	0.746	0.747	0.749	0.750
18.00–20.00	0.751	0.752	0.753	0.754	0.756	0.758	0.758	0.760
20.00–22.00	0.765	0.765	0.766	0.767	0.769	0.770	0.771	0.772
22.00–24.00	0.777	0.777	0.778	0.780	0.781	0.781	0.782	0.783
24.00–26.00	0.789	0.789	0.790	0.790	0.791	0.792	0.793	0.794
26.00–30.00	0.803	0.803	0.804	0.804	0.805	0.806	0.806	0.807
30.00–40.00	0.827	0.827	0.828	0.828	0.829	0.829	0.830	0.831
40.00–60.00	0.863	0.863	0.864	0.864	0.864	0.865	0.865	0.866
60.00–100.00	0.902	0.904	0.904	0.903	0.904	0.904	0.902	0.906

Due to a finer granularity in $p_{\text {T}}$ for the 8 TeV data, a weighted average of the 8 TeV results is taken across equivalent intervals of the 7 TeV data to enable direct comparisons. Both data and theoretical predictions agree that the ratios become larger with increasing $p_{\text {T}}$, however at the lower edge of the $p_{\text {T}}$ range the data tends to be slightly below theory.

## Summary and conclusions

The prompt and non-prompt production cross-sections, the non-prompt production fraction of the $J/\psi$ and $\psi (2\mathrm {S})$ decaying into two muons, the ratio of prompt $\psi (2\mathrm {S})$ to prompt $J/\psi$ production, and the ratio of non-prompt $\psi (2\mathrm {S})$ to non-prompt $J/\psi$ production were measured in the rapidity range $|y|<2.0$ for transverse momenta between 8 and 110 GeV. This measurement was carried out using 2.1$\mathrm{fb}^{-1}$($11.4\mathrm{fb}^{-1}$) of *pp* collision data at a centre-of-mass energy of 7 TeV (8 TeV) recorded by the ATLAS experiment at the LHC. It is the latest in a series of related measurements of the production of charmonium states made by ATLAS. In line with previous measurements, the central values were obtained assuming isotropic $\psi \rightarrow \mu \mu$ decays. Correction factors for these cross-sections, computed for a number of extreme spin-alignment scenarios, are between $-35$ and $+100\,\%$ at the lowest transverse momenta studied, and between $-14$ and $+9\,\%$ at the highest transverse momenta, depending on the specific scenario.

The ATLAS measurements presented here extend the range of existing measurements to higher transverse momenta, and to a higher collision energy of $\sqrt{s} = 8$ TeV, and, in overlapping phase-space regions, are consistent with previous measurements made by ATLAS and other LHC experiments. For the prompt production mechanism, the predictions from the NRQCD model, which includes colour-octet contributions with various matrix elements tuned to earlier collider data, are found to be in good agreement with the observed data points. For the non-prompt production, the fixed-order next-to-leading-logarithm calculations reproduce the data reasonably well, with a slight overestimation of the differential cross-sections at the highest transverse momenta reached in this analysis.

**Table 5:** Mean weight correction factor for $J/\psi$ under the “transverse zero” spin-alignment hypothesis for 7 TeV

$p_{\text {T}}$ [GeV]	Absolute Rapidity Range							
	0.00–0.25	0.25–0.50	0.50–0.75	0.75–1.00	1.00–1.25	1.25–1.50	1.50–1.75	1.75–2.00
8.00–8.50	1.336	1.324	1.315	1.309	1.299	1.297	1.296	1.298
8.50–9.00	1.329	1.323	1.310	1.300	1.291	1.284	1.280	1.284
9.00–9.50	1.326	1.315	1.303	1.295	1.289	1.281	1.279	1.276
9.50–10.00	1.317	1.311	1.300	1.289	1.284	1.276	1.276	1.272
10.00–10.50	1.310	1.304	1.297	1.290	1.280	1.276	1.273	1.269
10.50–11.00	1.302	1.298	1.291	1.285	1.276	1.271	1.267	1.268
11.00–11.50	1.296	1.290	1.284	1.278	1.271	1.266	1.263	1.261
11.50–12.00	1.288	1.284	1.277	1.274	1.265	1.261	1.260	1.257
12.00–13.00	1.276	1.273	1.268	1.263	1.257	1.255	1.251	1.250
13.00–14.00	1.263	1.260	1.254	1.250	1.247	1.244	1.243	1.240
14.00–15.00	1.248	1.246	1.244	1.240	1.236	1.233	1.233	1.230
15.00–16.00	1.237	1.233	1.231	1.228	1.225	1.223	1.223	1.221
16.00–17.00	1.224	1.222	1.221	1.219	1.216	1.213	1.212	1.212
17.00–18.00	1.213	1.213	1.211	1.208	1.205	1.204	1.204	1.203
18.00–20.00	1.200	1.198	1.197	1.196	1.194	1.192	1.192	1.190
20.00–22.00	1.183	1.182	1.180	1.180	1.178	1.177	1.176	1.175
22.00–24.00	1.168	1.167	1.166	1.165	1.164	1.164	1.163	1.163
24.00–26.00	1.155	1.155	1.154	1.154	1.153	1.152	1.151	1.150
26.00–30.00	1.140	1.140	1.140	1.139	1.138	1.138	1.138	1.137
30.00–40.00	1.117	1.117	1.117	1.116	1.116	1.116	1.115	1.115
40.00–60.00	1.087	1.087	1.086	1.086	1.086	1.085	1.085	1.085
60.00–100.00	1.057	1.056	1.057	1.057	1.056	1.056	1.057	1.055

**Table 6:** Mean weight correction factor for $J/\psi$ under the “transverse positive” spin-alignment transverse positive hypothesis for 7 TeV

$p_{\text {T}}$ [GeV]	Absolute Rapidity Range							
	0.00–0.25	0.25–0.50	0.50–0.75	0.75–1.00	1.00–1.25	1.25–1.50	1.50–1.75	1.75–2.00
8.00–8.50	1.693	1.694	1.700	1.711	1.727	1.720	1.720	1.747
8.50–9.00	1.561	1.564	1.564	1.568	1.568	1.568	1.571	1.673
9.00–9.50	1.468	1.468	1.465	1.466	1.470	1.466	1.471	1.519
9.50–10.00	1.418	1.416	1.417	1.417	1.421	1.417	1.423	1.453
10.00–10.50	1.383	1.383	1.387	1.389	1.390	1.389	1.391	1.406
10.50–11.00	1.360	1.362	1.365	1.364	1.364	1.362	1.362	1.380
11.00–11.50	1.344	1.342	1.342	1.344	1.344	1.344	1.346	1.355
11.50–12.00	1.326	1.326	1.327	1.329	1.327	1.327	1.329	1.334
12.00–13.00	1.307	1.308	1.307	1.308	1.308	1.308	1.308	1.312
13.00–14.00	1.285	1.287	1.285	1.285	1.285	1.286	1.285	1.288
14.00–15.00	1.266	1.266	1.267	1.267	1.266	1.266	1.266	1.268
15.00–16.00	1.250	1.249	1.250	1.251	1.250	1.249	1.250	1.250
16.00–17.00	1.234	1.235	1.235	1.235	1.235	1.235	1.235	1.235
17.00–18.00	1.222	1.223	1.223	1.223	1.222	1.222	1.223	1.222
18.00–20.00	1.206	1.206	1.206	1.207	1.207	1.207	1.206	1.205
20.00–22.00	1.187	1.187	1.187	1.188	1.187	1.187	1.187	1.186
22.00–24.00	1.171	1.171	1.171	1.171	1.171	1.171	1.171	1.171
24.00–26.00	1.158	1.158	1.158	1.158	1.158	1.158	1.158	1.156
26.00–30.00	1.142	1.142	1.142	1.142	1.142	1.142	1.142	1.141
30.00–40.00	1.118	1.118	1.118	1.118	1.118	1.118	1.118	1.117
40.00–60.00	1.087	1.087	1.087	1.086	1.087	1.086	1.086	1.086
60.00–100.00	1.058	1.057	1.057	1.057	1.057	1.056	1.058	1.056

**Table 7:** Mean weight correction factor for $J/\psi$ under the “transverse negative” spin-alignment hypothesis for 7 TeV

$p_{\text {T}}$ [GeV]	Absolute Rapidity Range							
	0.00–0.25	0.25–0.50	0.50–0.75	0.75–1.00	1.00–1.25	1.25–1.50	1.50–1.75	1.75–2.00
8.00–8.50	1.030	1.020	1.004	0.995	0.992	0.981	0.973	0.949
8.50–9.00	1.157	1.148	1.134	1.113	1.101	1.092	1.079	1.055
9.00–9.50	1.207	1.196	1.176	1.161	1.147	1.138	1.130	1.107
9.50–10.00	1.231	1.219	1.202	1.186	1.174	1.162	1.158	1.138
10.00–10.50	1.243	1.231	1.217	1.202	1.189	1.181	1.175	1.158
10.50–11.00	1.246	1.239	1.228	1.213	1.200	1.191	1.186	1.174
11.00–11.50	1.252	1.242	1.230	1.218	1.205	1.198	1.192	1.181
11.50–12.00	1.251	1.243	1.229	1.222	1.208	1.202	1.197	1.187
12.00–13.00	1.247	1.240	1.230	1.221	1.211	1.205	1.200	1.193
13.00–14.00	1.240	1.235	1.227	1.218	1.211	1.206	1.202	1.197
14.00–15.00	1.232	1.227	1.221	1.215	1.207	1.203	1.200	1.195
15.00–16.00	1.223	1.219	1.213	1.207	1.201	1.198	1.196	1.193
16.00–17.00	1.213	1.210	1.206	1.201	1.196	1.193	1.191	1.189
17.00–18.00	1.204	1.203	1.199	1.194	1.189	1.187	1.186	1.183
18.00–20.00	1.193	1.191	1.188	1.185	1.181	1.179	1.177	1.176
20.00–22.00	1.178	1.177	1.174	1.172	1.169	1.167	1.166	1.164
22.00–24.00	1.164	1.163	1.162	1.159	1.157	1.156	1.156	1.154
24.00–26.00	1.153	1.152	1.150	1.149	1.148	1.147	1.145	1.144
26.00–30.00	1.139	1.138	1.137	1.136	1.135	1.134	1.133	1.132
30.00–40.00	1.116	1.116	1.115	1.114	1.114	1.113	1.113	1.112
40.00–60.00	1.086	1.086	1.086	1.085	1.085	1.084	1.084	1.084
60.00–100.00	1.057	1.056	1.056	1.056	1.056	1.056	1.057	1.055

**Table 8:** Mean weight correction factor for $J/\psi$ under the “off-($\lambda _{\theta }$–$\lambda _{\phi }$)-plane positive” spin-alignment hypothesis for 7 TeV

$p_{\text {T}}$ [GeV]	Absolute Rapidity Range							
	0.00–0.25	0.25–0.50	0.50–0.75	0.75–1.00	1.00–1.25	1.25–1.50	1.50–1.75	1.75–2.00
8.00–8.50	1.015	1.047	1.073	1.094	1.113	1.120	1.124	1.122
8.50–9.00	1.020	1.058	1.087	1.110	1.125	1.134	1.142	1.144
9.00–9.50	1.019	1.056	1.084	1.107	1.127	1.138	1.144	1.145
9.50–10.00	1.017	1.053	1.081	1.105	1.122	1.129	1.140	1.142
10.00–10.50	1.017	1.049	1.077	1.100	1.115	1.125	1.132	1.136
10.50–11.00	1.014	1.048	1.075	1.095	1.109	1.118	1.124	1.130
11.00–11.50	1.015	1.044	1.069	1.088	1.103	1.112	1.117	1.122
11.50–12.00	1.014	1.043	1.066	1.083	1.096	1.105	1.112	1.115
12.00–13.00	1.012	1.038	1.060	1.076	1.089	1.097	1.101	1.105
13.00–14.00	1.012	1.035	1.053	1.068	1.079	1.087	1.090	1.093
14.00–15.00	1.010	1.031	1.048	1.061	1.070	1.076	1.080	1.083
15.00–16.00	1.010	1.028	1.043	1.054	1.063	1.068	1.072	1.074
16.00–17.00	1.009	1.025	1.039	1.049	1.056	1.062	1.065	1.067
17.00–18.00	1.009	1.023	1.035	1.044	1.051	1.055	1.059	1.060
18.00–20.00	1.007	1.019	1.030	1.039	1.045	1.049	1.051	1.053
20.00–22.00	1.007	1.016	1.025	1.032	1.038	1.041	1.043	1.044
22.00–24.00	1.005	1.014	1.021	1.027	1.031	1.035	1.036	1.037
24.00–26.00	1.005	1.012	1.018	1.024	1.027	1.030	1.032	1.032
26.00–30.00	1.004	1.010	1.015	1.019	1.023	1.025	1.026	1.026
30.00–40.00	1.003	1.007	1.011	1.014	1.016	1.017	1.018	1.019
40.00–60.00	1.002	1.004	1.006	1.008	1.009	1.010	1.010	1.010
60.00–100.00	1.001	1.002	1.003	1.003	1.004	1.004	1.005	1.005

**Table 9:** Mean weight correction factor for $J/\psi$ under the “off-($\lambda _{\theta }$–$\lambda _{\phi }$)-plane negative” spin-alignment hypothesis for 7 TeV

$p_{\text {T}}$ [GeV]	Absolute Rapidity Range							
	0.00–0.25	0.25–0.50	0.50–0.75	0.75–1.00	1.00–1.25	1.25–1.50	1.50–1.75	1.75–2.00
8.00–8.50	0.984	0.956	0.932	0.920	0.912	0.904	0.898	0.905
8.50–9.00	0.981	0.948	0.925	0.910	0.898	0.892	0.886	0.891
9.00–9.50	0.983	0.950	0.925	0.910	0.901	0.893	0.889	0.888
9.50–10.00	0.983	0.951	0.929	0.912	0.903	0.894	0.892	0.891
10.00–10.50	0.985	0.953	0.932	0.918	0.907	0.900	0.896	0.894
10.50–11.00	0.984	0.957	0.936	0.922	0.910	0.904	0.900	0.899
11.00–11.50	0.985	0.958	0.939	0.927	0.915	0.909	0.906	0.903
11.50–12.00	0.987	0.961	0.942	0.929	0.919	0.912	0.910	0.907
12.00–13.00	0.987	0.963	0.945	0.934	0.925	0.920	0.915	0.913
13.00–14.00	0.989	0.968	0.951	0.940	0.932	0.927	0.924	0.922
14.00–15.00	0.990	0.971	0.957	0.946	0.938	0.934	0.931	0.929
15.00–16.00	0.992	0.974	0.961	0.951	0.944	0.940	0.937	0.936
16.00–17.00	0.991	0.976	0.964	0.955	0.949	0.945	0.943	0.941
17.00–18.00	0.992	0.978	0.968	0.959	0.953	0.949	0.948	0.946
18.00–20.00	0.993	0.981	0.971	0.964	0.959	0.956	0.954	0.953
20.00–22.00	0.994	0.984	0.976	0.970	0.965	0.962	0.961	0.960
22.00–24.00	0.994	0.986	0.979	0.974	0.970	0.968	0.966	0.965
24.00–26.00	0.995	0.988	0.982	0.977	0.974	0.972	0.971	0.970
26.00–30.00	0.996	0.990	0.985	0.981	0.978	0.977	0.976	0.975
30.00–40.00	0.997	0.993	0.990	0.987	0.985	0.983	0.983	0.982
40.00–60.00	0.998	0.996	0.994	0.993	0.991	0.991	0.990	0.990
60.00–100.00	0.999	0.998	0.997	0.997	0.996	0.996	0.995	0.995

**Table 10:** Mean weight correction factor for $\psi (2\mathrm {S})$ under the “longitudinal” spin-alignment hypothesis for 7 TeV

$p_{\text {T}}$ [GeV]	Absolute Rapidity Range							
	0.00–0.25	0.25–0.50	0.50–0.75	0.75–1.00	1.00–1.25	1.25–1.50	1.50–1.75	1.75–2.00
8.00–8.50	0.670	0.678	0.685	0.692	0.701	0.707	0.713	0.709
8.50–9.00	0.676	0.681	0.688	0.698	0.703	0.709	0.712	0.713
9.00–9.50	0.678	0.683	0.691	0.700	0.708	0.713	0.717	0.718
9.50–10.00	0.680	0.684	0.693	0.699	0.708	0.710	0.720	0.722
10.00–10.50	0.684	0.687	0.695	0.704	0.707	0.713	0.720	0.725
10.50–11.00	0.687	0.691	0.698	0.705	0.712	0.714	0.719	0.728
11.00–11.50	0.692	0.695	0.701	0.709	0.713	0.717	0.722	0.728
11.50–12.00	0.696	0.700	0.704	0.711	0.717	0.719	0.724	0.729
12.00–13.00	0.701	0.705	0.710	0.716	0.720	0.724	0.727	0.731
13.00–14.00	0.711	0.714	0.718	0.722	0.727	0.730	0.732	0.734
14.00–15.00	0.719	0.722	0.725	0.730	0.732	0.736	0.739	0.742
15.00–16.00	0.727	0.729	0.733	0.735	0.740	0.741	0.745	0.745
16.00–17.00	0.736	0.738	0.740	0.743	0.746	0.748	0.749	0.753
17.00–18.00	0.742	0.744	0.748	0.750	0.753	0.754	0.759	0.760
18.00–20.00	0.753	0.755	0.758	0.760	0.762	0.763	0.764	0.769
20.00–22.00	0.766	0.767	0.770	0.773	0.774	0.776	0.776	0.778
22.00–24.00	0.778	0.782	0.780	0.784	0.785	0.782	0.790	0.788
24.00–26.00	0.791	0.791	0.795	0.795	0.795	0.799	0.798	0.798
26.00–30.00	0.806	0.805	0.805	0.809	0.808	0.810	0.810	0.812
30.00–40.00	0.829	0.830	0.830	0.830	0.828	0.832	0.830	0.830
40.00–60.00	0.864	0.865	0.867	0.864	0.868	0.867	0.861	0.953

**Table 11:** Mean weight correction factor for $\psi (2\mathrm {S})$ under the “transverse zero” spin-alignment hypothesis for 7 TeV

$p_{\text {T}}$ [GeV]	Absolute Rapidity Range							
	0.00–0.25	0.25–0.50	0.50–0.75	0.75–1.00	1.00–1.25	1.25–1.50	1.50–1.75	1.75–2.00
8.00–8.50	1.328	1.311	1.300	1.284	1.274	1.267	1.261	1.265
8.50–9.00	1.318	1.309	1.293	1.279	1.268	1.263	1.252	1.259
9.00–9.50	1.317	1.303	1.287	1.273	1.267	1.256	1.249	1.250
9.50–10.00	1.310	1.301	1.286	1.275	1.262	1.255	1.248	1.247
10.00–10.50	1.303	1.294	1.283	1.271	1.265	1.257	1.248	1.243
10.50–11.00	1.295	1.289	1.279	1.271	1.259	1.254	1.246	1.240
11.00–11.50	1.289	1.282	1.273	1.264	1.254	1.249	1.242	1.238
11.50–12.00	1.282	1.276	1.267	1.260	1.249	1.246	1.240	1.234
12.00–13.00	1.271	1.266	1.259	1.250	1.244	1.241	1.236	1.232
13.00–14.00	1.258	1.252	1.246	1.239	1.234	1.232	1.229	1.226
14.00–15.00	1.244	1.240	1.237	1.230	1.228	1.221	1.219	1.216
15.00–16.00	1.234	1.229	1.224	1.221	1.216	1.214	1.211	1.211
16.00–17.00	1.220	1.217	1.213	1.211	1.208	1.205	1.205	1.202
17.00–18.00	1.211	1.210	1.205	1.202	1.198	1.197	1.193	1.193
18.00–20.00	1.197	1.194	1.191	1.190	1.188	1.187	1.186	1.181
20.00–22.00	1.181	1.180	1.176	1.174	1.174	1.171	1.172	1.169
22.00–24.00	1.167	1.163	1.164	1.161	1.160	1.164	1.155	1.159
24.00–26.00	1.153	1.154	1.149	1.148	1.149	1.145	1.147	1.147
26.00–30.00	1.137	1.139	1.138	1.135	1.136	1.134	1.135	1.133
30.00–40.00	1.115	1.115	1.115	1.115	1.116	1.114	1.116	1.116
40.00–60.00	1.086	1.085	1.083	1.086	1.083	1.084	1.089	1.028

**Table 12:** Mean weight correction factor for $\psi (2\mathrm {S})$ under the “transverse positive” spin-alignment hypothesis for 7 TeV

$p_{\text {T}}$ [GeV]	Absolute Rapidity Range							
	0.00–0.25	0.25–0.50	0.50–0.75	0.75–1.00	1.00–1.25	1.25–1.50	1.50–1.75	1.75–2.00
8.00–8.50	2.009	2.007	1.986	1.994	1.964	1.936	1.949	1.967
8.50–9.00	1.614	1.617	1.617	1.613	1.618	1.624	1.606	1.872
9.00–9.50	1.504	1.502	1.496	1.493	1.500	1.499	1.494	1.741
9.50–10.00	1.445	1.443	1.440	1.445	1.440	1.441	1.436	1.621
10.00–10.50	1.404	1.401	1.403	1.401	1.412	1.406	1.400	1.507
10.50–11.00	1.374	1.377	1.378	1.378	1.377	1.378	1.372	1.447
11.00–11.50	1.355	1.352	1.352	1.353	1.352	1.353	1.350	1.409
11.50–12.00	1.335	1.334	1.335	1.335	1.332	1.336	1.331	1.375
12.00–13.00	1.314	1.313	1.312	1.312	1.313	1.314	1.312	1.343
13.00–14.00	1.289	1.289	1.287	1.286	1.286	1.288	1.288	1.311
14.00–15.00	1.268	1.267	1.269	1.267	1.269	1.265	1.265	1.280
15.00–16.00	1.253	1.250	1.249	1.252	1.250	1.250	1.248	1.262
16.00–17.00	1.234	1.234	1.234	1.234	1.235	1.235	1.236	1.241
17.00–18.00	1.224	1.224	1.222	1.222	1.220	1.222	1.218	1.224
18.00–20.00	1.206	1.205	1.204	1.205	1.205	1.207	1.206	1.204
20.00–22.00	1.187	1.187	1.186	1.184	1.186	1.184	1.186	1.186
22.00–24.00	1.171	1.169	1.171	1.169	1.169	1.174	1.166	1.170
24.00–26.00	1.157	1.158	1.155	1.155	1.156	1.153	1.156	1.156
26.00–30.00	1.140	1.142	1.142	1.139	1.141	1.140	1.141	1.139
30.00–40.00	1.116	1.117	1.117	1.117	1.119	1.117	1.119	1.119
40.00–60.00	1.087	1.086	1.084	1.087	1.085	1.085	1.091	1.029

**Table 13:** Mean weight correction factor for $\psi (2\mathrm {S})$ under the “transverse negative” spin-alignment hypothesis for 7 TeV

$p_{\text {T}}$ [GeV]	Absolute Rapidity Range							
	0.00–0.25	0.25–0.50	0.50–0.75	0.75–1.00	1.00–1.25	1.25–1.50	1.50–1.75	1.75–2.00
8.00–8.50	0.998	0.986	0.970	0.957	0.949	0.941	0.935	0.883
8.50–9.00	1.115	1.102	1.084	1.062	1.047	1.039	1.025	0.959
9.00–9.50	1.169	1.154	1.131	1.110	1.096	1.084	1.075	1.007
9.50–10.00	1.200	1.185	1.163	1.144	1.126	1.114	1.105	1.047
10.00–10.50	1.216	1.200	1.181	1.161	1.148	1.137	1.127	1.075
10.50–11.00	1.222	1.212	1.196	1.178	1.161	1.152	1.143	1.097
11.00–11.50	1.230	1.218	1.202	1.185	1.169	1.161	1.152	1.112
11.50–12.00	1.233	1.221	1.205	1.192	1.175	1.169	1.160	1.124
12.00–13.00	1.232	1.222	1.208	1.195	1.184	1.176	1.169	1.141
13.00–14.00	1.228	1.220	1.208	1.196	1.187	1.181	1.176	1.155
14.00–15.00	1.221	1.214	1.207	1.196	1.188	1.181	1.176	1.159
15.00–16.00	1.215	1.208	1.200	1.193	1.184	1.181	1.175	1.165
16.00–17.00	1.205	1.200	1.194	1.187	1.182	1.177	1.175	1.165
17.00–18.00	1.199	1.196	1.188	1.183	1.177	1.174	1.169	1.162
18.00–20.00	1.188	1.184	1.179	1.175	1.170	1.168	1.166	1.158
20.00–22.00	1.174	1.172	1.167	1.162	1.161	1.157	1.157	1.153
22.00–24.00	1.162	1.157	1.158	1.153	1.151	1.153	1.145	1.146
24.00–26.00	1.149	1.149	1.144	1.142	1.142	1.138	1.139	1.138
26.00–30.00	1.135	1.136	1.134	1.130	1.131	1.129	1.129	1.127
30.00–40.00	1.114	1.113	1.113	1.112	1.113	1.110	1.112	1.112
40.00–60.00	1.086	1.085	1.083	1.085	1.082	1.082	1.088	1.028

**Table 14:** Mean weight correction factor for $\psi (2\mathrm {S})$ under the “off-($\lambda _{\theta }$–$\lambda _{\phi }$)-plane positive” spin-alignment hypothesis for 7 TeV

$p_{\text {T}}$ [GeV]	Absolute Rapidity Range							
	0.00–0.25	0.25–0.50	0.50–0.75	0.75–1.00	1.00–1.25	1.25–1.50	1.50–1.75	1.75–2.00
8.00–8.50	1.017	1.052	1.081	1.100	1.118	1.123	1.129	1.106
8.50–9.00	1.023	1.064	1.094	1.118	1.136	1.146	1.151	1.132
9.00–9.50	1.021	1.062	1.093	1.119	1.140	1.150	1.153	1.139
9.50–10.00	1.019	1.060	1.092	1.119	1.135	1.144	1.152	1.146
10.00–10.50	1.020	1.057	1.088	1.112	1.132	1.140	1.146	1.145
10.50–11.00	1.017	1.055	1.085	1.108	1.124	1.134	1.139	1.141
11.00–11.50	1.017	1.052	1.079	1.102	1.118	1.127	1.131	1.137
11.50–12.00	1.017	1.050	1.076	1.096	1.110	1.120	1.126	1.130
12.00–13.00	1.014	1.044	1.069	1.088	1.102	1.111	1.116	1.123
13.00–14.00	1.013	1.041	1.061	1.078	1.091	1.100	1.104	1.111
14.00–15.00	1.012	1.036	1.056	1.070	1.082	1.088	1.092	1.098
15.00–16.00	1.011	1.032	1.049	1.064	1.073	1.079	1.083	1.090
16.00–17.00	1.010	1.029	1.045	1.057	1.065	1.072	1.076	1.080
17.00–18.00	1.010	1.027	1.041	1.051	1.059	1.064	1.068	1.071
18.00–20.00	1.008	1.023	1.035	1.045	1.052	1.057	1.059	1.062
20.00–22.00	1.008	1.019	1.030	1.037	1.044	1.047	1.050	1.052
22.00–24.00	1.006	1.016	1.025	1.032	1.037	1.042	1.042	1.044
24.00–26.00	1.005	1.014	1.021	1.027	1.032	1.034	1.037	1.038
26.00–30.00	1.005	1.012	1.018	1.022	1.027	1.029	1.030	1.031
30.00–40.00	1.003	1.008	1.013	1.016	1.019	1.020	1.022	1.022
40.00–60.00	1.002	1.005	1.007	1.009	1.010	1.011	1.013	1.004

**Table 15:** Mean weight correction factor for $\psi (2\mathrm {S})$ under the “off-($\lambda _{\theta }$–$\lambda _{\phi }$)-plane negative” spin-alignment hypothesis for 7 TeV

$p_{\text {T}}$ [GeV]	Absolute Rapidity Range							
	0.00–0.25	0.25–0.50	0.50–0.75	0.75–1.00	1.00–1.25	1.25–1.50	1.50–1.75	1.75–2.00
8.00–8.50	0.983	0.950	0.931	0.916	0.908	0.902	0.902	0.911
8.50–9.00	0.979	0.944	0.919	0.904	0.892	0.887	0.882	0.898
9.00–9.50	0.981	0.943	0.919	0.901	0.894	0.886	0.883	0.891
9.50–10.00	0.981	0.945	0.922	0.903	0.894	0.886	0.885	0.891
10.00–10.50	0.982	0.948	0.925	0.910	0.897	0.891	0.888	0.890
10.50–11.00	0.982	0.951	0.929	0.913	0.901	0.895	0.891	0.892
11.00–11.50	0.983	0.953	0.931	0.918	0.906	0.900	0.897	0.894
11.50–12.00	0.985	0.955	0.934	0.920	0.910	0.903	0.901	0.897
12.00–13.00	0.985	0.958	0.938	0.925	0.915	0.911	0.906	0.903
13.00–14.00	0.988	0.963	0.944	0.932	0.924	0.918	0.915	0.910
14.00–15.00	0.988	0.966	0.950	0.939	0.930	0.926	0.923	0.918
15.00–16.00	0.990	0.969	0.955	0.944	0.937	0.932	0.929	0.924
16.00–17.00	0.991	0.972	0.959	0.949	0.941	0.937	0.935	0.932
17.00–18.00	0.991	0.975	0.963	0.953	0.947	0.942	0.941	0.938
18.00–20.00	0.992	0.978	0.967	0.959	0.953	0.949	0.947	0.945
20.00–22.00	0.993	0.981	0.972	0.965	0.960	0.957	0.955	0.953
22.00–24.00	0.994	0.984	0.975	0.970	0.966	0.962	0.961	0.959
24.00–26.00	0.995	0.986	0.979	0.974	0.970	0.968	0.966	0.965
26.00–30.00	0.995	0.988	0.983	0.978	0.975	0.973	0.972	0.971
30.00–40.00	0.997	0.992	0.988	0.984	0.982	0.981	0.979	0.979
40.00–60.00	0.998	0.995	0.993	0.991	0.990	0.989	0.988	0.996

**Table 16:** Mean weight correction factor for $J/\psi$ under the “longitudinal” spin-alignment hypothesis for 8 TeV. Those intervals not measured in the analysis at low $p_{\text {T}}$, high rapidity are also excluded here

$p_{\text {T}}$ [GeV]	Absolute Rapidity Range							
	0.00–0.25	0.25–0.50	0.50–0.75	0.75–1.00	1.00–1.25	1.25–1.50	1.50–1.75	1.75–2.00
8.00–8.50	0.672	0.674	0.678	–	–	–	–	–
8.50–9.00	0.670	0.673	0.678	–	–	–	–	–
9.00–9.50	0.671	0.674	0.679	–	–	–	–	–
9.50–10.00	0.674	0.676	0.681	–	–	–	–	–
10.00–10.50	0.676	0.678	0.683	0.686	0.691	0.694	0.695	0.696
10.50–11.00	0.680	0.681	0.686	0.689	0.693	0.696	0.697	0.698
11.00–11.50	0.684	0.685	0.690	0.692	0.695	0.698	0.700	0.701
11.50–12.00	0.688	0.688	0.693	0.695	0.698	0.701	0.702	0.704
12.00–12.50	0.692	0.692	0.696	0.698	0.702	0.704	0.705	0.706
12.50–13.00	0.696	0.696	0.700	0.702	0.705	0.707	0.708	0.710
13.00–14.00	0.702	0.703	0.705	0.707	0.710	0.712	0.713	0.715
14.00–15.00	0.710	0.711	0.713	0.714	0.717	0.719	0.720	0.722
15.00–16.00	0.719	0.719	0.721	0.722	0.724	0.725	0.727	0.729
16.00–17.00	0.726	0.727	0.729	0.729	0.732	0.733	0.734	0.735
17.00–18.00	0.734	0.735	0.736	0.737	0.738	0.740	0.740	0.743
18.00–20.00	0.744	0.745	0.746	0.746	0.748	0.750	0.750	0.752
20.00–22.00	0.758	0.759	0.760	0.759	0.761	0.762	0.763	0.764
22.00–24.00	0.771	0.771	0.772	0.771	0.773	0.774	0.774	0.776
24.00–26.00	0.783	0.783	0.783	0.783	0.784	0.786	0.786	0.787
26.00–30.00	0.797	0.798	0.798	0.797	0.798	0.799	0.800	0.800
30.00–35.00	0.817	0.817	0.817	0.816	0.817	0.818	0.818	0.820
35.00–40.00	0.836	0.836	0.836	0.835	0.835	0.836	0.836	0.840
40.00–60.00	0.862	0.862	0.861	0.861	0.861	0.862	0.862	0.863
60.00–110.00	0.904	0.902	0.903	0.902	0.903	0.904	0.905	0.906

**Table 17:** Mean weight correction factor for $J/\psi$ under the “transverse zero” spin-alignment hypothesis for 8 TeV. Those intervals not measured in the analysis at low $p_{\text {T}}$, high rapidity are also excluded here

$p_{\text {T}}$ [GeV]	Absolute Rapidity Range							
	0.00–0.25	0.25–0.50	0.50–0.75	0.75–1.00	1.00–1.25	1.25–1.50	1.50–1.75	1.75–2.00
8.00–8.50	1.326	1.321	1.311	–	–	–	–	–
8.50–9.00	1.326	1.320	1.309	–	–	–	–	–
9.00–9.50	1.322	1.316	1.306	–	–	–	–	–
9.50–10.00	1.317	1.312	1.302	–	–	–	–	–
10.00–10.50	1.311	1.306	1.297	1.291	1.283	1.278	1.275	1.273
10.50–11.00	1.304	1.300	1.292	1.286	1.279	1.274	1.272	1.269
11.00–11.50	1.297	1.293	1.286	1.280	1.275	1.270	1.268	1.265
11.50–12.00	1.290	1.287	1.280	1.275	1.270	1.266	1.263	1.261
12.00–12.50	1.283	1.280	1.274	1.270	1.264	1.261	1.259	1.257
12.50–13.00	1.276	1.273	1.268	1.264	1.260	1.256	1.254	1.252
13.00–14.00	1.265	1.264	1.259	1.256	1.252	1.249	1.247	1.245
14.00–15.00	1.253	1.251	1.247	1.245	1.241	1.238	1.237	1.235
15.00–16.00	1.240	1.239	1.236	1.234	1.231	1.229	1.227	1.225
16.00–17.00	1.228	1.227	1.225	1.223	1.220	1.218	1.218	1.216
17.00–18.00	1.218	1.217	1.215	1.213	1.211	1.209	1.209	1.206
18.00–20.00	1.204	1.203	1.201	1.201	1.199	1.197	1.196	1.195
20.00–22.00	1.186	1.186	1.185	1.185	1.183	1.182	1.181	1.180
22.00–24.00	1.172	1.171	1.171	1.171	1.169	1.168	1.168	1.167
24.00–26.00	1.159	1.159	1.158	1.158	1.157	1.156	1.156	1.154
26.00–30.00	1.144	1.144	1.143	1.144	1.143	1.142	1.141	1.141
30.00–35.00	1.125	1.125	1.125	1.125	1.124	1.124	1.124	1.122
35.00–40.00	1.108	1.108	1.108	1.108	1.108	1.108	1.107	1.105
40.00–60.00	1.087	1.086	1.087	1.087	1.087	1.087	1.087	1.086
60.00–110.00	1.056	1.057	1.057	1.057	1.057	1.056	1.055	1.055

**Table 18:** Mean weight correction factor for $J/\psi$ under the “transverse positive” spin-alignment hypothesis for 8 TeV. Those intervals not measured in the analysis at low $p_{\text {T}}$, high rapidity are also excluded here

$p_{\text {T}}$ [GeV]	Absolute Rapidity Range							
	0.00–0.25	0.25–0.50	0.50–0.75	0.75–1.00	1.00–1.25	1.25–1.50	1.50–1.75	1.75–2.00
8.00–8.50	1.926	1.933	1.930	–	–	–	–	–
8.50–9.00	1.555	1.558	1.559	–	–	–	–	–
9.00–9.50	1.463	1.464	1.465	–	–	–	–	–
9.50–10.00	1.416	1.418	1.418	–	–	–	–	–
10.00–10.50	1.386	1.388	1.387	1.390	1.390	1.390	1.391	1.411
10.50–11.00	1.363	1.365	1.365	1.367	1.367	1.366	1.368	1.382
11.00–11.50	1.345	1.347	1.346	1.348	1.348	1.348	1.349	1.358
11.50–12.00	1.330	1.331	1.331	1.333	1.333	1.332	1.333	1.340
12.00–12.50	1.316	1.318	1.317	1.319	1.318	1.319	1.319	1.325
12.50–13.00	1.304	1.305	1.305	1.307	1.307	1.307	1.306	1.311
13.00–14.00	1.288	1.290	1.290	1.291	1.291	1.291	1.291	1.293
14.00–15.00	1.270	1.271	1.271	1.272	1.272	1.271	1.272	1.272
15.00–16.00	1.253	1.254	1.254	1.255	1.255	1.255	1.254	1.255
16.00–17.00	1.239	1.240	1.240	1.241	1.240	1.240	1.241	1.240
17.00–18.00	1.227	1.227	1.227	1.228	1.228	1.227	1.228	1.226
18.00–20.00	1.211	1.211	1.211	1.212	1.212	1.211	1.211	1.210
20.00–22.00	1.191	1.192	1.192	1.193	1.193	1.192	1.192	1.192
22.00–24.00	1.175	1.176	1.176	1.177	1.176	1.176	1.176	1.175
24.00–26.00	1.162	1.162	1.162	1.163	1.162	1.162	1.162	1.161
26.00–30.00	1.146	1.146	1.146	1.147	1.146	1.146	1.146	1.146
30.00–35.00	1.126	1.126	1.126	1.127	1.127	1.126	1.126	1.125
35.00–40.00	1.109	1.109	1.109	1.110	1.110	1.109	1.109	1.107
40.00–60.00	1.087	1.087	1.088	1.088	1.088	1.087	1.087	1.087
60.00–110.00	1.056	1.057	1.057	1.057	1.057	1.056	1.056	1.055

**Table 19:** Mean weight correction factor for $J/\psi$ under the “transverse negative” spin-alignment hypothesis for 8 TeV. Those intervals not measured in the analysis at low $p_{\text {T}}$, high rapidity are also excluded here

$p_{\text {T}}$ [GeV]	Absolute Rapidity Range							
	0.00–0.25	0.25–0.50	0.50–0.75	0.75–1.00	1.00–1.25	1.25–1.50	1.50–1.75	1.75–2.00
8.00–8.50	1.026	1.017	1.005	–	–	–	–	–
8.50–9.00	1.157	1.145	1.129	–	–	–	–	–
9.00–9.50	1.207	1.196	1.178	–	–	–	–	–
9.50–10.00	1.231	1.220	1.203	–	–	–	–	–
10.00–10.50	1.244	1.234	1.218	1.204	1.192	1.182	1.177	1.161
10.50–11.00	1.250	1.241	1.227	1.214	1.202	1.193	1.188	1.175
11.00–11.50	1.252	1.244	1.231	1.220	1.209	1.200	1.195	1.184
11.50–12.00	1.253	1.246	1.234	1.223	1.213	1.206	1.201	1.191
12.00–12.50	1.251	1.245	1.234	1.224	1.215	1.208	1.204	1.196
12.50–13.00	1.248	1.243	1.233	1.224	1.216	1.210	1.206	1.199
13.00–14.00	1.243	1.239	1.230	1.222	1.215	1.210	1.206	1.200
14.00–15.00	1.236	1.231	1.224	1.218	1.212	1.207	1.204	1.200
15.00–16.00	1.226	1.223	1.217	1.212	1.207	1.203	1.200	1.197
16.00–17.00	1.218	1.215	1.210	1.206	1.201	1.197	1.195	1.193
17.00–18.00	1.209	1.206	1.202	1.199	1.195	1.192	1.190	1.187
18.00–20.00	1.197	1.195	1.192	1.189	1.186	1.183	1.182	1.180
20.00–22.00	1.182	1.181	1.178	1.177	1.174	1.172	1.170	1.170
22.00–24.00	1.168	1.167	1.166	1.165	1.162	1.161	1.160	1.159
24.00–26.00	1.156	1.156	1.154	1.153	1.152	1.150	1.150	1.148
26.00–30.00	1.142	1.141	1.140	1.140	1.139	1.137	1.137	1.136
30.00–35.00	1.124	1.123	1.123	1.123	1.122	1.121	1.121	1.119
35.00–40.00	1.107	1.107	1.107	1.107	1.107	1.106	1.106	1.103
40.00–60.00	1.087	1.086	1.087	1.086	1.087	1.086	1.086	1.085
60.00–110.00	1.056	1.057	1.057	1.057	1.056	1.056	1.055	1.055

**Table 20:** Mean weight correction factor for $J/\psi$ under the “off-($\lambda _{\theta }$–$\lambda _{\phi }$)-plane positive” spin-alignment hypothesis for 8 TeV. Those intervals not measured in the analysis at low $p_{\text {T}}$, high rapidity are also excluded here

$p_{\text {T}}$ [GeV]	Absolute Rapidity Range							
	0.00–0.25	0.25–0.50	0.50–0.75	0.75–1.00	1.00–1.25	1.25–1.50	1.50–1.75	1.75–2.00
8.00–8.50	1.016	1.048	1.074	–	–	–	–	–
8.50–9.00	1.019	1.056	1.087	–	–	–	–	–
9.00–9.50	1.019	1.055	1.086	–	–	–	–	–
9.50–10.00	1.018	1.053	1.083	–	–	–	–	–
10.00–10.50	1.017	1.051	1.079	1.101	1.117	1.127	1.134	1.138
10.50–11.00	1.016	1.048	1.075	1.096	1.110	1.120	1.126	1.131
11.00–11.50	1.015	1.045	1.071	1.090	1.104	1.113	1.119	1.124
11.50–12.00	1.014	1.043	1.067	1.085	1.098	1.107	1.113	1.117
12.00–12.50	1.014	1.040	1.063	1.080	1.093	1.101	1.106	1.111
12.50–13.00	1.013	1.038	1.059	1.076	1.087	1.095	1.100	1.104
13.00–14.00	1.012	1.035	1.055	1.070	1.080	1.088	1.092	1.096
14.00–15.00	1.011	1.031	1.049	1.062	1.072	1.078	1.082	1.085
15.00–16.00	1.010	1.028	1.044	1.056	1.065	1.070	1.074	1.076
16.00–17.00	1.009	1.025	1.040	1.050	1.058	1.063	1.067	1.069
17.00–18.00	1.008	1.023	1.036	1.046	1.053	1.057	1.060	1.062
18.00–20.00	1.007	1.020	1.031	1.040	1.046	1.050	1.053	1.054
20.00–22.00	1.006	1.017	1.026	1.033	1.039	1.042	1.044	1.045
22.00–24.00	1.005	1.014	1.022	1.028	1.033	1.036	1.038	1.039
24.00–26.00	1.004	1.012	1.019	1.024	1.028	1.030	1.032	1.033
26.00–30.00	1.004	1.010	1.016	1.020	1.023	1.025	1.026	1.027
30.00–35.00	1.003	1.008	1.012	1.015	1.018	1.019	1.020	1.021
35.00–40.00	1.002	1.006	1.009	1.012	1.013	1.015	1.015	1.015
40.00–60.00	1.001	1.004	1.006	1.008	1.009	1.010	1.010	1.010
60.00–110.00	1.001	1.002	1.003	1.003	1.004	1.004	1.004	1.005

**Table 21:** Mean weight correction factor for $J/\psi$ under the “off-($\lambda _{\theta }$–$\lambda _{\phi }$)-plane negative” spin-alignment hypothesis for 8 TeV. Those intervals not measured in the analysis at low $p_{\text {T}}$, high rapidity are also excluded here

$p_{\text {T}}$ [GeV]	Absolute Rapidity Range							
	0.00–0.25	0.25–0.50	0.50–0.75	0.75–1.00	1.00–1.25	1.25–1.50	1.50–1.75	1.75–2.00
8.00–8.50	0.985	0.957	0.936	–	–	–	–	–
8.50–9.00	0.982	0.950	0.926	–	–	–	–	–
9.00–9.50	0.982	0.950	0.926	–	–	–	–	–
9.50–10.00	0.983	0.952	0.929	–	–	–	–	–
10.00–10.50	0.984	0.954	0.932	0.916	0.905	0.898	0.894	0.891
10.50–11.00	0.985	0.956	0.935	0.919	0.909	0.903	0.899	0.895
11.00–11.50	0.985	0.959	0.938	0.923	0.913	0.907	0.903	0.900
11.50–12.00	0.986	0.961	0.941	0.927	0.918	0.911	0.908	0.905
12.00–12.50	0.987	0.963	0.944	0.931	0.922	0.916	0.912	0.909
12.50–13.00	0.988	0.965	0.947	0.934	0.925	0.920	0.916	0.913
13.00–14.00	0.988	0.967	0.951	0.939	0.930	0.925	0.922	0.919
14.00–15.00	0.990	0.971	0.955	0.944	0.937	0.932	0.929	0.927
15.00–16.00	0.991	0.974	0.960	0.950	0.943	0.938	0.936	0.934
16.00–17.00	0.991	0.976	0.963	0.954	0.948	0.944	0.941	0.939
17.00–18.00	0.992	0.978	0.967	0.958	0.952	0.949	0.946	0.945
18.00–20.00	0.993	0.981	0.971	0.963	0.958	0.954	0.952	0.951
20.00–22.00	0.994	0.984	0.975	0.969	0.964	0.961	0.959	0.958
22.00–24.00	0.995	0.986	0.979	0.973	0.969	0.967	0.965	0.964
24.00–26.00	0.996	0.988	0.982	0.977	0.973	0.971	0.970	0.969
26.00–30.00	0.996	0.990	0.985	0.981	0.978	0.976	0.975	0.974
30.00–35.00	0.997	0.992	0.988	0.985	0.983	0.982	0.981	0.980
35.00–40.00	0.998	0.994	0.991	0.989	0.987	0.986	0.985	0.985
40.00–60.00	0.999	0.996	0.994	0.992	0.991	0.991	0.990	0.990
60.00–110.00	0.999	0.998	0.997	0.997	0.996	0.996	0.996	0.996

**Table 22:** Mean weight correction factor for $\psi (2\mathrm {S})$ under the “longitudinal” spin-alignment hypothesis for 8 TeV. Those intervals not measured in the analysis at low $p_{\text {T}}$, high rapidity are also excluded here

$p_{\text {T}}$ [GeV]	Absolute Rapidity Range							
	0.00–0.25	0.25–0.50	0.50–0.75	0.75–1.00	1.00–1.25	1.25–1.50	1.50–1.75	1.75–2.00
8.00–8.50	0.672	0.677	0.686	–	–	–	–	–
8.50–9.00	0.674	0.680	0.689	–	–	–	–	–
9.00–9.50	0.677	0.682	0.691	–	–	–	–	–
9.50–10.00	0.680	0.684	0.692	–	–	–	–	–
10.00–10.50	0.683	0.688	0.695	0.702	0.709	0.713	0.717	0.721
10.50–11.00	0.687	0.692	0.698	0.705	0.710	0.715	0.718	0.722
11.00–11.50	0.692	0.695	0.701	0.708	0.714	0.716	0.718	0.725
11.50–12.00	0.695	0.698	0.704	0.710	0.715	0.718	0.721	0.725
12.00–12.50	0.700	0.703	0.708	0.713	0.718	0.721	0.723	0.728
12.50–13.00	0.704	0.706	0.711	0.716	0.721	0.722	0.726	0.730
13.00–14.00	0.710	0.713	0.717	0.722	0.725	0.727	0.730	0.733
14.00–15.00	0.719	0.721	0.724	0.728	0.731	0.733	0.736	0.738
15.00–16.00	0.727	0.728	0.732	0.735	0.737	0.740	0.741	0.743
16.00–17.00	0.735	0.737	0.739	0.742	0.743	0.746	0.748	0.750
17.00–18.00	0.742	0.744	0.746	0.750	0.750	0.753	0.755	0.755
18.00–20.00	0.753	0.754	0.756	0.759	0.760	0.761	0.762	0.765
20.00–22.00	0.767	0.768	0.769	0.771	0.773	0.773	0.775	0.775
22.00–24.00	0.779	0.779	0.782	0.783	0.784	0.785	0.785	0.788
24.00–26.00	0.791	0.791	0.793	0.794	0.793	0.795	0.795	0.795
26.00–30.00	0.805	0.804	0.806	0.807	0.808	0.809	0.809	0.811
30.00–35.00	0.823	0.823	0.824	0.824	0.826	0.826	0.828	0.828
35.00–40.00	0.841	0.841	0.840	0.842	0.843	0.842	0.843	0.843
40.00–60.00	0.866	0.867	0.866	0.868	0.868	0.866	0.868	0.870
60.00–110.00	0.905	0.906	0.906	0.909	0.907	0.903	0.906	0.905

**Table 23:** Mean weight correction factor for $\psi (2\mathrm {S})$ under the “transverse zero” spin-alignment hypothesis for 8 TeV. Those intervals not measured in the analysis at low $p_{\text {T}}$, high rapidity are also excluded here

$p_{\text {T}}$ [GeV]	Absolute Rapidity Range							
	0.00–0.25	0.25–0.50	0.50–0.75	0.75–1.00	1.00–1.25	1.25–1.50	1.50–1.75	1.75–2.00
8.00–8.50	1.325	1.316	1.301	–	–	–	–	–
8.50–9.00	1.321	1.311	1.295	–	–	–	–	–
9.00–9.50	1.316	1.307	1.291	–	–	–	–	–
9.50–10.00	1.310	1.301	1.288	–	–	–	–	–
10.00–10.50	1.303	1.295	1.283	1.272	1.261	1.254	1.249	1.244
10.50–11.00	1.296	1.289	1.278	1.267	1.259	1.252	1.247	1.241
11.00–11.50	1.289	1.283	1.273	1.262	1.254	1.250	1.246	1.238
11.50–12.00	1.282	1.276	1.267	1.258	1.251	1.246	1.242	1.236
12.00–12.50	1.274	1.270	1.261	1.253	1.247	1.242	1.239	1.233
12.50–13.00	1.267	1.263	1.256	1.248	1.242	1.239	1.235	1.230
13.00–14.00	1.257	1.254	1.247	1.241	1.236	1.232	1.229	1.225
14.00–15.00	1.244	1.241	1.236	1.230	1.227	1.223	1.220	1.217
15.00–16.00	1.232	1.230	1.225	1.221	1.217	1.215	1.213	1.211
16.00–17.00	1.221	1.218	1.215	1.211	1.209	1.206	1.204	1.202
17.00–18.00	1.210	1.208	1.206	1.202	1.200	1.197	1.195	1.195
18.00–20.00	1.197	1.195	1.193	1.190	1.188	1.187	1.186	1.184
20.00–22.00	1.180	1.179	1.177	1.175	1.173	1.172	1.171	1.171
22.00–24.00	1.165	1.165	1.163	1.162	1.161	1.159	1.159	1.157
24.00–26.00	1.153	1.153	1.151	1.150	1.150	1.149	1.149	1.149
26.00–30.00	1.138	1.139	1.138	1.136	1.136	1.135	1.135	1.133
30.00–35.00	1.121	1.121	1.120	1.119	1.119	1.118	1.117	1.117
35.00–40.00	1.105	1.104	1.105	1.104	1.103	1.104	1.103	1.103
40.00–60.00	1.084	1.083	1.084	1.083	1.083	1.084	1.083	1.081
60.00–110.00	1.056	1.055	1.055	1.053	1.054	1.057	1.055	1.056

**Table 24:** Mean weight correction factor for $\psi (2\mathrm {S})$ under the “transverse positive” spin-alignment hypothesis for 8 TeV. Those intervals not measured in the analysis at low $p_{\text {T}}$, high rapidity are also excluded here

$p_{\text {T}}$ [GeV]	Absolute Rapidity Range							
	0.00–0.25	0.25–0.50	0.50–0.75	0.75–1.00	1.00–1.25	1.25–1.50	1.50–1.75	1.75–2.00
8.00–8.50	2.029	2.023	2.022	–	–	–	–	–
8.50–9.00	1.620	1.620	1.618	–	–	–	–	–
9.00–9.50	1.504	1.504	1.502	–	–	–	–	–
9.50–10.00	1.444	1.444	1.443	–	–	–	–	–
10.00–10.50	1.405	1.405	1.404	1.404	1.402	1.401	1.400	1.500
10.50–11.00	1.377	1.377	1.376	1.375	1.375	1.373	1.373	1.443
11.00–11.50	1.354	1.354	1.354	1.352	1.351	1.353	1.353	1.403
11.50–12.00	1.336	1.336	1.335	1.334	1.335	1.334	1.333	1.375
12.00–12.50	1.320	1.320	1.320	1.319	1.319	1.319	1.318	1.351
12.50–13.00	1.306	1.307	1.306	1.305	1.304	1.306	1.304	1.331
13.00–14.00	1.289	1.289	1.289	1.288	1.288	1.288	1.287	1.308
14.00–15.00	1.268	1.269	1.268	1.267	1.268	1.267	1.266	1.281
15.00–16.00	1.251	1.251	1.250	1.251	1.251	1.250	1.250	1.261
16.00–17.00	1.236	1.236	1.236	1.236	1.236	1.235	1.235	1.242
17.00–18.00	1.223	1.222	1.223	1.222	1.223	1.221	1.221	1.227
18.00–20.00	1.206	1.206	1.206	1.205	1.206	1.206	1.206	1.208
20.00–22.00	1.186	1.186	1.186	1.186	1.186	1.186	1.185	1.187
22.00–24.00	1.170	1.171	1.170	1.170	1.170	1.170	1.170	1.169
24.00–26.00	1.157	1.157	1.156	1.156	1.157	1.157	1.157	1.158
26.00–30.00	1.141	1.142	1.141	1.141	1.141	1.140	1.141	1.140
30.00–35.00	1.122	1.122	1.122	1.122	1.122	1.122	1.121	1.121
35.00–40.00	1.106	1.105	1.106	1.106	1.105	1.106	1.105	1.105
40.00–60.00	1.085	1.084	1.085	1.084	1.084	1.085	1.084	1.083
60.00–110.00	1.056	1.055	1.055	1.054	1.054	1.057	1.055	1.056

## Appendix: Spin-alignment correction factors

The measurement presented here assumes an unpolarized spin-alignment hypothesis for determining the correction factor. In principle, the polarization may be non-zero and may vary with $p_{\text {T}}$. In order to correct these measurements when well-measured $J/\psi$ and $\psi (2\mathrm {S})$ polarizations are determined, a set of correction factors are provided in Tables 4, 5, 6, 7, 8, 9, 10, 11, 12, 13, 14, 15 for the 7 TeV data, and in the Tables 16, 17, 18, 19, 20, 21, 22, 23, 24, 25, 26, 27 for the 8 TeV data. These tables are created by altering the spin-alignment hypothesis for either the $J/\psi$ or $\psi (2\mathrm {S})$ meson and then determining the ratio of the mean sum-of-weights of the new hypotheses to the original flat hypothesis. The mean weight is calculated from all the events in each dimuon $p_{\text {T}}$ and rapidity analysis bin, selecting those dimuons within $\pm 2\sigma$ of the $\psi$ fitted mean mass position. The choice of spin-alignment hypothesis for each $\psi$ meson has negligible effect on the results of the other $\psi$ meson, and therefore these possible permutations are not considered. The definitions of each of the spin-alignment scenarios, which are given in the caption to the table, are defined in Table 1.

**Table 25:** Mean weight correction factor for $\psi (2\mathrm {S})$ under the “transverse negative” spin-alignment hypothesis for 8 TeV. Those intervals not measured in the analysis at low $p_{\text {T}}$, high rapidity are also excluded here

$p_{\text {T}}$ [GeV]	Absolute Rapidity Range							
	0.00–0.25	0.25–0.50	0.50–0.75	0.75–1.00	1.00–1.25	1.25–1.50	1.50–1.75	1.75–2.00
8.00–8.50	0.995	0.986	0.970	–	–	–	–	–
8.50–9.00	1.116	1.102	1.081	–	–	–	–	–
9.00–9.50	1.170	1.156	1.133	–	–	–	–	–
9.50–10.00	1.199	1.185	1.163	–	–	–	–	–
10.00–10.50	1.215	1.202	1.182	1.163	1.146	1.135	1.127	1.075
10.50–11.00	1.225	1.212	1.194	1.175	1.161	1.150	1.142	1.098
11.00–11.50	1.230	1.218	1.201	1.184	1.170	1.161	1.155	1.114
11.50–12.00	1.232	1.222	1.206	1.190	1.178	1.169	1.162	1.127
12.00–12.50	1.232	1.223	1.208	1.194	1.182	1.174	1.168	1.137
12.50–13.00	1.231	1.223	1.210	1.196	1.185	1.178	1.172	1.146
13.00–14.00	1.228	1.220	1.209	1.197	1.188	1.181	1.176	1.154
14.00–15.00	1.221	1.215	1.206	1.196	1.188	1.182	1.177	1.161
15.00–16.00	1.214	1.209	1.200	1.193	1.186	1.181	1.177	1.165
16.00–17.00	1.206	1.202	1.195	1.188	1.183	1.178	1.175	1.166
17.00–18.00	1.198	1.195	1.189	1.183	1.179	1.174	1.171	1.165
18.00–20.00	1.188	1.184	1.180	1.175	1.171	1.168	1.166	1.161
22.00–24.00	1.161	1.160	1.156	1.154	1.151	1.149	1.149	1.145
24.00–26.00	1.150	1.149	1.146	1.144	1.143	1.141	1.141	1.140
26.00–30.00	1.136	1.136	1.134	1.132	1.131	1.129	1.129	1.127
30.00–35.00	1.119	1.119	1.117	1.117	1.115	1.115	1.113	1.113
35.00–40.00	1.104	1.103	1.103	1.102	1.101	1.101	1.100	1.100
40.00–60.00	1.084	1.083	1.083	1.082	1.082	1.083	1.082	1.080
60.00–110.00	1.056	1.054	1.055	1.053	1.054	1.057	1.055	1.055

**Table 26:** Mean weight correction factor for $\psi (2\mathrm {S})$ under the “off-($\lambda _{\theta }$–$\lambda _{\phi }$)-plane positive” spin-alignment hypothesis for 8 TeV. Those intervals not measured in the analysis at low $p_{\text {T}}$, high rapidity are also excluded here

$p_{\text {T}}$ [GeV]	Absolute Rapidity Range							
	0.00–0.25	0.25–0.50	0.50–0.75	0.75–1.00	1.00–1.25	1.25–1.50	1.50–1.75	1.75–2.00
8.00–8.50	1.018	1.053	1.081	–	–	–	–	–
8.50–9.00	1.021	1.062	1.095	–	–	–	–	–
9.00–9.50	1.021	1.062	1.096	–	–	–	–	–
9.50–10.00	1.020	1.060	1.094	–	–	–	–	–
10.00–10.50	1.020	1.057	1.089	1.114	1.130	1.140	1.146	1.145
10.50–11.00	1.018	1.055	1.085	1.108	1.124	1.133	1.139	1.142
11.00–11.50	1.017	1.052	1.080	1.102	1.117	1.127	1.133	1.137
11.50–12.00	1.017	1.049	1.076	1.096	1.111	1.120	1.126	1.132
12.00–12.50	1.016	1.046	1.072	1.091	1.105	1.113	1.119	1.125
12.50–13.00	1.015	1.043	1.068	1.086	1.099	1.108	1.112	1.119
13.00–14.00	1.013	1.040	1.062	1.079	1.091	1.099	1.104	1.111
14.00–15.00	1.012	1.036	1.056	1.071	1.082	1.089	1.093	1.099
15.00–16.00	1.011	1.032	1.050	1.064	1.073	1.080	1.084	1.090
16.00–17.00	1.010	1.029	1.045	1.057	1.067	1.072	1.076	1.081
17.00–18.00	1.009	1.026	1.041	1.052	1.060	1.065	1.068	1.073
18.00–20.00	1.008	1.023	1.036	1.045	1.053	1.057	1.060	1.063
20.00–22.00	1.007	1.019	1.030	1.038	1.044	1.048	1.050	1.053
22.00–24.00	1.006	1.016	1.025	1.032	1.037	1.040	1.043	1.044
24.00–26.00	1.005	1.014	1.022	1.028	1.032	1.035	1.037	1.038
26.00–30.00	1.004	1.012	1.018	1.023	1.026	1.029	1.030	1.031
30.00–35.00	1.003	1.009	1.014	1.017	1.020	1.022	1.023	1.023
35.00–40.00	1.002	1.007	1.010	1.013	1.015	1.017	1.017	1.018
40.00–60.00	1.002	1.004	1.007	1.009	1.010	1.011	1.012	1.012
60.00–110.00	1.001	1.002	1.003	1.004	1.004	1.005	1.005	1.005

**Table 27:** Mean weight correction factor for $\psi (2\mathrm {S})$ under the “off-($\lambda _{\theta }$–$\lambda _{\phi }$)-plane negative” spin-alignment hypothesis for 8 TeV. Those intervals not measured in the analysis at low $p_{\text {T}}$, high rapidity are also excluded here

$p_{\text {T}}$ [GeV]	Absolute Rapidity Range							
	0.00–0.25	0.25–0.50	0.50–0.75	0.75–1.00	1.00–1.25	1.25–1.50	1.50–1.75	1.75–2.00
8.00–8.50	0.983	0.952	0.931	–	–	–	–	–
8.50–9.00	0.980	0.945	0.920	–	–	–	–	–
9.00–9.50	0.980	0.945	0.919	–	–	–	–	–
9.50–10.00	0.981	0.946	0.921	–	–	–	–	–
10.00–10.50	0.981	0.949	0.924	0.908	0.897	0.891	0.887	0.888
10.50–11.00	0.982	0.951	0.928	0.912	0.901	0.895	0.891	0.890
11.00–11.50	0.983	0.953	0.931	0.916	0.906	0.899	0.895	0.893
11.50–12.00	0.984	0.956	0.934	0.919	0.910	0.903	0.900	0.896
12.00–12.50	0.985	0.958	0.937	0.923	0.914	0.908	0.904	0.900
12.50–13.00	0.986	0.960	0.940	0.927	0.918	0.911	0.908	0.904
13.00–14.00	0.987	0.963	0.945	0.932	0.923	0.917	0.914	0.910
14.00–15.00	0.988	0.967	0.950	0.938	0.930	0.925	0.922	0.917
15.00–16.00	0.989	0.970	0.955	0.944	0.936	0.931	0.928	0.924
16.00–17.00	0.990	0.973	0.959	0.949	0.941	0.937	0.934	0.931
17.00–18.00	0.991	0.975	0.962	0.953	0.946	0.943	0.940	0.936
18.00–20.00	0.992	0.978	0.967	0.958	0.953	0.949	0.946	0.944
20.00–22.00	0.993	0.981	0.972	0.965	0.960	0.956	0.955	0.952
22.00–24.00	0.994	0.984	0.976	0.970	0.965	0.963	0.961	0.960
24.00–26.00	0.995	0.986	0.979	0.974	0.970	0.967	0.966	0.965
26.00–30.00	0.996	0.989	0.983	0.978	0.975	0.973	0.972	0.971
30.00–35.00	0.997	0.991	0.987	0.983	0.981	0.979	0.978	0.978
35.00–40.00	0.998	0.993	0.990	0.987	0.985	0.984	0.983	0.983
40.00–60.00	0.998	0.996	0.993	0.992	0.990	0.989	0.989	0.989
60.00–110.00	0.999	0.998	0.997	0.996	0.996	0.995	0.995	0.995

## Footnotes

1 ATLAS uses a right-handed coordinate system with its origin at the nominal interaction point (IP) in the centre of the detector and the *z*-axis along the beam pipe. The *x*-axis points from the IP to the centre of the LHC ring, and the *y*-axis points upward. Cylindrical coordinates $(r,\phi )$ are used in the transverse plane, $\phi$ being the azimuthal angle around the beam pipe. The pseudorapidity $\eta$ is defined in terms of the polar angle $\theta$ as $\eta =-\ln \tan (\theta /2)$ and the transverse momentum $p_\mathrm{T}$ is defined as $p_\mathrm{T}=p\sin \theta$. The rapidity is defined as $y=0.5\ln \left[ \left( E + p_z \right) / \left( E - p_z \right) \right]$, where *E* and $p_z$ refer to energy and longitudinal momentum, respectively. The $\eta$–$\phi$ distance between two particles is defined as $\Delta R=\sqrt{(\Delta \eta )^2 + (\Delta \phi )^2}$.

2 The Crystal Ball function is given by:
$$B(x;\alpha ,n,\bar{x},\sigma ) = N \cdot {\left\{ \begin{array}{ll} \exp \left( - \frac{(x - \bar{x})^2}{2 \sigma ^2}\right) , &{} \text{ for } \frac{x - \bar{x}}{\sigma } > -\alpha \\ A \cdot \left( A' - \frac{x - \bar{x}}{\sigma }\right) ^{-n}, &{} \text{ for } \frac{x - \bar{x}}{\sigma } \leqslant -\alpha \end{array}\right. }$$
where $$A = \left( \frac{n}{\left| \alpha \right| }\right) ^n \cdot \exp \left( - \frac{\left| \alpha \right| ^2}{2}\right) , A' = \frac{n}{\left| \alpha \right| } - \left| \alpha \right| $$
